# Conservation of magnetite biomineralization genes in all domains of life and implications for magnetic sensing

**DOI:** 10.1073/pnas.2108655119

**Published:** 2022-01-10

**Authors:** M. Renee Bellinger, Jiandong Wei, Uwe Hartmann, Hervé Cadiou, Michael Winklhofer, Michael A. Banks

**Affiliations:** ^a^Coastal Oregon Marine Experiment Station, Department Fisheries and Wildlife, Hatfield Marine Science Center, Oregon State University, Newport, OR 97365;; ^b^Experimental Physics Department, Saarland University, D-66041 Saarbruecken, Germany;; ^c^Institut des Neurosciences Cellulaires et Intégratives (INCI), Centre National de la Recherche Scientifique UPR3212, F-67100 Strasbourg, France;; ^d^Institute of Biology and Environmental Science, University of Oldenburg, D-26129 Oldenburg, Germany;; ^e^Research Center Neurosensory Science, University of Oldenburg, D-26111 Oldenburg, Germany

**Keywords:** endosymbiosis, eukaryogenesis, exaptation, magnetoreception, sensory organelle

## Abstract

Animals use geomagnetic fields for navigational cues, yet the sensory mechanism underlying magnetic perception remains poorly understood. One idea is that geomagnetic fields are physically transduced by magnetite crystals contained inside specialized receptor cells, but evidence for intracellular, biogenic magnetite in eukaryotes is scant. Certain bacteria produce magnetite crystals inside intracellular compartments, representing the most ancient form of biomineralization known and having evolved prior to emergence of the crown group of eukaryotes, raising the question of whether magnetite biomineralization in eukaryotes and prokaryotes might share a common evolutionary history. Here, we discover that salmonid olfactory epithelium contains magnetite crystals arranged in compact clusters and determine that genes differentially expressed in magnetic olfactory cells, contrasted to nonmagnetic olfactory cells, share ancestry with an ancient prokaryote magnetite biomineralization system, consistent with exaptation for use in eukaryotic magnetoreception. We also show that 11 prokaryote biomineralization genes are universally present among a diverse set of eukaryote taxa and that nine of those genes are present within the Asgard clade of archaea Lokiarchaeota that affiliates with eukaryotes in phylogenomic analysis. Consistent with deep homology, we present an evolutionary genetics hypothesis for magnetite formation among eukaryotes to motivate convergent approaches for examining magnetite-based magnetoreception, molecular origins of matrix-associated biomineralization processes, and eukaryogenesis.

## Significance

We present a model of biogenic magnetite formation in eukaryotes and hypothesize this genetic mechanism is used by broad forms of life for geomagnetic sensory perception. Countering previous assertions that salmon olfactory tissues lack biogenic magnetite, we determine that it is present in the form of compact crystal clusters and show that a subset of genes differentially expressed in candidate magnetoreceptor cells, compared to nonmagnetic olfactory cells, are distant homologs to a core suite of genes utilized by magnetotactic bacteria for magnetite biomineralization. This same core gene suite is common to a broad array of eukaryotes and the Asgard clade archaea Lokiarchaeta. Findings have implications for revising our understanding of eukaryote magnetite biomineralization, sensory perception of magnetic fields, and eukaryogenesis.

Diverse animals utilize the Earth’s magnetic field for orientation and navigation cues; however, the receptor mechanism that underlies this sensory ability remains a fundamental question in sensory biology (1–3). A leading hypothesis posits that specialized sensory organelles containing crystals of magnetite physically interact with Earth‐strength magnetic fields to transduce geomagnetic information into neural signals (1, 4–7). These crystals are predicted to be similar in shape and size (4–7) to iron mineral crystals biosynthesized by magnetotactic bacteria (MTB) for use in magnetotaxis, passive alignment to geomagnetic fields (8). MTB are the most ancient and simple organisms known to biomineralize (7–9), with biologically controlled iron-based (Fe_3_O_4_ and Fe_3_S_4_) iron mineral formation in the domain Bacteria proposed to have originated ∼3 to ∼2 gigaannum (Ga) (9–11). Magnetite biomineralization thus predates the emergence of the crown group of eukaryotes (∼1.8 to 1.2 Ga), based on the fossil record and molecular clock estimates (7, 9–12). Like other forms of nonskeletal biomineralization, formation of crystals occurs in intracellular compartments bounded by membranes, underpinned by local expression of genes that guide precipitation (13). The mechanisms that control magnetite biomineralization in prokaryotes have been studied for decades, and numerous associated proteins are well characterized (14–17).

Presence of magnetite in eukaryotes has mainly been affirmed through magnetic remanence measurements in magnetosensitive species, e.g., honeybees, birds, mice, fish (reviewed by ref. 6), yet direct evidence for intracellular magnetite is scant, the evolutionary origins are poorly understood, and no magnetite-based receptor has been confirmed in situ (1, 18, 19). Iron-rich structures detected in the upper beak of pigeons were once proposed as magnetoreceptors (20) but later were identified as phagocytosed debris in cells presenting major histocompatibility complex II, probably macrophages (18). Still, multiple lines of evidence support a “universal” magnetite-based magnetoreceptor (4, 5): The trigeminal nerve of fish exhibits neural responses to magnetic treatment (4), neurons associated with the avian trigeminal brainstem complex show magnetic activation (2), and behavioral responses to pulse magnetization are exhibited by birds, sea turtles, and bats (21–23). Thus, the magnetite hypothesis for geomagnetic receptivity holds and is believed to provide sensory information that differs from the cryptochrome-based model, which is unaffected by magnetic pulse (3).

Pacific and Atlantic salmon (*Oncorhynchus* and *Salmo*) possess an innate guidance mechanism utilized for long-distance migration and homing to natal rivers (24). Navigational cues include geomagnetic intensity and inclination, as shown by exposing juvenile salmon to simulated magnetic displacements (25, 26). Although magnetite is present in salmon tissues, no deposits have been directly associated with sensory transduction and in most cases are unlikely to represent the magnetoreceptor site (6). An important exception is occurrence of magnetite in olfactory epithelial tissue (refs. 1, 5, and 27; but see ref. 19), innervated by the magnetically responsive superficial ophthalmic branch of the trigeminal nerve (4). We extend the hypothesis that magnetite-containing cells have a universal genetic basis and role in magnetoreception through 1) in situ magnetic measurements, microscopies, and transcriptomic characterization of magnetite-containing cells of salmonids; 2) assessing whether magnetite biomineralization in eukaryotes could have ancient prokaryotic origins by comparing the genome contents of a salmon, 12 additional eukaryotes, and one archaea against an MTB magnetosome protein sequence database; and 3) proposing an evolutionary genetics hypothesis for eukaryote biomineralization and magnetoreception predicated on transcriptomic and comparative genomic findings.

## Results

### Salmonid Candidate Magnetoreceptors.

**Fig. 1.**
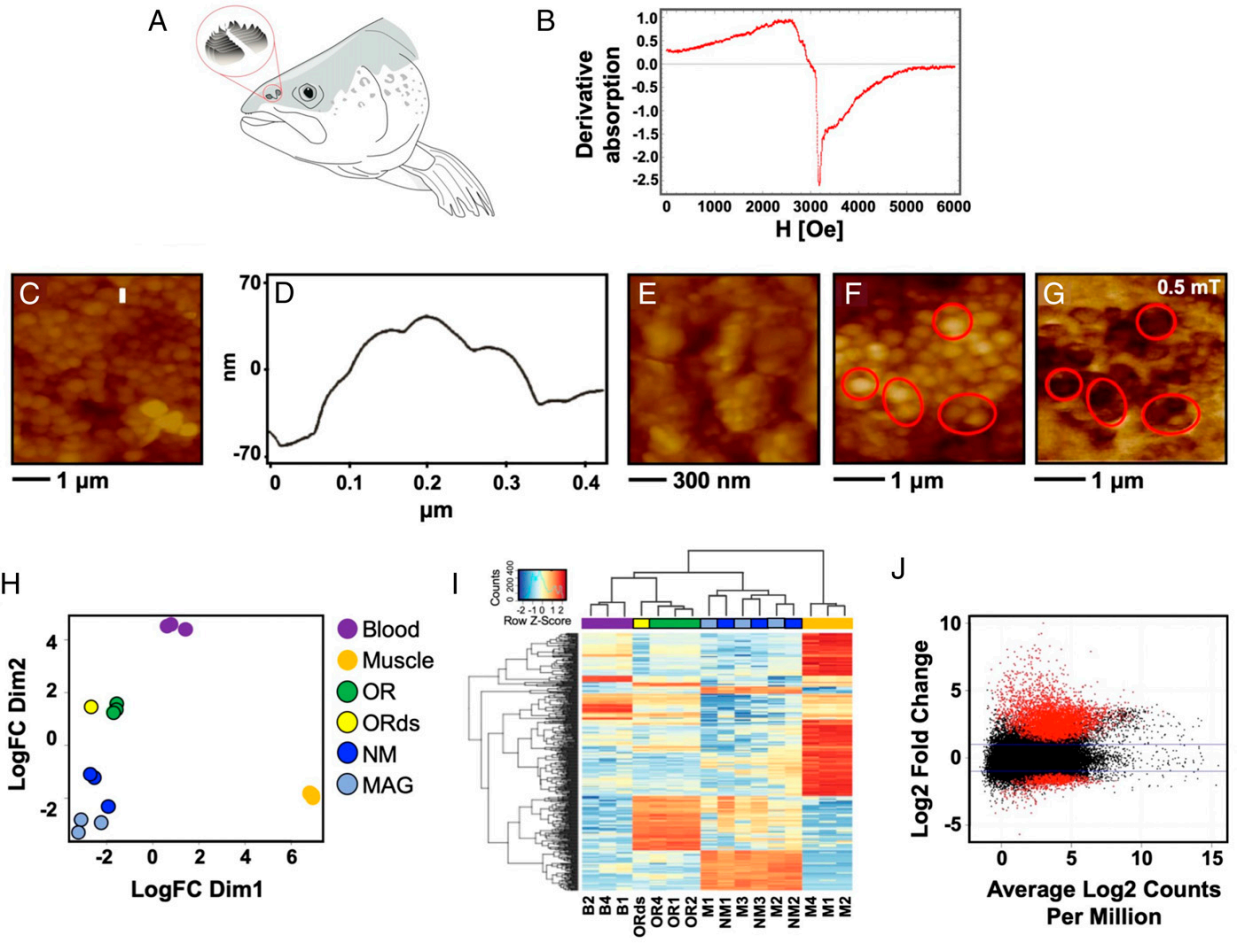
Candidate magnetoreceptor cell characteristics. (*A*) Schematic representation of a salmonid head showing OR location. (*B*) Broad electron spin resonance spectrum of rainbow trout (*O. mykiss*) ORs demonstrates presence of ferromagnetic material. The sharp edge at a magnetic field strength H = 3 kOe corresponds to a paramagnetic signal (*SI Appendix*, Fig. S1). (*C*, *E*, and *F*) AFM images of magnetite clusters extracted from Atlantic salmon (*S. salar*) ORs (*SI Appendix*, Fig. S2). (*D*) Dimensional profile of the magnetite cluster (*x* axis) and maximum diameter of individual magnetite particles (*y* axis) marked by the white line in (*C*). (*E*) Individual particles can be visualized under higher magnification. (*G*) Magnetic force microscopy image obtained at 0.5 mT; image directly corresponds to *F*. (*H–J*) Chinook salmon (*O. tshawytscha)* transcriptome profiles of three blood, muscle, and whole OR samples obtained from three fish (*n* = 9 transcriptomes), a single pair of deep-sequenced ORs (ORds) sampled from a fourth fish (*n* = 1 transcriptome), and MAG and NM cells obtained through three replicate MAG cell isolation experiments, each using dissociated ORs from 3 to 5 fish (*n* = 3 MAG and *n* = 3 NM transcriptomes). (*H*) Multidimensional scaling plot and (*I*) heatmap of top 500 most abundantly expressed genes across the 16 transcriptomes. ORs in the color keys are demarcated with dark outlines. (*J*) M (log ratio) versus A (mean average) plot of the log2 fold ratio of modeled gene expression values (*y* axis) and average log2 counts per million (*x* axis) between magnetic (negative *y* axis) and nonmagnetic (positive *y* axis) cell isolates, with red dots indicating DEGs (at FDR < 0.05) and black dots indicating no significant difference in gene expression.

The physical properties of magnetite in salmon olfactory epithelium were characterized using a combination of ferromagnetic resonance spectrum (FMR) and atomic and magnetic force microscopies (AFM/MFM). The FMR analysis, conducted on intact olfactory rosette (OR) tissues (Fig. 1*A*), provides in situ information relating to the size and physical arrangement of magnetite particles. The rainbow trout (*Oncorhynchus mykiss*) broad FMR spectrum (Fig. 1*B*; *SI Appendix*, Fig. S1) seen in the electron spin resonance spectrum is different from that reported for linear chains of magnetosome crystals in MTB and rather resembles the FMR spectra of strongly interacting magnetic particle systems (28). Consistent with that finding, visualized under AFM, magnetic particles extracted from digests of Atlantic salmon (*Salmo salar*) olfactory epithelium appear as uniformly sized and ellipsoid shaped clusters, with each cluster containing a compact arrangement of individual particles. Clusters range in size from ∼200 to ∼300 nm (Fig. 1 *C–G*) and are estimated to contain ∼100 to 200 individual particles with diameters that range from approximately ∼30 to 60 nm. As an example, a profile of a single cluster (Fig. 1*D*) marked by the white bar in Fig. 1*C*, is ∼300 nm in diameter and contains crystals with a maximum diameter of ∼60 nm. Individual crystals can also be visualized in the higher resolution image shown in Fig. 1*E*. Using images taken from a different sample location, to demonstrate the magnetic properties of particle clusters, a switch from AFM (Fig. 1*F*) to MFM measurements performed in a near-zero field show an attractive interaction between the magnetic probe tip and the magnetite, which results from magnetostatic interactions and is indicated by a dark contrast (Fig. 1*G*; *SI Appendix*, Fig. S2). Our images of magnetite are strikingly similar to those obtained by Diebel et al. (5) (see their figure 2), who used confocal microscopy and AFM/MFM to visualize a cluster of intracellular magnetite in a rainbow trout olfactory epithelium cell. In our case, we can rule out bacteria and commercially prepared magnetite contaminants by differences in particle size and aggregation patterns visualized by AFM/MFM (*SI Appendix*, Figs. S2 and S3).

After confirming the presence of biogenic magnetite in salmonid olfactory epithelium, we then determined candidate magnetoreceptor genes of Chinook salmon (*Oncorhynchus tshawytscha*) by contrasting transcriptome profiles of magnetic (MAG) and nonmagnetic (NM) olfactory cells and blood and muscle tissues. Briefly, three replicate MAG cell isolation experiments were conducted by dissociating OR cells, followed by collection of MAG cells using a magnet with a pointed tip placed on the outside, upper portion of the sample vial and allowing the NM cells to settle to the bottom of the vial through gravitational forces. The pellet of MAG cells that accumulated inside the vial at the tip of the magnet and NM cells from the bottom of the vial were aspirated and transferred into new vials for messenger RNA (mRNA) isolation. Because of MAG cell scarcity, three to five sets of ORs were combined for each cell isolation experiment. The MAG and NM samples, plus three sets of ORs, blood, and muscle tissues from three additional fish and a set of ORs from a fourth fish (for a total of 16 transcriptomes), were subjected to Illumina sequencing for transcriptome profiling. Adjusting for false discovery rates (FDRs) < 0.05, this experiment revealed 610 differentially expressed genes (DEGs) more highly expressed in the MAG relative to the NM cell type and considerably greater difference between MAG and blood and muscle tissues (Fig. 1 *H–J*; *SI Appendix*, Fig. S4 and Table S1). In the latter two cases, >11,000 DEGs were more highly expressed in each binary comparison. Consistent with DEG results, multidimensional scaling plots show well separated clusters of points by tissue type or experimental condition (Fig. 1*H*). Two of the three MAG samples clustered together, positioned distinct from their NM sample counterparts, while the third MAG sample grouped between the other MAG samples and its NM experimental counterpart. The NM samples were positioned intermediate between the MAG and nontreated olfactory samples. A heatmap of the top 500 most variable genes shows that at this high level, samples from MAG and NM experimental trials group together (Fig. 1*I*), which masks expression differences between these two OR cell subtypes. Overall, differences in gene expression fold-differences and transcript abundance are less for the MAG–NM contrast compared to MAG–blood and MAG–muscle contrasts, as visualized in MA plots, in which red and black dots depict genes with significant or nonsignificant levels of expression, respectively (Fig. 1*J* MAG–NM contrast; MAG–blood and MAG–muscle contrasts available in *SI Appendix*, Fig. S4).

Discrete differential gene expression distinctions observed repeatedly when comparing MAG and NM cell findings in our study are only consistent with the conclusion that salmon olfactory tissue magnetic properties result from the intracellular presence of biogenic magnetite. With macrophages ruled out (*SI Appendix*), a random assortment of MAG material attached to NM cells could not provide the data observed here.

To broadly characterize the molecular functions of MAG cells, we relaxed the threshold FDR < 0.1 and focused on the 1,588 DEGs more highly expressed within the MAG sample contrasted to the NM sample. These candidate genes were overrepresented in 80 Gene Ontology (GO) categories, including anatomical structure and cell maturation/development, mitotic cell cycle, protein modification, protein binding, and bounding membrane of an organelle (Dataset S1). Among the DEGs were proteins involved in “iron uptake and transport” (14, <1% of DEGs) and “iron ion binding’” (6, <1% of DEGs), including ferritin. Also present were proteins associated with keywords “actin” (84, 5.3% of DEGs), “microtubule” (24, 1.5% of DEGs), and “cytoskeleton” (36, 2.3% of DEGs). These results are consistent with the production or maintenance of an organelle, possibly one produced through a cellular machinery process that somehow shares commonalities with mitosis and that involves iron.

### “Universal” Magnetosome Gene Homologs.

**Fig. 2.**
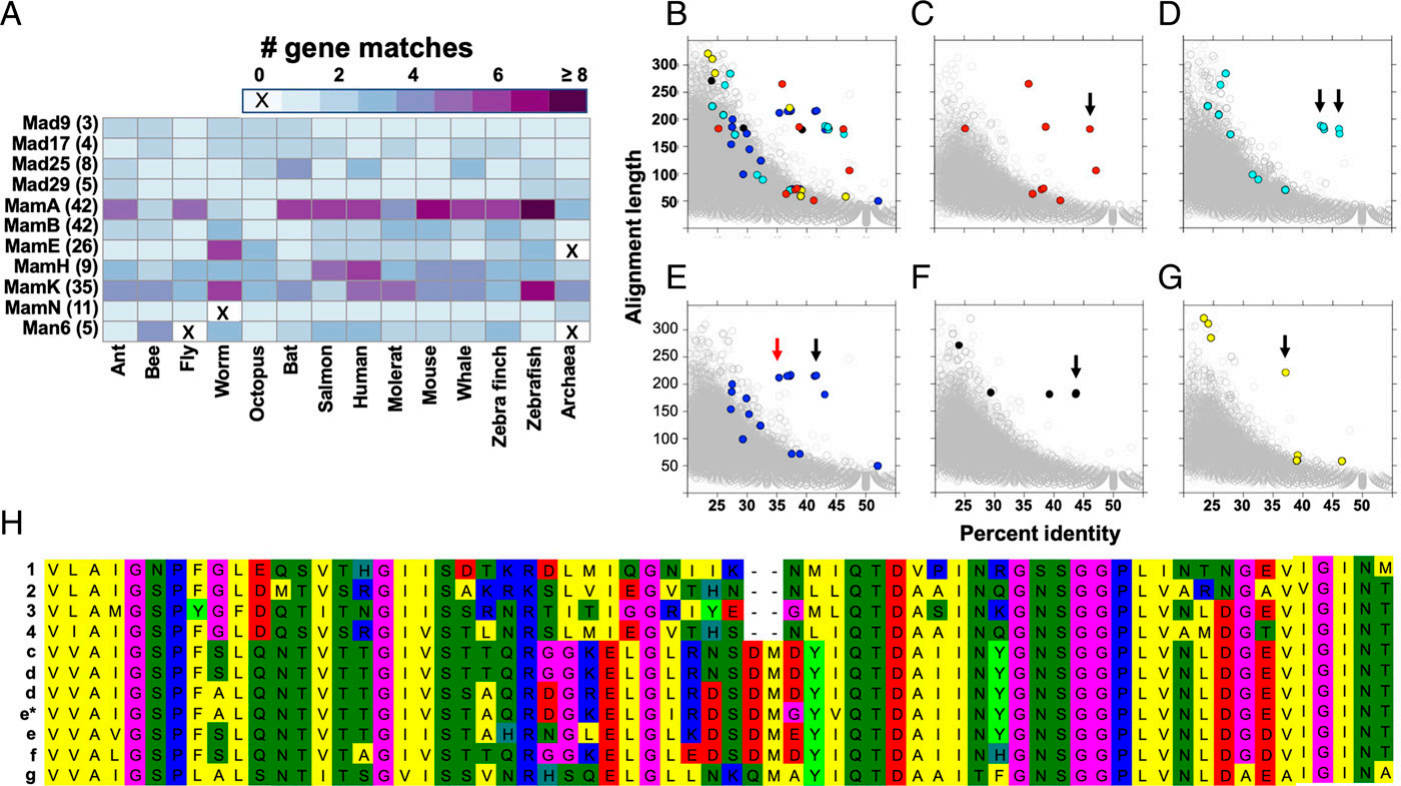
Comparative genomics. Data are presented for reciprocal BLASTp matches between magnetotactic bacterial biomineralization proteins and genome contents of eukaryotes and the archaea Lokiarchaeota. (*A*) Numbers of eukaryote proteins with reciprocal BLASTp match to 11 proteins known for involvement in prokaryote iron biomineralization (numbers of genes in prokaryote database in parenthesis). (*B–G*) Scatterplots of alignment lengths and percent identities scores for unidirectional BLASTp matches between genome contents of five magnetic responsive eukaryote taxa and the MTB magnetosome gene dataset (gray background circles). Proteins showing homology to the MTB gene MamE (HtrA-like serine protease) with E-value < 1 × 10e−5 are color highlighted. (*B*) All taxa (*C–G* combined), (*C*) zebra finch, *Taeniopygia guttata* (red); (*D*) naked mole-rat *Heterocephalus glaber* (cyan); (*E*) Chinook salmon, *O. tshawytscha* (blue); (*F*) little brown bat, *Myotis lucifugus* (black); and (*G*) honeybee, *Apis mellifera* (yellow). (*H*) A partial (66 amino acid) MamE alignment displays high levels of conservation across the five eukaryote taxa (*C–G*) and four MTB (1 to 4: UniprotKB accessions L0R6S4, *Desulfamplus magnetovallimortis;* C5JBP1, uncultured bacterium; A0A0F3GW16, *Candidatus Magnetobacterium bavaricum;* C5JAJ2, uncultured bacterium). Arrows in panels *C* to *G* point to the gene included in the multispecies alignment, with the red arrow indicating a gene differentially and more highly expressed in salmonid candidate magnetoreceptor cells, indicated by e* in the alignment. A full alignment is available from Dataset S4. Genome details are available from *SI Appendix*, Table S2.

To examine the hypothesis that genetic mechanisms controlling magnetite biomineralization in prokaryotes and eukaryotes might share common, ancient origins, we compared the genome contents of 13 eukaryotes (five protostomes and eight deuterostomes; *SI Appendix*, Table S2) to a database of magnetite biomineralization genes (Dataset S2). We found that 11 MTB magnetosome gene homologs (MGHs) are “universally present” (uMGHs) in eukaryotes, defined as having bidirectional Basic Local Alignment Search Tool protein (BLASTp) matches across at least 12 of the 13 animal genomes (>92%, Fig. 2*A* and Datasets S2 and S3). Furthermore, 9 of these 11 uMGHs were contained in genome contents of the Asgard archaea clade Lokiarchaeota (Fig. 2*A*; Datasets S2 and S3), which shows monophyly with eukaryotes (29, 30). The MamE homolog, an HTRA-like serine protease, exhibits exceptionally high levels of conservation in Chinook salmon and other magnetically sensitive animals (Fig. 2; Dataset S4 and *SI Appendix*).

A previous survey of MTB Nitrospirae and Proteobacteria genomes indicates they share a core set of five MTB magnetosome genes, MamABEKP (10). The 11 uMGHs identified in our study include four of these five core genes, with only MamP missing from eukaryotes (and Lokiarchaeota; Fig. 2*A*; Dataset S3). MamP contains an iron-binding residue with a role in iron oxidation (31), but this protein is not essential for crystal formation, possibly because of functional compensation by other magnetosome proteins (15, 31). These core genes, along with MamH and MamN (6 of the 11 uMGHs), are part of the MamAB operon (14–16), the only operon solely capable of supporting magnetite crystallization (14, 17). We found no support for the presence of MGHs belonging to three other magnetosome-associated, operon-like gene clusters, MamGFDC, MamXY, and mms6 (Datasets S2 and S3). Those gene clusters are generally present in magnetotactic Alphaproteobacteria (14, 17, 32) but absent from magnetotactic Deltaproteobacteria and Nitrospirae (10, 16, 33) (Datasets S2 and S3). Of the remaining five uMGHs, Mad9, 17, 25, 29 are present in genomes of magnetotactic Deltaproteobacteria and Nitrospirae, the latter also containing Man6 (10, 16). At a broader view, a meta-analysis of MTB genomes indicated that Mad genes are present in Nitrospirae, Omnitropha, and Deltaproteobacteria but absent from Proteobacteria classes Alpha, Eta, and Zeta and that Man genes are only contained in genomes of Nitrospirae (34). Thus, presence of the Man6 uMGH in eukaryote genomes, in conjunction with generally high proportions of eukaryote gene matches to individual Nitrospirae MTB proteins (*SI Appendix*, Table S3), is most parsimonious with a magnetite biomineralization gene transfer to eukaryotes having involved a Nitrospirae ancestor.

After identifying the 11 “universally conserved” uMGH proteins, we then cataloged their complete repertoire (homologs and paralogs) within genomes of zebrafish (*Danio rerio*) and Chinook salmon, which amounted to a total of 244 and 367 genes encoding uMGHs. Of those Chinook salmon genes, 332 matched to 181 zebrafish orthologs and corresponding Zebrafish Information Network (ZFIN) gene codes (35), a ∼45% reduction most likely explained by salmonid’s whole genome duplication event (36). In contrast, the zebrafish gene dataset was only marginally reduced (to 226 ZFIN gene codes) after accounting for a small number of paralogs. The number of fish genes encoding uMGHs varied across the 11 uMGH categories, with MamA, MamE, and MamK having the greatest number of matches (Table 1). Using PANTHER (37) and ZFIN gene codes to leverage the well-annotated zebrafish genome (35), notable protein classes included oxidoreductase, protein chaperones, matrix proteins, serine proteases, and transporters. Despite the diversity of protein classes, gene ontology analysis for these two sets of fish uMGHs indicated significant overrepresentation and exceptionally high fold-enrichment values across several categories; as an example, the molecular function term “protein folding chaperone” is 90× enriched in zebrafish and 63× enriched in Chinook salmon. Other notably enriched ontology categories include protein folding and refolding; divalent inorganic cation transmembrane transporter activity; four iron, four sulfur cluster binding; zinc ion transport, activity, and response; cellular response to heat (mostly heat shock proteins); and actin-based cell projection (*SI Appendix*, Table S4; hierarchical ontologies available from Dataset S5). Consistent with these findings, significantly overrepresented reactome pathways include zinc efflux and compartmentalization by the SLC30 family; signal transduction; laminin interactions; and the anaphase promoting complex/cyclosome, which regulates progression through the mitotic phase of the of the cell cycle (38). Several magnetite biomineralization proteins of bacteria have been functionally categorized, yet the roles of some proteins are not yet well understood, especially within the magnetotactic Nitrospirae and Deltaproteobacteria (Table 1). A list of genes encoding fish uMGHs, their ZFIN codes, and protein class annotations are provided in Dataset S6.

**Table 1.** Summary data for the complete repertoire of fish genes encoding distant homologs of 11 MTB biomineralization proteins

MTB protein name	No. uMGH: Z, C (no. unique)	No. annot. ZFINs Z, C	Eukaryote PANTHER protein classes (no. genes Z, C)	MTB biomineralization protein function
Mad17	4, 9 (6)	2, 3	G protein (1, 1); RNA methyltransferase (0, 1); protein modifying enzyme (0, 1); RNA metabolism protein (1, 0).	May be involved in production of crystals and/or crystal shape.
Mad25	6, 12 (7)	4, 6	Membrane traffic protein (2, 3); nonreceptor serine/threonine protein kinase (2, 2); protein-binding activity modulator (0, 1).	
Mad29	4, 6 (3)	1, 1	Transporter (1, 1).	
Mad9	2, 4 (3)	2, 2	Oxidoreductase (2, 2).	
MamA	89 (83), 144 (75)	41, 34	RNA splicing factor (1, 1); chaperone (11, 10); chromatin/chromatin-binding or regulatory protein (2, 1); general transcription factor (2, 1); membrane trafficking regulatory protein (3, 1); microtubule binding motor protein (2, 3); nonreceptor serine/threonine protein kinase (1, 0); nucleic acid metabolism protein (1, 1); primary active transporter (1, 1); protein-binding activity modulator (1, 1); protein modifying enzyme (4, 3); protein phosphatase (1, 1); scaffold/adaptor protein (1, 1); serine protease (1, 1); structural protein (1, 0); ubiquitin-protein ligase (8, 8).	Protein–protein interactions; multiprotein assembly site on the magnetosome.
MamB	12, 17 (11)	5, 6	G protein–coupled receptor (0, 1); nonreceptor serine/threonine protein kinase (0, 1); transporter (5, 4).	Membrane invagination; magnetosome membrane assembly; biomineralization.
MamE	66 (60), 58 (35)	43, 21	C2H2 zinc finger transcription factor (0, 1); actin or actin-binding cytoskeletal protein (1, 1); cell junction protein (1, 1); cytoskeletal protein (2, 3); general transcription factor (1, 0); guanyl-nucleotide exchange factor (3, 2); membrane trafficking regulatory protein (2, 0); oxidase (1, 0); protease (1, 0); protein phosphatase (2, 0); scaffold/adaptor protein (3, 5); secondary carrier transporter (1, 0); serine protease (21, 4); tight junction (4, 4).	Protein localization to the magnetosome membrane; membrane invagination and magnetosome biogenesis.
MamH	10 (9), 6 (4)	9, 3	DNA-binding transcription factor (1, 1); secondary carrier transporter (7, 2); transporter (1, 0).	Possibly involved in redox; affects the crystals’ size, shape, and magnetic properties.
MamK	30 (25), 49 (18)	14, 13	GTPase-activating protein (1); actin and actin related protein (14, 10); transmembrane signal receptor (2)	Magnetosome chain assembly; cytoskeletal filament to position magnetosome organelles.
MamN	2, 3 (2)	2, 2	Primary active transporter (1, 0); secondary carrier transporter (1, 2).	May be involved in iron transport, magnetite nucleation.
Man6	9, 17 (11)	7, 7	DNA metabolism protein (1, 0); chromatin/chromatin-binding or regulatory protein (0, 1); extracellular matrix protein (5, 5); histone modifying enzyme (0, 1); scaffold/adaptor protein (1, 0).	May be involved in chain arrangement or the processes of magnetosome formation.
Mad25/Man6^2^	4, 2	4, 2	Extracellular matrix protein (4, 2).	
MamN/MamE	6, 5 (4)	2, 1	Serine protease (2, 1).	
Totals	244 (226), 332 (181)	136, 101		

Numbers of genes and annotations for the complete repertoire (homologs and paralogs) of zebrafish (*D. rerio*) and Chinook salmon (*O. tshawytscha*) genes encoding distant homologs of 11 MTB biomineralization proteins universally conserved in eukaryotes (uMGHs). Zebrafish (Z) and Chinook salmon (C) genes were matched to ZFIN gene codes for annotation (annot.) and assignment to protein classes in PANTHER. In some cases, multiple fish genes matched to single ZFIN identifiers, as indicated by no. unique. A small number of fish genes matched to both Mad25 and Man6 or MamE and ManN proteins. MTB annotations are summarized from reviews by refs. 65 and 66.

Considering the full repertoire of uMGHs in Chinook salmon, we then examined whether these genes may be engaged with putative magnetite presence in salmonid olfactory cells, and thus biomineralization, in light of the DEG findings at threshold FDR < 0.1. Based on the full repertoire of protein-coding uMGHs in the salmon genome, 12.5 μMGHs are expected to occur by chance in a random sample equally sized to the MAG DEG dataset. We found 18 uMGHs were among the differentially expressed genes, which approaches statistical significance (*P* = 0.0675, one-tailed proportion test, *P* value threshold = 0.05). This indicates that uMGHs may show up-regulated expression in the MAG cell sample. The 18 genes were distributed across 7 of the 11 “universally conserved” categories and included MamABEK, Mad9, Mad25, and Man6 (*SI Appendix*, Table S5 and *SI Appendix*). The differentially expressed MamE homolog shows an exceptional level of conservation to MTB proteins (Fig. 2 *E* and *H*; Dataset S4).

## Discussion

The widespread distribution of magnetite and retention of distant homologs of bacterial magnetite biomineralization genes in eukaryote genomes is interpreted by us as an indication that biologically controlled magnetite precipitation is a fundamental feature of eukaryotic biology and was at one time present in the last common ancestor of extant eukaryotes and some archaea. All but two of the core set of genes we identified as universally present in eukaryotes are detectible in genome contents of Lokiarchaeota, a member of the Asgard superphylum of archaea that forms a monophyletic group with eukaryotes in phylogenomic analyses and whose genome encodes an expanded repertoire of eukaryotic signature proteins (actin and tubulin, which form the core of the cytoskeleton), suggestive of sophisticated membrane remodeling capabilities (29, 30). Our results are thus consistent with eukaryotes having evolved from within the archaea (39–42).

**Fig. 3.**
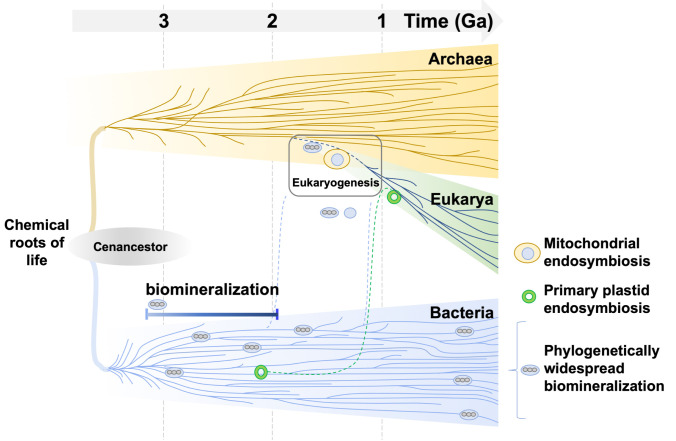
Conceptual schematic of the magnetite evolutionary hypothesis. The timing of ancient serial endosymbiosis events (stylistically adapted from ref. 45) are detailed in refs. 9, 10, and 12 and described in the main text. Uncertainty surrounding timing of eukaryogenesis is depicted by the box.

Could ancient serial endosymbiosis events explain magnetite biomineralization in complex life forms (9, 43) (Fig. 3)? Since the now widely accepted symbiotic origin for some eukaryotic organelles was proposed, a wealth of secondary and even tertiary symbioses events within eukaryotes have been cataloged (reviewed by refs. 44, 45). Here, observed commonality of core biomineralization genes between prokaryotes and eukaryotes is consistent with an ancient endosymbiosis event (9), although an ancient horizontal gene transfer event cannot be ruled out. Regardless of gene acquisition mechanisms, retention of uMGHs in eukaryote genomes (and Lokiarchaeota) signifies that these particular genes are essential features of eukaryotic biology. Our results are parsimonious with the hypothesis that magnetite biomineralization represents deep homology, a latent but plesiomorphic ability (genetic and cellular) to form structures (46), and exaptation of magnetite biomineralization for magnetoreception (7, 43).

The importance of endosymbiosis in the evolution of eukaryotic complexity has become firmly established through accumulation of evidence that mitochondria and plastids (double bilayer membrane-bound organelles) evolved from bacteria (44, 45). A necessary intermediary to endosymbiosis is formation of obligate host–symbiont associations, with numerous examples known to occur at various levels of interdependence and integration, e.g., endosymbiotic bacteria found in cells of insects, nitrogen-fixing spheroid bodies found in some diatoms, and zooxanthellae in marine invertebrates (47). Symbiosis is suspected to occur between members of the Asgard clade of archaea and a candidate division of bacteria (TA06) (40) and was recently documented to occur between MTB and a unicellular eukaryote. In that case, excavate protists (Symbiontida, Euglenozoa) and ectosymbiotic Deltaproteobacteria biomineralizing ferrimagnetic nanoparticles formed a mutualistic relationship based on collective magnetotactic motility with division of labor and interspecies hydrogen-transfer–based syntrophy (48). These assemblages were identified in multiple locations around the northern and southern hemispheres of the globe, and congruence in topology of host–symbiont phylogenetic trees indicates that these partners coevolved and diversified from a single ancestral magnetotactic symbiosis event. Symbiosis between MTB and other forms of life potentially carry a selective advantage, perhaps through a dedicated molecular machinery to sequester excess iron, or perhaps through the physical properties of magnetite, be it a magnetic dipole moments for magnetotaxis of the host [as suggested for a marine protist (48) and for larvae of a marine mollusc (49)], density for adjusting buoyancy in the water column, mechanical stability similar to silica-based phytoliths in grasses and other land plants, hardness for providing protection against grazing, or protection against ultraviolet radiation (50). Consistent with endosymobiosis, mutualistic symbiosis assemblages composed of microbial eukaryotes and bacteria that biomineralize magnetosomes have been observed in multiple locations around the globe (48).

Previous searches for candidate magnetoreceptors in dissociated salmonid ORs using a microscope with an applied rotating magnetic field identified cells with magnetic properties (27). However, in a follow-up study (19), cells isolated in a similar way showed an absence of intracellular magnetite and presence of extracellular contaminants, leading some researchers to question whether olfactory tissues indeed even harbor biogenic magnetite at all (51). Why our search for magnetite was successful in contrast to the cell-spinning approach may be explained by the constraining effect of solution viscosity on spinning properties, with trade-offs between levels of dissociation. Gentle dissociation produces whole cells as necessary for quantifying intracellular components but increases the probability of cells remaining in intact clumps that may not spin, while strong dissociation risks membrane rupture and loss of magnetic contents that are invisible under light microscopy. Alternatively, putative magnetic particle structures from ruptured trigeminal nerve terminals in the OR were released into the cell suspension and adhered to other cells, making them magnetic.

## Conclusions

Our findings are a transformative advance to generate convergent approaches that may illuminate the mysterious “sixth sense” of magnetoreception. Equipped with genomic findings, genetic tools coupled with those of physics, behavior, anatomy, and physiology can be developed to validate associations between candidate magnetoreceptor cells and neural signal transduction. Whether the ancient biomineralization system we nominate here bears a relationship to the numerous other matrix-mediated biomineralization systems found in living organisms (7, 13) or played a role in eukaryogenesis further warrants advancing convergent approaches to resolve the complex innovations that embody life’s diversity.

## Materials and Methods

To minimize contamination by nontarget magnetic particles, the tools used for animal termination and dissection were iron-free and nonmagnetic (made of titanium, ceramic, or glass). All tools and labware used for microscopy protocols, if not presterilized, were cleaned in HCl or ultrasonic bath in EtOH. The tools and labware used for magnetic cell isolation/transcriptomics experiments were cleaned in HCl, with the exception of filter tips used in RNA liquid handling. That work was performed inside a hood equipped with a high-efficiency particulate air filter whenever possible, and tools were covered with plastic wrap as a dust preventative measure. All reagents were ultrapure, molecular biology–grade buffers made with Milli-Q water, and powdered (e.g., papain and L-cysteine [Sigma-Aldrich]) reagents were hydrated in molecular-grade water and filtered through a 0.22-μm membrane using an HCl-cleaned syringe. Fish were obtained from local fish farms/markets or hatchery operations and killed in accordance with European and German regulations or under the authority of permit issued to Oregon State University (ACUP 4421).

### Salmonid Candidate Magnetoreceptors.

#### In situ magnetic measurements.

To assess in situ magnetic properties, the olfactory epithelium of rainbow trout (*O. mykiss*, *n* = 10) was isolated bilaterally and frozen for measurement of FMR absorption spectra acquired using an X-band ESR spectrometer (JEOL, JES-FA 200), at a microwave frequency of 9.07 GHz, 4-mW input power, and a magnetic field sweep rate of 200 mT/min. For lock-in detection, the applied magnetic field was modulated with a 0.4-mT magnetic field of 100 kHz frequency. Findings were compared to experimentally observed FMR spectra of MTB quantitatively explained using the theoretical model developed in Charilaou et al. (52).

#### Microscopies.

##### Scanning probe microscopy.

To study biogenic magnetism at the nanoscale, the physical and magnetic features of salmonid and bacteria magnetite particles were determined using a custom-designed scanning probe microscope with AFM and MFM modes. Biogenic magnetite particles were extracted from Atlantic salmon olfactory epithelium and the MTB *Magnetospirillum gryphiswaldense* MSR-1 and compared to a commercial ferrofluid (sample preparation details available from *SI Appendix*, *Extended Methods*). The scanning probe microscope (Veeco Digital Instruments) was equipped with a small, super-sharp AFM/MFM tip attached to a commercially produced cantilever (53). The tip, with a curvature radius less than 10 nm, was made from a microfabricated silicon probe selectively coated with 30 nm Co_85_Cr_15_. The tip and cantilever had a resonant frequency of 75 kHz and a spring constant of 3 N/m for measurement of topography and magnetic signals using MFM tapping and lift modes, setting the lift height in MFM measurements to 20 nm. External in-plane magnetic fields were generated by a pair of solenoids. The field strength was enhanced by a pair of iron cores, with a maximum field in the middle of two iron cores measured as 370 mT. The sweeping function of the magnetic field within the MFM was realized by the combination of a function generator (HP 33120A) and a self-made voltage to current converter with a maximum current of 8 A for the employed solenoid. The salmon magnetite sample was visualized in external fields applied normal (*z* axis) to the sample surface at field strengths of 0.5, 3.5, 7, 15, and 35 mT. The externally applied magnetic fields orients all particle magnetic moments partially or even completely along the field direction. Since the probe magnetization is also partially aligned, attractive magnetostatic interactions between probe and magnetic nanoparticles result. These interactions are specifically measured upon lifting the probe in the MFM mode of operation and they manifest themselves in terms of a dark contrast. In the tapping mode of operation, the oscillating probe is periodically almost in contact with the sample, which results in a topographical image irrespectively of the nanoparticle magnetic configurations. Additional microscope details are available from (53).

##### Confocal microscopy.

Rainbow trout olfactory epithelium and MTB were examined using reflectance mode of the confocal microscope, based on previously developed protocols (5, 54) and described in *SI Appendix*, *Extended Methods*. A sample of competent *Escherichia coli* (DH-5) was used as a nonmagnetic control. The MTB were obtained from mud samples collected in the Rhin Tortu, Strasbourg, France (48°32′59.1″N, 7°45′38.0″E). Samples were imaged using a Leica TCS SP5 II Laser Scanning Confocal microscope with a 63× oil immersion objective (numerical aperture 1.40). fn1-43fx was excited at 488 nm and emitted light collected using a 500 long pass filter. DAPI was excited at 405 nm. The reflectance mode option of the confocal microscope was calibrated using the MTB reflectance. Further analysis and image presentation were performed using ImageJ software (55). Confocal microscopy was performed at the in vitro imaging core facility (CNRS UPS3156) located at the Institute of Cellular and Integrative Neuroscience, Centre National de la Recherche Scientifique, Strasbourg, France.

#### Salmonid transcriptomics.

##### Biological sampling.

Tissues for RNA isolation were sourced from Chinook salmon reared in a single tank at the Fish Research Laboratory, Corvallis, OR (44°33′52.4″N, 23°15′43.4″W). 15 fish were sampled for OR tissues used in magnetic cell isolation experiments, one fish was sampled for OR deep transcriptome sequencing, and three fish were each sampled for muscle tissue, blood, and additional pairs of ORs. Muscle tissue was used as a negative control to rule out potential presence of contaminants during magnetic cell isolation experiments. For olfactory MAG and NM transcriptome profiling, three replicate experiments were conducted by enzymatically dissociating olfactory tissues, then isolating MAG cells by conducting magnetic collection (using a fine-point magnet placed on the exterior of a glass vial), during which NM cells collected on the bottom of the vial through gravitational forces (*SI Appendix*, *Extended Methods*). Given the scarcity of MAG cells in olfactory tissues, three to five sets of ORs were combined in each experiment to obtain sufficient material for visualization of the magnetic pellet under a dissecting microscope. The magnetic cell pellet was aspirated and placed in a RNase-free vial with ∼20 μL buffer, followed by transfer of an aliquot (∼20 μL) of the nonmagnetic cells to a separate RNase-free vial. All other transcriptome samples—*n* = 3 muscle, *n* = 3 blood, and *n* = 4 pairs of ORs—were individually processed. The fish fork lengths ranged from 10 to 15 cm.

#### RNA extraction and Illumina sequencing.

In the presence of QIAzol Lysis Reagent (Qiagen), solid tissues (untreated ORs and muscle) were mechanically homogenized and lysed with an electronic mortar and pestle, while blood and dissociated MAG and NM OR cells were homogenized and lysed by pipetting. Total RNA was isolated from lysed materials using a Qiagen RNEasy Mini kit following manufacturer’s protocols. Samples were submitted to Oregon State University’s Center for Genome Research and Biocomputing core facility for messenger RNA isolation, Illumina library preparation, individual indexing for demultiplexing, and sequencing on an Illumina HiSeq2000. Each experimental pair of MAG and NM samples was sequenced in a single Illumina lane using 101 cycles and paired-end protocols, with one lane also including the additional snap-frozen single OR sample for deep sequencing. The other nine samples—blood, muscle, and OR tissues—were single-end sequenced in a single lane using 50 cycles.

#### RNA sequencing data processing and mapping.

The raw Illumina reads were quality processed with Trimmomatic (56) (version 0.32), removing adapter contaminants and low-quality sequences and retaining reads ≥25 nucleotides in length with an average sequencing quality of phred 20 across 4 nucleotide sliding windows. Reads were mapped with Bowtie2 version 2.2.1 (57) (setting: very sensitive) to a Chinook salmon reference transcriptome based on a Chinook salmon genome (36) having a total sequence length of 2.54 Gb (National Center for Bioinformatic Information Accession GCF_002872995.1). This genome’s companion *rna.fna file contains 81,329 predicted RNA transcripts that correspond to 73,277 predicted proteins and their variants. The longest RNA transcript per gene (*n* = 47,921 transcripts) was selected for inclusion in the reference transcriptome used for read-mapping, differential gene expression analysis, and bidirectional BLASTp comparison to MTB biomineralization proteins (MTB accessions available from Dataset S3).

##### Differential gene expression analysis.

Differential gene expression was modeled using a generalized linear model likelihood ratio test implemented in EdgeR (58). With focus on MAG samples, pairwise contrasts were made to NM experimental counterparts, blood, and muscle tissues. Magnetoreceptors are presumed to be absent from the latter two sample types, and their expression profiles may be useful for making general inferences about gene functions. Data inputs for EdgeR included counts of mapped forward reads (to match single-end sequenced samples) extracted from *bam files. Transcripts were filtered for low expression using a minimum of two count-per-million reads across at least three of the 16 samples, adjusting for high expressed reads using trimmed mean of M component read normalization (59). Postfilter, per-sample mapped read numbers ranged from 8.6 to 46.0 million (average 21.7 million; SD 9.0 million). A total of 38,598 (81% of 47,921) RNA transcripts were considered in differential gene expression analysis. Statistical significance was adjusted for multiple tests using Benjamini–Hochberg (B-H) (60) FDR-corrected *P* values with a threshold cutoff of FDR < 0.05 for broad contrasts between MAG and all tissue types and FDR < 0.1 for analysis of genes differentially expressed in the MAG–NM contrast. Broad relationships among gene expression profiles were visualized and inspected through multidimensional scaling plots (EdgeR function plotMDS) and heatmaps (gplot version 3.0.1 function heatmap.2) generated in EdgeR version 3.12.1 with R version 3.2 (61).

##### Functional annotations.

The molecular functions of zebrafish and Chinook salmon genes encoding uMGHs and genes differentially expressed in the MAG cell sample (contrasted to NM cell sample, FDR < 0.1) were annotated using the Protein ANalysis THrough Evolutionary Relationships classification system (37) (PANTHER version 13.1, release date February 3, 2018). To leverage well-characterized gene ontology terms from a model fish species, the Chinook salmon mRNA transcripts were BLASTx matched to zebrafish (*D. rerio*) orthologs (ENSEMBL genome version GRCz11, file “Danio_rerio.GRCz11.pep.all.fa”; last modified March 8, 2018) to identify PANTHER-compatible ZFIN identifiers (35). Nonspecific BLASTx matches were filtered by applying a threshold cutoff *E* < 1e−5. Of the 1,588 MAG DEGs, 1,333 zebrafish ZDB gene identifiers were procured. Statistical tests for overrepresentation across GO complete categories (Overrepresentation Release 20181113; GO database release January 1, 2019) and reactome pathways (Reactome version 65, released June 12, 2018) were assessed on the basis of fold-enrichment values, dividing the observed by expected numbers of per GO or pathway term. This denominator is based on the zebrafish background genome and considers the number of genes in the input file. The zebrafish genome was used as a background genome. Statistical significance was adjusted for multiple tests using PANTHER’s built-in B-H FDR correction function. Individual zebrafish and Chinook salmon genes encoding uMGHs (see below) were also categorized by PANTHER family/subfamily groups and protein classes, based on ZFIN identifiers (35), using the 2020_04 release of the ReferenceProteome dataset. Protein names of individual DEGs were also obtained from the Chinoook salmon RefSeq genome feature table (GCF_002872995.1).

Macrophages, a type of immune system cell that can precipitate and store iron deposits (62), were evaluated as a potential explanation for the observed magnetic properties of dissociated cells used for transcriptome experiments. Genes annotated as “macrophage” (*n* = 261 ZDB genes) in the ZFIN data repository (35) were matched to annotations for NM and MAG DEGs (at FDR < 0.05) and evaluated for statistical overrepresentation using a one-sided proportion test with a threshold significance value of *P* = 0.05.

### “Universal” MGHs.

Whether distant homologs of MTB biomineralization proteins are universally present among eukaryote genomes was assessed by comparing genome contents of 13 phylogenetically diverse eukaryote taxa (*SI Appendix*, Table S2) to a database of magnetosome proteins of distantly related phyla including Nitrospirae and Proteobacteria (classes Alpha, Delta, and Gamma; Uniprot-KB SWISS-PROT database download date 9/12/2018; search term name = “magnetosome”) (accessions provided in Dataset S3). An MGH was classified as “universal” (uMGH) in eukaryote genomes if a bidirectional BLASTp match to a named MGH occurred across at least 12 of the 13 eukaryote genomes (>92%), allowing for one missed protein product annotation or gene loss. Nonspecific matches were filtered by applying a threshold cutoff Expect value (*E*) of *E* < 1e−3, considered reliable for inferring gene descendants with distant homology (63), in the eukaryote/archaea to bacteria comparison. The MTB protein database contained 106 named magnetosome genes (similarly named genes were kept separate, e.g., MamK and its paralog MamK2; ref. 64) represented by 594 sequences meeting a threshold minimum length of 100 amino acids (Dataset S3). Genes labeled as “Unknown” (*n* = 7) were excluded from consideration as uMGHs. To account for evolutionary distance, the Lokiarchaeota uMGH assessment included matches to uMGHs and similarly named MTB homologs.

#### Fish uMGH repertoire identification.

The full repertoire of genes encoding uMGHs in zebrafish and Chinook salmon was identified through unidirectional BLASTp queries of fish genome contents to the magnetosome protein database, applying a threshold *E* < 1e−3 filter to remove nonspecific matches (63). As the objective here was to identify the complete repertoire of genes with distant homology to MTB magnetite biomineralization genes, we retained both matches to named uMGHs and matches to their homologs and grouped them under a single gene identifier (i.e., MamK and MamK2 were retained and grouped as “MamK”). Based on the full uMGH repertoire of Chinook salmon, whether the relative frequency of DEGs characterized as uMGHs was greater than expected was tested using a one-tailed proportion test (without Yates continuity correction). The background global frequency of uMGHs was calculated by dividing the number of protein-coding uMGHs (*n* = 367) by the genome-wide number of protein-coding genes (*n* = 42,215). Only DEGs characterized as protein coding were considered in this analysis (*n* = 1,433 of 1,588 DEGs). Calculations were made in R package *stats* version 3.2.1 (61), with statistical significance set to *P* < 0.05.

## Supplementary Material

Supplementary File

Supplementary File

Supplementary File

Supplementary File

Supplementary File

Supplementary File

Supplementary File

## Data Availability

The RNA sequencing data used for differential gene expression modeling are available through National Center for Biotechnology Information BioProject accession no. PRJNA614978. All other data are available from *SI Appendix*, Datasets S1–S6, and public repositories as described within the text.
