# Supplementary material for: Conservation of magnetite biomineralization genes in all domains of life and implications for magnetic sensing
Source: Proc Natl Acad Sci U S A. 2022 Jan 10;119(3):e2108655119. doi: 10.1073/pnas.2108655119 (PMC8784154; doi:10.1073/pnas.2108655119)
Supplement: Supplementary File [file pnas.2108655119.sapp.pdf]

PNAS
www.pnas.org

Supplementary Information for
Conservation of magnetite biomineralization genes in all domains of life and implications for magnetic sensing

M. Renee Bellinger*[1†], Jiandong Wei[2††], Uwe Hartmann[2], Hervé Cadiou[3], Michael Winklhofer[4,5], Michael A. Banks[1]

Corresponding Author: M. Renee Bellinger
Email: belling@hawaii.edu

This PDF file includes:

Supplementary text
Figures S1 to S5
Tables S1 to S6
SI References

Other supplementary materials for this manuscript include the following:

Datasets S1 to S6

Supplementary Information Text

This Supplementary Information text provides additional context for microscopy results, evidence against contaminants in experiments, detailed annotations for differentially expressed genes matching to distant homologs of ‘universally conserved’ magnetosome-associated genes (uMGHs) of magnetotactic bacteria, discusses the magnetite evolutionary genetics hypothesis in further detail, and contains Extended Methods.

1) Salmonid Candidate Magnetoreceptors

Visualized with a combination of ferromagnetic resonance spectrum (FMR) and atomic and magnetic force microscopies (AFM/MFM), we found that magnetically extracted particles of Atlantic salmon (*Salmo salar*) occurred in the form of large aggregates (Fig. 1C-G, Fig. S2). A closer examination of the aggregates reveals a conspicuous substructure (Fig. 1E), consisting of 300 nm wide, tightly packed agglomerations of dozens of sub-100 nm magnetic particles (Fig. 1C, D). Originally, we expected to obtain chains of magnetic particles similar to those reported by Mann et. al. (1), who magnetically isolated particles from homogenized ethmoid tissue of the sockeye salmon, *Oncorhynchus nerka*, albeit with a more aggressive digestion protocol, using commercial bleach. While the *in-situ* arrangement of magnetic particles cannot possibly be inferred from digested tissue homogenates, it can at least be constrained from ferromagnetic resonance (FMR) measurements of intact tissue, because linear chains of magnetic particles would have FMR signatures that are distinctly different from three-dimensional magnetic particle agglomerates (2–5). Significantly, our FMR spectra measured on 20 olfactory rosettes from 10 juvenile rainbow trout are inconsistent with those to be expected for a population of single-strand chains of magnetic particles (Fig. 1B, Fig. S1). Notably, FMR absorption at magnetic fields well below 100 mT points to the presence of magnetically soft components, which is in agreement with our MFM observations that revealed switching fields as low as a few mT (Fig. S2). Thus, from the FMR spectra, we can infer the presence of more complex agglomerates of particles, which tallies with the atomic-force microscopy maps of tissue sections presented in Diebel et. al. (6), showing a cluster structure of particles in a section of a candidate magnetoreceptor structure in the olfactory epithelium of rainbow trout. Similarly, confocal reflectance images of putative magnetoreceptor structure presented previously (7) and

here (Fig. S5) reveal reflective particle structures which are broader compared to magnetosome chains in positive controls. From these observations, we surmise that the inner magnetic architecture of the putative magnetoreceptor is more complex than just a chain.

Confirming magnetite's involvement in signal transduction is currently hindered by a lack of obvious physical characteristics visible through light microscopy, and no methodology is yet available to locate physiologically responsive cells for assessment of magnetic sensitivity. However, as exemplified in Fig. S5, reflectance mode of confocal microscopy can be used to identify candidate cells *in situ*, which holds promise for testing gene-specific hypotheses regarding their involvement in magnetite crystal production. We demonstrate the utility of this technique using reflectance mode on a magnetotactic bacteria sample, which revealed elongated objects between 0.5 and 1 um in length, enclosed by bacterial membranes labelled with the lipophilic dye FM1-43fx. On close examination, no reflectance signal was ever seen outside the bacteria delimited by the FM staining of the bacterial membrane, indicated on by the red arrow (Fig. S5A, far right panel). Observing an *Escherichia. coli* sample using the same conditions resulted in an absence of reflective signal (Fig. S5B) in contrast to magnetotactic bacteria and trout olfactory epithelium (Fig. S5C). Visualization of the trout olfactory under the reflectance mode allowed us to observe a single reflective particle (approx. 1 μm in length). These are rare objects (one per section). Developing novel single cell genetic and fluorescence tools and improved microscopy methods for ultra-structure analysis will be major steps towards unlocking the mystery of how animals are able to sense geomagnetic fields, and to identify key components of the genetic, physiological, and structural basis of magnetite-containing cells of vertebrates.

We can rule out contaminants as an explanation for presence of magnetite in salmon epithelial tissue using AFM/MFM microscopy by comparing findings to those of bacteria and commercial ferrofluid magnetite particles. As shown in Fig 1D, the narrow size distributions of salmon magnetite particles indicates their formation is under strict genetic control, in contrast to external contaminants that are expected to show irregular shapes and sizes (1, 8–10). Self-assembled clusters are highly unlikely to occur as a result of synthetic or bacteria magnetite particle uptake because there is no kinetic or

thermodynamic reason for the formation of well-defined, regularly sized, ellipsoid shaped clusters (Fig. 1C-G). These particle clusters, along with magnetic properties of crystals, are increasingly apparent as visualized using AFM/MFM while applying a range of external magnetic field strengths (Fig. S2). No such self-assembly of particles was observed for the ferrofluid magnetite, which formed 'islands' of varying sizes contingent on external magnetic field strength (Fig. S3A-C), or bacteria particles, which formed long chains (Fig. S3D, E; close-up of particles shown in F). In the absence of external magnetic fields during drying the ferrofluid magnetite formed small particle islands, but when subjected to a 20 mT in-plane static magnetic field the particle islands increased in size, reaching lengths of about 10 µm, leaving a major part of the substrate bare (Fig. S3B). Visualized under AFM, those particles were arranged in mono- or bilayers. The island size further extends by applying higher magnetic fields, for example, the in-plane field of 40 mT (Fig. S3C). Collectively, the salmon magnetite particles and their arrangement are distinct from those of MTB and commercial magnetite preparations.

For transcriptomics experiments, we found no evidence for macrophages explaining the magnetic properties of dissociated olfactory cells. The NM samples contained 5.5x greater proportion of expressed macrophage genes compared to the MAG samples (1.1% vs. 0.2% of all differentially expressed genes with ZFIN annotations: NM, 29 macrophage genes / 2547 ZFIN identifiers; MAG, 1 macrophage gene / 455 ZFIN identifiers). The fact that macrophage genes were significantly and more highly expressed in the NM sample than in the MAG sample ($p = 3.45e-11$, one-sided proportion test), indicates that macrophages are not responsible for the magnetic properties of olfactory cells used in the transcriptome experiment.

2) 'Universal' Magnetosome Gene Homologs

The genome contents of 13 eukaryote taxa were compared to a database of 594 magnetotactic bacteria (MTB) biomineralization proteins (plus seven proteins listed as "Unknown") grouped into 105 named protein categories, based on UniProtKB naming conventions (Datasets S2, S3). This grouping simplified the evaluation of presence/absence patterns of MTB genes in eukaryotes, but also masks interesting cases whereby similarly named MTB protein sequences show a high prevalence of bidirectional match. Considering individual proteins, the majority of MTB proteins had bidirectional matches across 3 or fewer

eukaryote genomes (n=532, of which 401 had zero match, Dataset 2). Yet, fourteen individual MTB protein sequences displayed bidirectional match across 7 or more of the 13 eukaryote genomes considered (summarized in Table S3; Dataset S2). In the context of using protein names as grouping criteria, three of those 14 proteins, Mad27-1, Mad27-2, and one Unknown, were excluded from the uMGH category designation because of more than one gene loss (our criteria for 'universal' presence) across the 13 eukaryote genomes (Dataset S3). Had we used a less conservative approach, i.e., grouping homologs of homologs within MTB, the Mad27 gene would have been included within the uMGH category. Accordingly, we do not intend the candidate gene list presented in this study to be construed as an exhaustive list, rather, these genes are anticipated to represent only a fraction of those involved in iron mineral biomineralization by higher organisms. In sum, the uMGH category can be considered conservative, and gene match data can be further mined to fine-tune magnetite biomineralization models.

If ancient bacterial genes were inherited as functional units they would be expected to cluster on chromosomes. To test for disproportionate representation of uMGHs on salmon chromosomes, the per-chromosome number of uMGH was compared to the number of expected uMGHs, applying a one-tailed proportion test with Yates continuity correction to account for low numbers (<5) on some chromosomes. Statistical significance was set to $p < 0.05$ for this test. The full Chinook salmon uMGH repertoire indicates distant homologs of magnetite-associated proteins are significantly enriched on Chromosome 6 ($p = 0.003$), and approaches statistical significance on Chromosomes 1 and 24 ($p = 0.066$; $p = 0.064$) (Table S6). The MamAKEB gene combination occurs on seven of 35 salmon chromosomes, including chromosomes 1 and 5 (sharing a >2 million bp homeologous block (36)), where they co-occur with Mad25 and Man6.

Differentially Expressed Candidate Genes

The Chinook salmon olfactory epithelium genes we identified as universal magnetosome gene homologs (uMGHs) and differentially and more highly expressed in the magnetic samples (contrasted to non-magnetic counterparts) show a range of conservation and similarity to MTB magnetosome-associated proteins. Among those 18 differentially expressed uMGHs, all but one protein sequence shares a

homology class with its corresponding MTB biomineralization protein match (Table S5). Based on BLASTp results, these Chinook salmon protein sequences are on average 26% similar (median 29%) across an average length of 154 amino acids (median 150) to their MTB subject sequence match, with the MamE homolog showing an unusually high level of conservation, 35% match across 212 amino acids. The reciprocal BLASTp match for that gene was to MTB MamEO-Nter, but also matched equally well to other MamE MTB sequences contained in the MTB protein database. Although the MamE amino acid sequences show extreme conservation across MTB and magnetically sensitive eukaryotes (Fig. 2, full protein alignment in Dataset S4), that gene is absent from the Asgard archaea Lokiarcheota genome assembly.

Eight of the 18 differentially expressed uMGHs match to MamA, characterized by tetratricopeptide motifs (TPR), a structural motif that mediates protein-protein interactions. Although this motif is present in numerous unrelated proteins and occurs ubiquitously in Bacteria and eukaryotes (11), 100% of the eukaryote genomes and one differentially expressed gene (DEG) had a bidirectional match to a specific Nitrospirae MamA gene (UniProtKB accession A0A142BTV4, Dataset S3, Tables S3, S5). One DEG with distant homology to MamB belongs to the CDF superfamily of proteins involved in heavy metal transport that occurs ubiquitously in Eukarya, Bacteria, and Archaea (12). The second distant homolog to MamB was the only DEG failing to show a conserved sequence domain classification that matched the MTB, despite BLASTp characteristics being on par with average values noted above. The MamK MTB protein is a homolog of the bacterial actin-like protein MreB, which appears to be used as a cytoskeletal filament to position individual magnetosome organelles within the cells (13). The DEG showing distant homology to MamK is an ARP3 actin related protein 3 homolog, which has diverse functions, including playing a major role in the regulation of the actin cytoskeleton (14, 15). This DEG also matched to the same MTB protein that received the greatest number of bidirectional BLASTp matches (9/13 eukaryote genomes) across all MamK genes (A0A0M9E4Z8) in the MTB magnetosome biomineralization protein database (Table S3, Dataset S3). Also relevant to actin, the Mad25 homolog, *Rho-associated protein kinase 1-like isoform*, is a key regulator of actin cytoskeleton and cell polarity. As with MamK-2, the bidirectional match counts across eukaryotes was greatest for this particular MTB amino acid sequence (9/13 eukaryote genomes). The

second DEG with homology to Mad25, *trafficking kinesin-binding protein 1-like*, is known for involvement in vesicle transport along a microtubule and stimulating axonogenesis. These Mad25 genes are characterized as belonging to superfamily SMC (structural maintenance of chromosomes), same as Man6 in both MTB and eukaryote uMGHs. The Man6 protein binds DNA and acts in organizing and segregating chromosomes for partition and is found in Bacteria, Archaea, and Eukarya. Last, the Mad9 uMGH is predicted to have several functions, including iron ion binding, and is one of only 16 genes in zebrafish belonging to the PANTHER category "regulation of biomineral tissue development”.

Although not all essential features of MTB proteins are known, at least one of the uMGH proteins we identified is missing a functional element. In this case, the highly conserved MamE protein, a HtrA-like serine protease, is missing the magnetochrome domain (16) (double-heme binding site, motif CxxCH), despite the striking amino acid conservation noted in Fig. 2 and Dataset S4. This domain is also missing from the MTB Deltaproteobacteria MamE homolog, which is split into two proteins, MamE-Nter and MamC-ter, in that group. Numerous iron-binding proteins are present within eukaryotes (17), and this motif is common in their genomes, for example occurring in 144 Chinook salmon proteins, thus functional compensation through interactions with other proteins is certainly possible. In general, reciprocal blast matches between MamE and MamEO were similarly scored, which indicates those proteins share conserved features across the entire length of the protein sequence.

3) Magnetite Evolutionary Genetics Hypothesis

Regarding timing of the acquisition of magnetite biomineralization by eukaryotes, we can (mostly) rule out the scenario that the Alphaproteobacteria endosymbiont that resulted in mitochondria was a magnetotactic bacterium, because archaea lack mitochondria. However, we cannot rule out serial endosymbiosis, or an ancient horizontal gene transfer event having occurred while the bacteria/archaea lineage was evolving or after their split (Fig 3). In either case, our findings indicate the most likely scenario would have involved the Nitrospirae lineage, based on their genome contents containing the Mam/Mad/Man gene combination (18, 19) and patterns of matches to distant homologs that we identified in eukaryotes and Lokiarchaeota (Dataset S3).

The biogenic production of magnetite in eukaryotes was first documented to occur in chiton (Mollusca, Polyplacophora), where crystals of magnetite cap radula teeth to reinforce the cutting edge. Chiton have long served as a model system for extracellular magnetite biomineralization processes, with the radula tooth consisting of a chitin-based organic fiber matrix with fibrous bridges. In the chiton model, magnetite most likely forms from a ferrihydrite-like hydrous iron oxide phase transported within a ferritin host protein, which fuses with the cell membrane and releases the mineral core to the extracellular matrix where it reorders to form magnetite. Rapid advances in next-generation sequencing hold promise for discovering the suite of genes involved in magnetite biomineralization pathways, however that research is in early stages. An *Acanthopleura hirtosa* genome has been sequenced but is not yet a publicly available resource. Two of the most primitive molluscan groups, the Archaeogastropods and the Polyplacophorans, both use iron minerals to harden radular teeth (goethite and magnetite, respectively), indicating that biological process has ancient origins. Whether the genetic machinery that underpins extracellular magnetite biomineralization in chiton shares commonalities with intracellular magnetite formation in eukaryotes, and magnetite-producing bacteria, remains an open question.

Extended Methods

Microscopies

Scanning Probe Microscopy

<u>Magnetite particle extraction and preparation</u>

Atlantic salmon (*Salmo salar*) olfactory epithelium was dissected from adult fish heads and then dissolved overnight at 60°C in a lysis buffer containing 5 ml of 400 mM Tris HCl (hydroxymethyl aminomethane hydrochloride, pH 8.5), 0.5 ml of 0.5 M EDTA (ethylene diamine tetraacetic acid), 1 ml of 10% SDS (sodium dodecyl sulfate), 2 ml of 5 M NaCl, 0.25 ml of 20 mg/ml proteinase K and 41.25 ml of H_2O per 50 ml solution. The solution was centrifuged at 1.7×10^4 rpm, and buffer replaced with 10 M NaOH for resuspension of particles and a second overnight incubation at 60 °C. Finally, the solution was centrifuged at 1.2×10^4 rpm and NaOH replaced with water for particle resuspension and overnight

collection with a magnet. The magnetic particles were transferred to a freshly cleaved mica surface by a pipette.

The magnetotactic bacteria *Magnetospirillum gryphiswaldense* MSR-1 were cultured microaerobically (0.125 mbar of oxygen, stationary condition) in a modified Biostat A twin dual vessel laboratory fermenter (B. Braun Biotech International GmbH) with a 100 ml culture volume set to 28 °C under moderate shaking (100 rpm) for 24 h in modified flask standard medium (51, 52). Bacteria (2.55×10^9 cells/ml) membranes were disrupted by 3 passages through a French pressure cell (53) at 1260 bar, after which unbroken bacteria and cellular debris were removed by centrifugation at 800 g for 5 min. The cleaned cell lysate was passed through a magnetic separation column (Miltenyi Biotech GmbH), and column-bound magnetite particles serially washed by 50 ml of EP (10 mM Hepes, 1 mM EDTA pH 7.4), HP (10 mM Hepes, 200 mM NaCl, 1 mM EDTA pH7.4), and water. Next, particles were eluted from the column, centrifuged at $2x10^5$ g for 90 min, resuspended in 2 mL EP, and then transferred to a freshly cleaved mica surface by a pipette.

The commercial ferrofluid EMG 805 Ferrotec contains 3.6% (in volume) spherical magnetite nanoparticles approximately 10 nm in diameter. This magnetic fluid was diluted to a volume ratio of 1:1000, and droplets from the solution transferred to a freshly cleaved mica surfaces for drying.

The biogenic magnetite sample preparations (salmon and bacteria) were dried in air in an external homogeneous magnetic field of ~45 mT, produced by an electromagnet. The ferrofluid samples were dried in the absence of external magnetic fields, and in 20 mT and 40 mT in-plane fields. After drying, to avoid possible electrostatic charging, biogenic magnetite samples were covered with a conductive coating of gold by means of sputtering.

Confocal Microscopy

Sample preparation

Magnetically collected MTB were placed on a HCl-cleaned coverslip, dried, then quickly rehydrated using PBS and incubated with FM 1-43fx (ThermoFisherScientific) for 5 minutes at room temperature, with FM 1-43fx denoting the fixable analogon of the lipophilic dye FM 1-43 (55). Bacteria were then fixed with PFA 4% IN PBS and 0.2% Glutaraldehyde. Following that, coverslips were mounted on a glass slide using MOWIOL 4-88.

To prepare biological samples for confocal microscopy, juvenile Rainbow Trout were decapitated and heads were fixed in 4% PFA in PBS. Next, the trout heads were rinsed thoroughly with PBS, cryoprotected in 30% sucrose in PBS overnight, then frozen in liquid Nitrogen after embedding in OCT. Cryosections, 20 microns thick, were generated using a CM 3050s Cryostat (Leica) equipped with a ceramic blade (a modified ceramic Nakiri knife, Kyocera, Japan) and deposited on HCl-treated glass slides. Slices were then bleached using a solution of 1.2% Hydrogen peroxide, 10% Formamide in PBS, and exposed to direct light in order to bleach melanin granules which could possibly reflect laser light.

Salmonid Transcriptomics

Biological Sampling

Sample preparation and magnetic isolation protocol

For each of the three replicate magnetic cell isolation experiments the ORs were rinsed in molecular-grade water, diced into small pieces, and placed in individual vials containing 350 uL buffer solution (200 mM sucrose, 20 mM Hepes, 4 mM EDTA, pH to 7.4), with five uL 2-mercaptoethanol to aid RNA preservation. To limit bacterial growth the vials used for two replicate trials were treated with 150 ul of antibiotic/antimycotic buffer (Invitrogen) diluted to 1x in Hanks Balanced Salt Solution pH 7. Vials were held at -20˚ C until cell dissociation, typically occurring within 2 hours of sampling. The additional pair of ORs, taken during the third trial and collected for deep transcriptome sequencing, were rinsed, placed in buffer, and snap-frozen in a slurry of dry ice and ethanol followed by transfer to a -80˚ C freezer for

storage. The three sets of muscle, blood, and OR samples were processed the same way as the deep-sequenced OR sample.

Magnetic (MAG) olfactory cells were separated from non-magnetic (NM) olfactory cells by magnetic collection of enzymatically dissociated cells. Individual pairs of diced OR tissues were dissociated in buffer solution by adding 100 uL of 2.5 mg/mL papain activated by five mM L-cysteine and 20-40 uL of Trypsin-EDTA solution 0.25%, keeping vials at ~12˚ to 15˚ C for up to two hours. Contents of three to five vials (depending on OR sizes) were gently triturated and transferred to a 50 mL beaker where solution viscosity was reduced by gently spinning cells in the presence of 1% triton-X at 4˚ C for 30 minutes. Next, ~3 mL of the cell suspension was transferred to a 4 mL glass vial (length 7.5 cm), contents mixed by gentle inversion, and the vial was stationed in a wooden stand positioned with a neodymium magnet affixed with a pointed magnetized tip (a 1/4 inch sawed off tip section from a 2" nail) placed mid-point on the vial's upper side. Overnight, magnetic cells collected at the magnet's pointed tip while opposing gravitational forces pulled non-magnetic cells to the vial's bottom. Plastic wrap secured over the top of the vial reduced contact with dust. The following morning the magnetic cell pellet, viewed under a dissecting microscope, was aspirated and placed in a RNAse free vial with ~20 uL buffer, followed by transfer of an aliquot (~20 ul) of the non-magnetic cells to a separate RNAse free vial. Vials were stored at -80 C until RNA extraction.

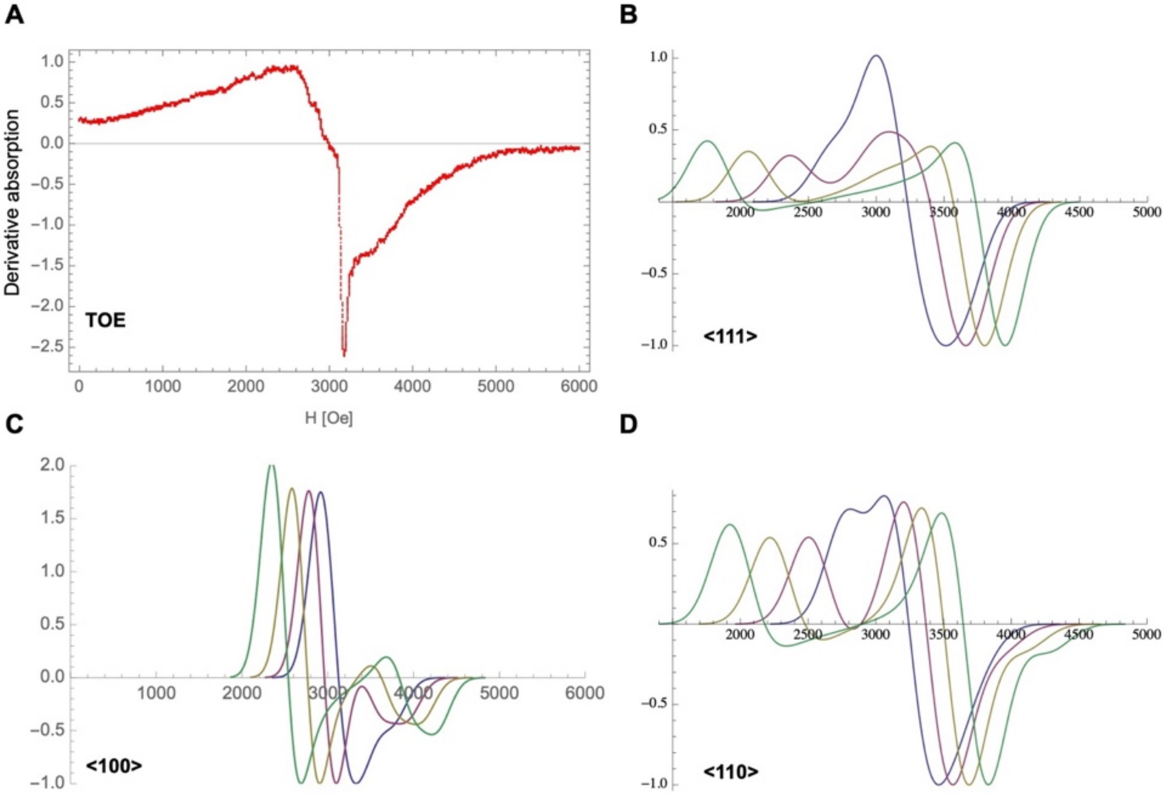

Fig S1. Ferromagnetic resonance spectra (FMR). Experimental FMR spectrum of rainbow trout (*Oncorhynchus mykiss*) olfactory epithelium (***A***, TOE) in comparison with model FMR spectra of magnetosome chains calculated with a theoretical model by Charilaou (4) which was able to quantitatively explain experimentally observed FMR spectra of magnetotactic bacteria (***B-D***). Since the FMR spectrum of a magnetosome chain depends on the crystallographic orientation of magnetosome crystals with respect to the chain axis, three different crystallographic orientations are juxtaposed here (<111>, <100>, and <110>, where <111> is typical for MTB from the Alphaproteobacteria and <100> (which includes which includes the [100], [010], [001] directions) is common among Deltaproteobacteria subphyla). The spectra are color-coded according to the shape anisotropy of individual crystals, ΔN Ms, where ΔN is the difference in demagnetization between short and long particle axis, and Ms is the saturation magnetization (blue – ΔN =0.05 , magenta – ΔN=0.1, brown – ΔN=0.15, green – ΔN=0.2, see (4) for details of the model. In essence, the larger the aspect ratio of a single magnetosome crystal, the broader the magnetic field span over which FMR absorption occurs (1000 Oe corresponds to 100 mT). Note that the FMR absorption spectrum of TOE extends to zero field, in stark contrast to the spectra of magnetosome chains.

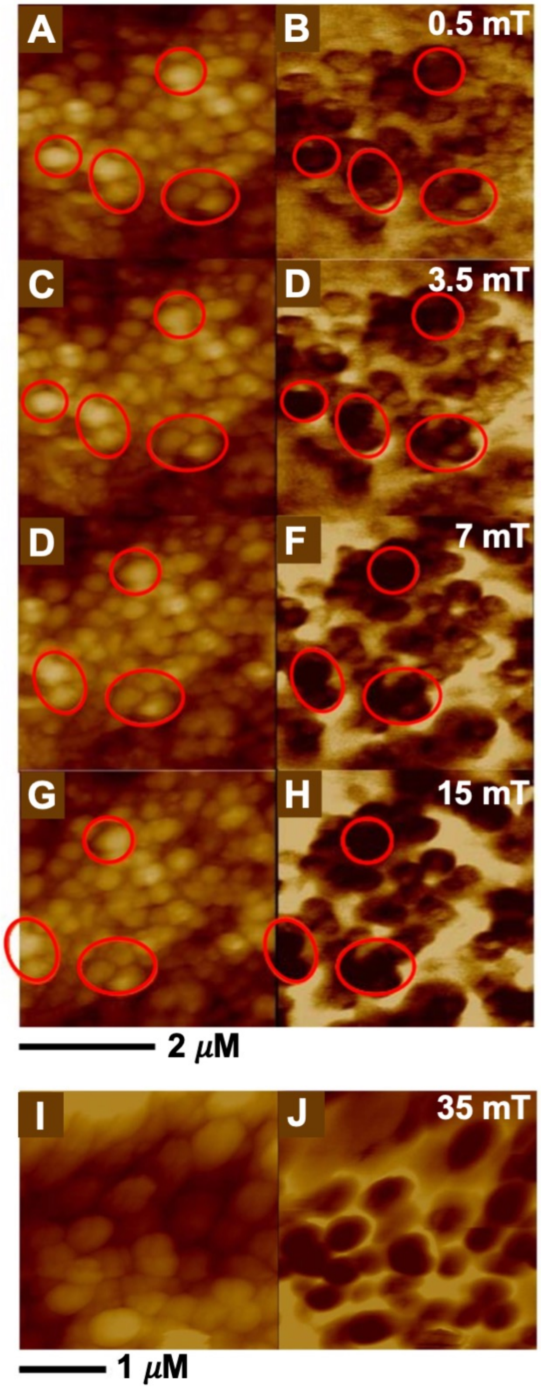

Fig. S2. Microscopy images. Atomic and Magnetic Force Microscopy images of magnetite clusters extracted from Atlantic salmon (*Salmo salar*) olfactory epithelium. (***A***), (***C***), (***E***), (***G***), and (***I***) are Atomic Force Microscopy images, and (***B***), (***D***), (***F***), (***H***), and (***J***) are corresponding Magnetic Force Microscopy images obtained at field values of 0.5 mT, 3.5 mT, 7mT, 15 mT, and 35 mT, respectively, with magnetic fields applied vertical to the sample surface. The circles in (***A-H***) highlight the locations where the magnetic contrast rapidly changes in response to the external magnetic field.

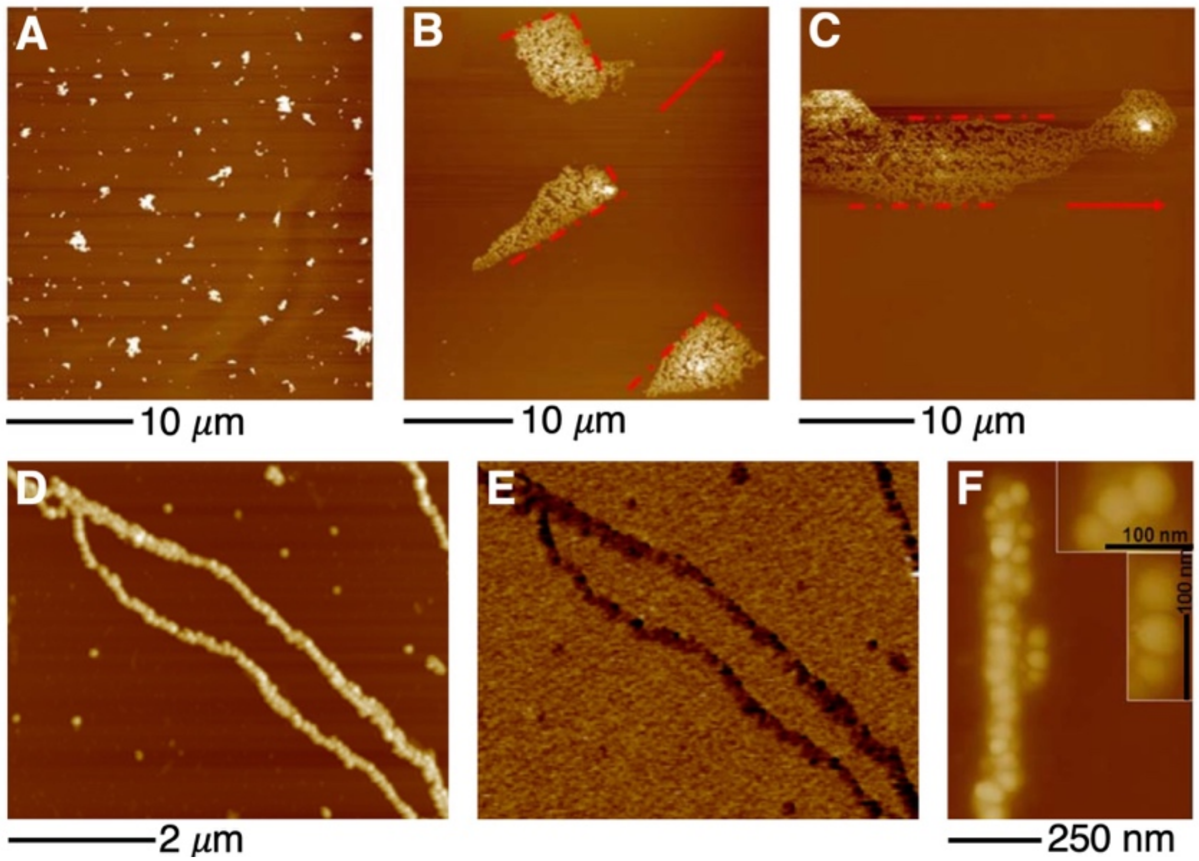

Fig. S3. Microscopy images of aggregates of magnetic nanoparticles dried on a solid surface. (***A-C***) commercial ferrofluid containing 10 nm sized particles dried in (***A***) the absence of external magnetic fields, (***B***) a 20 mT in-plane static magnetic field, (***C***) a 40 mT in-plane static field. The field axis is indicated by the red arrow. (***D-E***) magnetotactic bacteria particle aggregations dried in a 45 mT in-plane static field. Images (***A-D***) were obtained with Atomic Force Microscopy and image (***E***) with Magnetic Force Microscopy. The dark magnetic contrast in the MFM image (***E***) represents attractive interactions between the magnetic tip and the particles. ***(F)*** chains of magnetotactic bacteria particles shown in higher resolution.

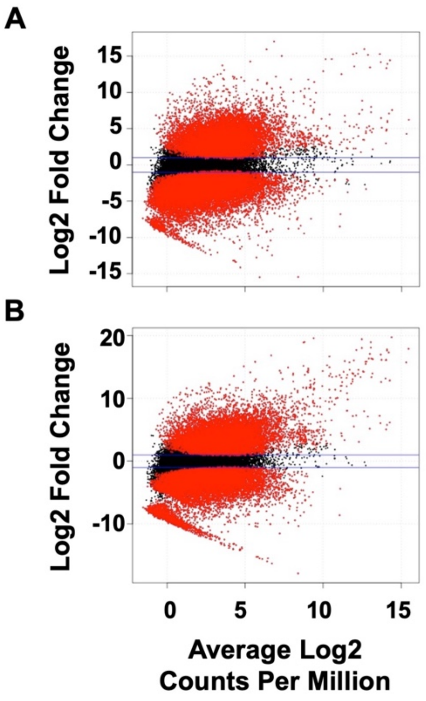

Fig. S4. Chinook salmon (*Oncorhynchus tshawytscha*) transcriptome profiles. MA-plot display of the log2 fold ratio of modeled gene expression values (y-axis) and average log2 counts per million (CPM, x-axis) between (***A***) magnetic cell isolates contrasted to blood and (***B***) magnetic cell isolates contrasted to muscle tissue, with red dots indicating differentially expressed genes (at FDR < 0.05) and black dots indicating no significant difference in gene expression. Blood and muscle transcriptomes (positive y-axis) were modeled using three biological replicates (n=3 fish), while magnetic cell transcriptomes (negative y-axis) were modeled from three replicate magnetic cell isolation experiments, each using pools of three to five sets of olfactory rosettes.

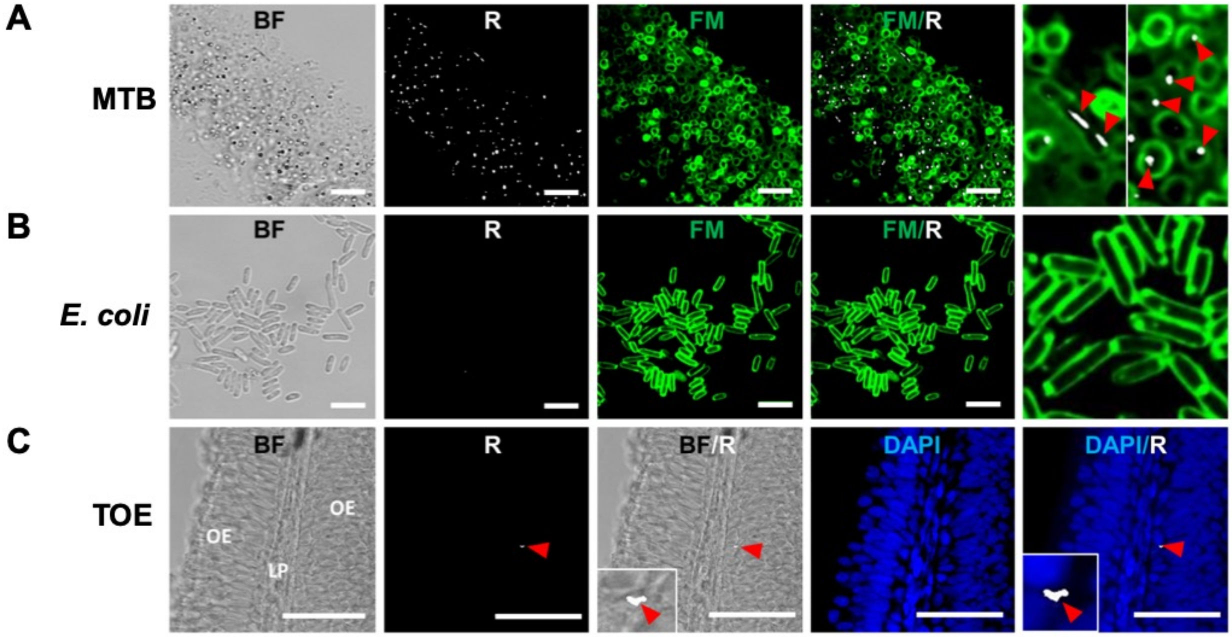

Fig. S5. Visualization of possible biogenic magnetite using confocal microscopy. Reflectance mode of a confocal microscope was used to demonstrate in situ magnetite particles. (***A***) Magnetotactic bacteria (MTB, positive control sample), containing magnetosome chains. Left to right: bright field (BF) image taken at the magnetic north of the drop; same image captured using confocal laser scanning microscope reflectance (R) mode; fluorescent imaging of the lipophilic dye FM 1-43fx (FM), applied to visualize cells' cytoplasmic membranes; FM and R taken in the same focal plane, with FM/R overlap image demonstrating co-localization of reflectance signal with cells, and FM/R close-ups showing reflective structures (red arrows) enclosed by cell membranes. Magnetosome chains in the MTB appear as a linear structure or point (white; reflective structure marked by adjacent red arrow), depending on the chain orientation. Scale bar: 5 μm. (***B***) *Escherichia coli* cells (negative control sample): no reflectance signal was observed despite using the same method as in (***A***). Scale bar: 5 μm. (***C***) Trout olfactory epithelium (TOE) cryosections. Left to right, following same method as (A): BF image of an olfactory lamella with the olfactory epithelium (OE) sandwiching the lamina propria; R mode, featuring a reflective structure (red arrows); fluorescent imaging using DAPI as counterstain instead of FM; BF/R and DAPI/R overlap images show the reflective structure (white) is in the epithelium. Scale bar: 50 μm.

Table S1. Differential gene expression. Numbers genes differentially expressed in four types of Chinook salmon tissue and blood samples. The number of genes differentially and more highly expressed are provided for binary contrasts between magnetic (MAG) olfactory cells, non-magnetic (NM) olfactory cells, blood, and muscle tissues (n=3 transcriptomes per tissue type). Differential gene expression (DEG) is based on FDR adjusted p-values FDR<0.05 or FDR<0.1 (MAG-NM contrast only). The MAG and NM cell isolation experiments (n=3 trials) were conducted using pools of three to five sets of olfactory rosettes.

High Expression	Comparison Tissue	N DEGs
MAG	NM	610 (1,588)
NM	MAG	3681 (5,255)
MAG	Blood	15,964
Blood	MAG	11,766
MAG	Muscle	13,176
Muscle	MAG	12,528
MAG	NM & Blood	429
MAG	NM & Muscle	438
MAG	NM, Blood, & Muscle	367

Table S2. Genomes included in comparative genomics. List of genomes assessed for evaluating presence of distant homologs of magnetotactic bacteria biomineralization genes. Five magnetotactic bacteria taxa (C=*Candidatus*,) in phyla Nitrospira and Proteobacteria (classes Alpha and Delta) were included for comparative purposes. The National Center for Bioinformatics Information (NCBI) RefSeq Accessions and last modified date (LMD) are listed for each genome.

Common name	Scientific name	Superkingdom	Phylum (class)	RefSeq Accession	LMD	N proteins / isoforms	Reference
Roundworm	*Caenorhabditis elegans*	Eukaryota	Nematoda	GCF_000002985.6	8/15/18	28310	(20)
Honeybee	*Apis mellifera*	Eukaryota	Arthropoda	GCF_000002195.4	9/19/17	22451	(21)
Fruit fly	*Drosophila melanogaster*	Eukaryota	Arthropoda	GCF_000001215.4[2]	6/3/18	30493	(22)
Panamanian leafcutter ant	*Acromyrmex echinatior*	Eukaryota	Arthropoda	GCF_000204515.1	6/14/16	20241	(23)
California two-spot octopus	*Octopus bimaculoides*	Eukaryota	Mollusca	GCF_001194135.1	6/9/16	23994	(24)
Little Brown Bat	*Myotis lucifugus*	Eukaryota	Chordata	GCF_000147115.1	1/28/18	43106	(25)
Naked Mole rat	*Heterocephalus glaber*	Eukaryota	Chordata	GCF_000247695.1	9/15/17	60814	(26)
Mouse	*Mus musculus*	Eukaryota	Chordata	GCF_000001635.26	12/26/17	76226	(27)
Human	*Homo sapiens*	Eukaryota	Chordata	GCF_000001405.38	9/10/18	113620	(28)
Zebrafish	*Danio rerio*	Eukaryota	Chordata	GCF_000002035.6	7/16/17	52829	(29)
Zebra finch	*Taeniopygia guttata*	Eukaryota	Chordata	GCF_000002035.6	12/22/17	19943	(30)
Chinook salmon	*Oncorhynchus tshawytscha*	Eukaryota	Chordata	GCF_002872995.1	3/21/18	73277	(31)
Minke whale	*Balaenoptera acutorostrata scammoni*	Eukaryota	Chordata	GCF_000493695.1	6/11/16	34821	(32)
Lokiarchaeota	*Lokiarchaeota spp.*	Archaea	C. Lokiarchaeota	GCA_003662865.1	10/15/18	2849	(33)
MTB	*Magnetococcus marinus* MC-1	Bacteria	Proteobacteria (α)	GCF_000014865.1	12/15/17	3641	(34)
MTB	*Magnetospirillum magneticum* AMB-*1*	Bacteria	Proteobacteria (α)	GCF_000009985.1_ASM998v1	12/15/17	4448	(35)
MTB	*Magnetospirillum magnetotacticum* MS-1	Bacteria	Proteobacteria (α)	GCF_000829825.1	12/9/17	4067	(36)
MTB	*C. Magnetomorum sp.* HK-1	Bacteria	Proteobacteria (δ)	GCA_001292585.1_SAG5*	5/10/16	11022	(37)
MTB	*C. Magnetobacterium casensis*	Bacteria	Nitrospirae	GCF_000714715.1	2/11/17	3034	(38)

* NCBI Genbank Assembly Accession

Table S3. Magnetotactic bacteria magnetosome-associated protein accessions with high numbers of bidirectional matches to eukaryotes. Shown is a summary of bidirectional match patterns between individual magnetotactic bacteria biomineralization proteins and genome contents of 13 eukaryotes (threshold 85% match; full list available from Dataset S2). The Uniprot-KB SWISS-PROT accessions and taxonomic details are provided for prokaryote proteins. The eukaryote genomes considered are: (1) Panamanian leafcutter ant, *Acromyrmex echinatior*, (2) honeybee, *Apis mellifera*, (3) roundworm, *Caenorhabditis elegans*, (4) fruit fly, *Drosophila melanogaster*, (5) California two-spot octopus, *Octopus bimaculoides*, (6) Little Brown Bat, *Myotis lucifugus*, (7) Chinook salmon, *Oncorhynchus tshawytscha,* (8) human, *Homo sapiens*, (9) naked mole-rat, *Heterocephalus glaber*, (10) mouse, *Mus musculus,* (11) Minke whale, *Balaenoptera acutorostrata scammoni*, (12) zebra finch, *Taeniopygia guttata*, and (13) zebrafish, *Danio rerio* (genome details in SI Appendix Table S2). Matches were identified by bidirectional BLASTp, applying a threshold E-value filter of $1e^{-3}$ for the eukaryote-prokaryote query. All but two genes (Mad27-1 and Mad27-2) were categorized as 'universally conserved' in eukaryotes.

**UniprotKB**	**Phylum**	**Bacteria name**	**Gene**	**1**	**2**	**3**	**4**	**5**	**6**	**7**	**8**	**9**	**10**	**11**	**12**	**13**
U5IGN0	Proteobacteria (d)	*Delta proteobacterium ML-1*	Mad9	x	x	x	x	x	x	x	x	x	x	x	x	x
A0A109CXN6	Nitrospirae	*Nitrospirae bacterium HCH-1*	Mad17		x	x	x	x	x	x	x	x	x	x	x	x
A0A109CXJ6	Nitrospirae	Nitrospirae bacterium HCH-1	Mad25					x	x	x	x	x	x	x	x	x
A0A142BTT5	Nitrospirae	*Uncultured Nitrospirae bacterium*	Mad29	x	x	x	x	x	x	x	x	x	x		x	x
A0A0M9E606	Proteobacteria (d)	*Candidatus Magnetomorum sp. HK-1*	Mad27-1			x	x	x	x	x	x	x	x	x	x	x
A0A0M9E220	Proteobacteria (d)	Delta proteobacterium ML-1	Mad27-2		x	x	x	x	x		x	x	x	x	x	
A0A142BTV4	Nitrospirae	*Uncultured Nitrospirae bacterium*	MamA	x	x	x	x	x	x	x	x	x	x	x	x	x
A0A0U5MCU9	Proteobacteria (a)	*Magnetospirillum* sp. XM-1	MamB		x		x		x	x	x	x	x	x	x	x
C5JAJ2	uncultured bact.	Uncultured bacterium	MamE				x		x		x	x	x	x	x	x
A0A0M9ED85	Proteobacteria (d)	*Candidatus Magnetomorum sp. HK-1*	MamE-Cter	x		x	x		x		x	x	x		x	x
A0A1C3RFR4	Proteobacteria (a)	*Candidatus Terasakiella magnetica*	MamH		x	x	x	x	x	x	x	x	x	x	x	
A0A0M9E4Z8	Proteobacteria (d)	*Candidatus Magnetomorum sp. HK-1*	MamK-2		x	x			x	x	x			x	x	x
L0R560	Proteobacteria (d)	*Desulfamplus magnetovallimortis*	MamM			x		x	x	x	x	x	x	x	x	x
A0A0M9E8B0	Proteobacteria (d)	*Candidatus Magnetomorum sp. HK-1*	MamN	x	x	x			x		x		x	x	x	x
Q3BK99	Proteobacteria (a)	*Magnetospirillum gryphiswaldense*	MamU	x					x		x	x	x	x	x	x
A0A0M9E139	Proteobacteria (d)	*Candidatus Magnetomorum sp. HK-1*	Unknown	x	x	x		x	x	x	x	x		x	x	x
A0A088F8P8	Nitrospirae	*Candidatus Magnetobacterium casensis*	Man6		x		x		x	x	x	x	x	x	x	x
Total matches	-----	-----	-----	7	11	12	11	10	17	12	17	15	15	15	17	16

Table S4. Functional annotations. A summary of functional annotations for the whole-genome repertoire of zebrafish (*Danio rerio*) and Chinook salmon (*Oncorhynchus tshawytscha*) protein matches to 'universally conserved' magnetotactic bacteria biomineralization proteins. A total of 229 zebrafish and 181 salmon proteins were matched to zebrafish information identifiers (ZFINs) for functional annotation in PANTHER, Protein Analysis Through Evolutionary Relationships (see Methods for details). Statistically significant, fold-enriched (FE) terms are shown for gene ontology (GO) complete categories biological process (BP), molecular function (MF), and cellular component (CC), using zebrafish as a background genome (# Genes Reference). The number of observed genes (# Obs.) refers to the number of genes mapped to each GO category, while the number of expected genes (# Exp.) is based on the number of genes in the input file relative to the background contents of the zebrafish genome. A complete list of gene ontology annotations is available from Dataset 5.

			Zebrafish				Chinook salmon			
Cat.	GO Complete	# Genes Reference	# Obs	# Exp	FE	FDR	# Obs	# Exp.	FE	FDR
BP	protein refolding	23	10	0.21	48.01	6.34E-10	6	0.16	36.45	1.09E-04
	zinc ion transport	20	8	0.18	44.17	9.75E-08	7	0.14	48.90	1.89E-06
	cellular response to heat	23	9	0.21	43.21	9.56E-09	5	0.16	30.37	1.63E-03
	response to zinc ion	13	5	0.12	42.47	2.26E-04	4	0.09	42.99	4.40E-03
	chaperone cofactor-dependent protein refolding	27	10	0.24	40.90	1.74E-09	6	0.19	31.05	1.98E-04
	mesenchyme morphogenesis	11	4	0.1	40.16	3.93E-03	3	0.08	38.10	4.88E-02
	cellular response to unfolded protein	31	9	0.28	32.06	7.66E-08	5	0.22	22.54	4.79E-03
	protein peptidyl-prolyl isomerization	24	4	0.22	18.41	3.33E-02	4	0.17	23.29	2.45E-02
MF	protein folding chaperone	11	9	0.1	90.35	2.64E-11	5	0.08	63.51	3.20E-05
	misfolded protein binding	14	11	0.13	86.77	1.01E-13	6	0.10	59.88	3.36E-06
	divalent inorganic cation transmembrane transporter activity	20	8	0.18	44.17	4.50E-08	7	0.14	48.90	1.02E-06
	zinc ion transmembrane transporter activity	20	8	0.18	44.17	3.94E-08	7	0.14	48.90	6.81E-07
	Hsp90 protein binding	12	3	0.11	27.61	3.29E-02	4	0.09	46.57	1.48E-03
	peptidyl-prolyl cis-trans isomerase activity	41	7	0.37	18.85	4.89E-05	7	0.29	23.85	2.49E-05
	4 iron, 4 sulfur cluster binding	36	5	0.33	15.34	4.86E-03	4	0.26	15.52	4.52E-02
	insulin-like growth factor binding	32	4	0.29	13.80	3.38E-02	4	0.23	17.46	3.23E-02
	unfolded protein binding	96	12	0.87	13.80	8.50E-08	7	0.69	10.19	2.89E-03
CC	actin-based cell projection	29	5	0.26	19.04	0.017	5	0.21	24.09	6.00E-03

Table S5. **Differentially expressed genes.** A list of Chinook salmon (*Oncorhynchus tshawytscha*) genes differentially and more highly expressed in magnetic olfactory cells compared to non-magnetic cells obtained from the same tissue, and that show distant homology to magnetotactic bacteria biomineralization genes. Chinook salmon gene names are from the RefSeq genome feature table (National Center for Bioinformatic Information Accession GCF_002872995.1). Statistical significance of differential gene expression is adjusted for multiple tests using Benjamini-Hochberg false discover rates (FDR) using a threshold FDR value < 0.10. BLASTp results includes percent (%) match across high scoring segment pairs (HSP), HPS lengths (L), and E-values, showing the best BLASTp match ranked by bitscore (Bit.). Abbreviations: *Candidatus*=C.; Chromosome=Chr; Unclassified=U.

MTB Gene	UniProtKB	MTB Taxonomic Information (Phylum)	Chinook Transcript	Chr.	% Match	L	E-value	Bit.	DEG FDR	Gene name	Homology Class
MamA	A0A142BU53	Uncultured Nitrospirae bacterium (N)	XM_024425584.1	7	31.82	110	7.02E-11	57.8	0.079	lysine-specific demethylase 6A-like	TPR proteins
MamA	W0LN53	*Magnetofaba australis* IT-1 (P, a)	XM_024398845.1	3	31.82	88	2.00E-07	46.6	0.089	peroxisomal targeting signal 1 receptor-like	TPR proteins
MamA	A0A0G3VLC8	Magneto-ovoid bacterium MO-1 (U)	XM_024386466.1	24	34.21	76	4.13E-07	45.1	0.090	interferon-induced protein with tetratricopeptide repeats 5-like	TPR proteins
MamA	A0A142BTV4*	Uncultured Nitrospirae (N)	XM_024397510.1	33	26.71	176	5.58E-05	38.9	0.072	G-protein-signaling modulator 1-like	TPR proteins
MamA	A0A0F2J8Z5	*C. Magnetoovum chiemensis (N)*	XM_024420069.1	5	30.30	132	2.22E-13	64.3	0.005	tetratricopeptide repeat protein 13-like isoform	TPR proteins
MamA	L0R598	*Desulfamplus magnetovallimortis (P, d)*	XM_024378985.1	19	22.60	177	6.68E-10	53.9	0.097	Bardet-Biedl syndrome 4 protein-like isoform	TPR proteins
MamA*	G8IQT3	*Desulfamplus magnetovallimortis* BW-1 (P,d)	XM_024422648.1	5	30.56	72	6.02E-04	32.7	0.011	tetratricopeptide repeat protein 32	TPR proteins
MamA*	G8IQT3	*Desulfamplus magnetovallimortis* BW-1 (P, d)	XM_024411064.1	U	32.90	76	1.15E-07	45.8	0.059	STIP1 homology and U box-containing protein 1-like	TPR proteins
MamB	A0A1C3RH60	*Terasakiella* sp. PR1 (P, a)	XM_024397399.1	33	25.00	112	3.75E-09	52.8	0.044	zinc transporter 5	CDF transporters

MamB	A0A142BTX1	Uncultured Nitrospirae (N)	XM_024391533.1	28	25.68	148	8.50E-04	35	0.072	serine/threonine-protein kinase 17A-like	PKc like superfamily
MamE O-Nter	U5IHV7	Delta proteobacterium ML-1 (P, D)	XM_024434369.1	10	35.38	212	3.84E-32	122	0.097	serine protease HTRA3-like	HtrA-like serine proteases
MamK-2	A0A0M9E4Z8*	*C. Magnetomorum* sp. HK-1 (P, d)	XM_024403462.1	3	19.19	344	6.71E-04	35.4	0.011	actin-related protein 3-like	NBD sugar-kinase HSP70 actin superfamily
Mad9	M1QLL7	Bacterium *FH-1* (U)	XM_024405266.1	U	70.59	17	5.81E-04	35	0.053	ATP-binding cassette sub-family E member 1-like	DMSOR_beta-like superfamily/ FeFe_hydrog_B1 superfamily
Mad25	A0A109CXJ6*	Nitrospirae bacterium HCH-1 (N)	XM_024391317.1	28	23.53	153	3.37E-04	38.1	0.032	rho-associated protein kinase 1-like isoform	Smc superfamily
Mad25	A0A0F2IYL9	*C. Magnetoovum chiemensis* (N)	XM_024442321.1	13	22.01	209	3.86E-05	40	0.055	trafficking kinesin-binding protein 1-like	Smc superfamily
Man6	A0A0F3GW11	*C. Magnetobacterium bavaricum* (N)	XM_024442649.1	13	24.78	222	3.98E-04	38.5	0.032	pleckstrin homology-like domain family B member 1	Smc superfamily
Man6	A0A0F3GW11	*C. Magnetobacterium bavaricum* (N)	XM_024402039.1	1	20.97	267	1.62E-04	39.7	0.057	histone-lysine N-methyltransferase, H3 lysine-79 specific-like	Smc superfamily
Man6	A0A0F3GW11	*C. Magnetobacterium bavaricum* (N)	XM_024380536.1	20	20.10	194	6.60E-06	43.9	0.075	hamartin-like	Smc superfamily

Table S6. Chromosomal locations of candidate genes. Numbers of Chinook (*Oncorhynchus tshawytscha*) salmon protein-coding genes (No. Genes) that show distant homology to universally conserved bacterial magnetosome genes (No. uMGHs). The enrichment of uMGHs on salmon's 34 chromosomes was evaluated using a one-tailed proportion test with Yates' continuity correction and a statistical threshold $p < 0.05$. The summary list highlights the MamABEK core gene combination shown in **bold**. See Methods for details.

Chr.	No. Genes	No. uMGHs	P-value	Summary List	Count by Chromosome
1	1409	18	0.066	**MamABEK**, Mad25, Man6,	5 MamA, 1 MamB, 8 MamE, 2 MamK, 1 Mad25, 1 Man6
2	1085	5	0.901	MamAK, Mad29	3 MamA, 1 MamK, 1 Mad29
3	1471	12	0.532	MamAEK, Mad17	9 MamA, 1 MamE, 1 MamK, 1 Mad17
4	1333	9	0.731	MamAEK	5 MamA, 1 MamE, 1 MamK
5	1551	17	0.205	**MamABEK**, Mad25, Man6	6 MamA, 2 MamB, 3 MamE, 2 MamK, 1 Mad25, 3 Man6
6	1233	21	**0.001**	MamAEK, Mad25, Man6	9 MamA, 4 MamE, 3 MamK, 2 Mad25, 2 Man6
7	1454	13	0.500	MamABE, Mad17, Man6	6 MamA, 2 MamB, 1 MamE, 1 Mad17, 2 Man6,
8	1135	12	0.301	**MamABEK**, MamH, Mad9, Mad17, Man6	4 MamA, 1 MamB, 1 MamE, 2 MamK, 1 MamH, 1 Mad9, 1 Mad17, 1 Man6
9	1707	19	0.170	**MamABEK**	8 MamA, 1 MamB, 4 MamE, 6 MamK
10	1166	10	0.500	MamAEK, Man6, Mad25	5 MamA , 1 MamE, 1 MamK, 1 Mad25, 2 Man6
11	844	10	0.211	MamABK, Mad25	5 MamA, 1 MamB, 2 MamK, 1 Mad25,
12	1299	10	0.594	MamAEK	6 MamA, 3 MamE, 1 MamK
13	1410	9	0.786	MamAK, MamH, ManN, Mad25, Man6	3 MamA, 2 MamK, 1 MamH, 1 MamN, 1 Mad25, 1 Man6
14	1042	6	0.803	**MamABEK**, Mad17	2 MamA, 1 MamB, 1 MamE, 1 MamK, 1 Mad17
15	737	7	0.485	MamAK, ManN, Man6	3 MamA, 2 MamK, 1 MamN, 1 Man6
16	1154	7	0.789	MamAEK, MamH	2 MamA, 2 MamE, 1 MamH, 1 MamK
17	446	6	0.204	MamAB, MamN, Man6	2 MamA, 1 MamB, 1 MamN, 2 Man6
18	612	8	0.171	MamABK, MamH, Man6	3 MamA, 1 MamB, 2 MamK, 1 MamH, 1 Man6
19	838	8	0.468	MamAK	5 MamA, 2 MamK
20	852	11	0.127	MamAEK, Mad17, Man6	4 MamA, 3 MamE, 2 MamK, 1 Mad17, 1 Man6
21	620	6	0.481	**MamABEK**	1 MamA, 1 MamB, 2 MamE, 2 MamK
22	856	4	0.861	MamAE, Mad27	1 MamA, 1 MamE, 1 Mad17
23	501	2	0.814	MamAK	1 MamA, 1 MamK
24	499	8	0.064	MamAE, Mad29	4 MamA, 3 MamE, 1 Mad29
25	660	7	0.375	MamAEK	2 MamA, 3 MamE, 2 MamK
26	622	7	0.319	MamAEK	5 MamA, 1 MamE, 1 MamK
27	474	6	0.248	MamEA	4 MamA, 2 MamE,
28	764	8	0.369	MamABE, Mad25, Man6	2 MamA, 1 MamB, 1 MamE, 1 Mad25, 3 Man6
29	465	5	0.410	MamAE	4 MamA, 1 MamE
30	821	3	0.914	MamAK	2 MamA, 1 MamK
31	663	2	0.914	MamA, MamH	1 MamE, 1 MamH

32	282	3	0.488	MamAK, Mad9	1 MamA, 1 MamK, 1 Mad29
33	753	10	0.123	**MamABEK**, Mad17	5 MamA, 1 MamB, 1 MamE, 2 MamK, 1 Mad17
34	382	2	0.675	Mad9	2 Mad9

SI References

1. S. Mann, N. H. Sparks, M. M. Walker, J. L. Kirschvink, Ultrastructure, morphology and organization of biogenic magnetite from sockeye salmon, *Oncorhynchus nerka*: implications for magnetoreception. *J. Exp. Biol.* **140**, 35–49 (1988).
2. B. P. Weiss, *et al.*, Ferromagnetic resonance and low-temperature magnetic tests for biogenic magnetite. *Earth Planet. Sci. Lett.* **224**, 73–89 (2004).
3. R. E. Kopp, *et al.*, Chains, clumps, and strings: Magnetofossil taphonomy with ferromagnetic resonance spectroscopy. *Earth Planet. Sci. Lett.* **247**, 10–25 (2006).
4. M. Charilaou, M. Winklhofer, A. U. Gehring, Simulation of ferromagnetic resonance spectra of linear chains of magnetite nanocrystals. *J. Appl. Phys.* **109**, 093903 (2011).
5. L. Chang, *et al.*, Magnetic detection and characterization of biogenic magnetic minerals: A comparison of ferromagnetic resonance and first-order reversal curve diagrams. *J. Geophys. Res. Solid Earth* **119**, 6136–6158 (2014).
6. C. E. Diebel, R. Proksch, C. R. Green, P. Neilson, M. M. Walker, Magnetite defines a vertebrate magnetoreceptor. *Nature* **406**, 299–302 (2000).
7. S. H. K. Eder, *et al.*, Magnetic characterization of isolated candidate vertebrate magnetoreceptor cells. *Proc. Natl. Acad. Sci. U. S. A.* **109**, 12022–12027 (2012).
8. H. Vali, J. L. Kirschvink, "Observations of magnetosome organization, surface structure, and iron biomineralization of undescribed magnetic bacteria: evolutionary speculations" in *Iron Biominerals*, Richard B. Frankel and Richard P. Blakemore, Ed. (Springer, Boston, MA, 1991), pp. 97–115.
9. A. Komeili, Molecular mechanisms of compartmentalization and biomineralization in magnetotactic bacteria. *FEMS Microbiol. Rev.* **36**, 232–255 (2012).
10. D. Murat, A. Quinlan, H. Vali, A. Komeili, Comprehensive genetic dissection of the magnetosome gene island reveals the step-wise assembly of a prokaryotic organelle. *Proc. Natl. Acad. Sci. U. S. A.* **107**, 5593–5598 (2010).
11. G. L. Blatch, M. Lässle, The tetratricopeptide repeat: A structural motif mediating protein-protein interactions. *BioEssays* **21**, 932–939 (1999).
12. K. Grünberg, *et al.*, Biochemical and Proteomic Analysis of the Magnetosome Membrane in *Magnetospirillum gryphiswaldense*. *Appl. Environ. Microbiol.* **70**, 1040–1050 (2004).
13. A. Komeili, Z. Li, D. K. Newman, G. J. Jensen, Magnetosomes are cell membrane imaginations organized by the actin-like protein MamK. *Science* **311**, 242–245 (2006).
14. H. N. Higgs, T. D. Pollard, Activation by Cdc42 and PIP2 of Wiskott-Aldrich Syndrome protein (WASp) stimulates actin nucleation by Arp2/3 complex. *J. Cell Biol.* **150**, 1311–1320 (2000).
15. R. D. Mullins, J. A. Heuser, T. D. Pollard, The interaction of Arp2/3 complex with actin: Nucleation, high affinity pointed end capping, and formation of branching networks of filaments. *Proc. Natl. Acad. Sci. U. S. A.* **95**, 6181–6186 (1998).
16. M. I. Siponen, G. Adryanczyk, N. Ginet, P. Arnoux, D. Pignol, Magnetochrome: A c-type cytochrome domain specific to magnetotatic bacteria in *Biochemical Society Transactions*, (2012), pp. 1319–1323.
17. E. R. Frawley, F. C. Fang, The ins and outs of bacterial iron metabolism. *Mol. Microbiol.* **93**, 609–616 (2014).
18. W. Lin, *et al.*, Origin of microbial biomineralization and magnetotaxis during the Archean. *Proc. Natl. Acad. Sci.* **114**, 2171–2176 (2017).
19. W. Lin, *et al.*, Genomic expansion of magnetotactic bacteria reveals an early common origin of magnetotaxis with lineage-specific evolution. *ISME J.* **12** (2018).
20. The *C. elegans* Sequencing Consortium, Genome sequence of the nematode *C. elegans*: A platform for investigating biology. *Science* **282**, 2012–2018 (1998).
21. G. M. Weinstock, *et al.*, Insights into social insects from the genome of the honeybee *Apis*

mellifera. *Nature* **443**, 931–934 (2006).
22. M. D. Adams, *et al.*, The genome sequence of *Drosophila melanogaster*. *Science* **287**, 2185–2195 (2000).
23. S. Nygaard, *et al.*, The genome of the leaf-cutting ant *Acromyrmex echinatior* suggests key adaptations to advanced social life and fungus farming. *Genome Res.* **21**, 1339–1348 (2011).
24. C. B. Albertin, *et al.*, The octopus genome and the evolution of cephalopod neural and morphological novelties. *Nature* **524**, 220–224 (2015).
25. K. Lindblad-Toh, *et al.*, A high-resolution map of human evolutionary constraint using 29 mammals. *Nature* **478**, 476–482 (2011).
26. E. B. Kim, *et al.*, Genome sequencing reveals insights into physiology and longevity of the naked mole rat. *Nature* **479**, 223–227 (2011).
27. D. M. Church, *et al.*, Lineage-specific biology revealed by a finished genome assembly of the mouse. *PLoS Biol.* **7**, e1000112 (2009).
28. E. S. Lander, *et al.*, Initial sequencing and analysis of the human genome. *Nature* **409**, 860–921 (2001).
29. K. Howe, *et al.*, The zebrafish reference genome sequence and its relationship to the human genome. *Nature* **496**, 498–503 (2013).
30. W. C. Warren, *et al.*, The genome of a songbird. *Nature* **496**, 498–503 (2010).
31. K. A. Christensen, *et al.*, Chinook salmon (*Oncorhynchus tshawytscha*) genome and transcriptome. *PLoS One* **13**, e0195461 (2018).
32. H. S. Yim, *et al.*, Minke whale genome and aquatic adaptation in cetaceans. *Nat. Genet.* **46**, 88–92 (2014).
33. N. Dombrowski, K. W. Seitz, A. P. Teske, B. J. Baker, Genomic insights into potential interdependencies in microbial hydrocarbon and nutrient cycling in hydrothermal sediments. *Microbiome* **5**, 106 (2017).
34. D. A. Bazylinski, *et al.*, *Magnetococcus marinus* gen. nov., sp. nov., a marine, magnetotactic bacterium that represents a novel lineage (Magnetococcaceae fam. nov., Magnetococcales ord. nov.) at the base of the Alphaproteobacteria. *Int. J. Syst. Evol. Microbiol.* **63**, 801–808 (2013).
35. T. Matsunaga, *et al.*, Complete genome sequence of the facultative anaerobic magnetotactic bacterium *Magnetospirillum* sp. strain AMB-1. *DNA Res.* **12**, 157–166 (2005).
36. M. D. Smalley, G. K. Marinov, L. E. Bertani, G. DeSalvo, Genome sequence of *Magnetospirillum magnetotacticum* strain MS-1. *Genome Announc.* **3**, e00233-15 (2016).
37. S. Kolinko, M. Richter, F. O. Glöckner, A. Brachmann, D. Schüler, Single-cell genomics reveals potential for magnetite and greigite biomineralization in an uncultivated multicellular magnetotactic prokaryote. *Environ. Microbiol. Rep.* **6**, 524–531 (2014).
38. W. Lin, *et al.*, Genomic insights into the uncultured genus "*Candidatus Magnetobacterium*" in the phylum Nitrospirae. *ISME J.* **8**, 2463–2477 (2014).
