# Supplementary material for: Conservation of magnetite biomineralization genes in all domains of life and implications for magnetic sensing
Source: Proc Natl Acad Sci U S A. 2022 Jan 10;119(3):e2108655119. doi: 10.1073/pnas.2108655119 (PMC8784154; doi:10.1073/pnas.2108655119)
Supplement: Supplementary File [file pnas.2108655119.sd01.pdf]

Dataset S1. **Gene ontologies for differentially expressed genes**. Functional annotation results for Chinook salmon (*Oncorhynchus tshawytscha*) genes differentially and more highly expressed in magnetic olfactory cells contrasted to non-magnetic cells isolated from the same tissue and having significant overrepresentation in gene ontology (GO) categories. Analyses were conducted in PANTHER, Protein Analysis Through Evolutionary Relationships, applying the built-in Benjamini-Hochberg false discovery rate (FDR $P < 0.05$) to correct for multiple tests and listing all categories identified as significant per program default settings. Results are shown for GO complete categories biological process (BP), molecular function (MF), and cellular component (CC). Shading is used to depict individual GO category groupings, with sub-category hierarchy (H) indicated using a numeric scale: zero indicates the most specific sub-category assignment, and incrementally higher numbers indicates the next-broader-level within the ontology hierarchy (multiple equal hierarchies can be present). A total of 1,588 differentially expressed transcripts were matched to zebrafish protein homologs, resulting in assignment of 1,333 zebrafish ZDB-Gene identifiers to enable use of zebrafish (*Danio rerio*) as a background genome. The number observed (No. Obs.) refers to the number of DEGs that mapped to each GO category, while the number of expected genes (No. exp.) is modeled by considering the number of genes in the input file and the background contents of the zebrafish genome. Fold-enrichment (FE) is calculated by No. obs. / No. exp.

GO	H	GO Complete	No. genes zebrafish genome	No. obs.	No. exp.	FE	Raw p-value	FDR
BP	0	anatomical structure maturation	53	11	2.38	4.63	0.0001	0.0392
	1	developmental maturation	71	12	3.18	3.77	0.0002	0.0486
	1	anatomical structure development	3908	223	175.24	1.27	0.0002	0.0512
	2	developmental process	4049	235	181.56	1.29	0.0000	0.0301
	0	cell maturation	50	10	2.24	4.46	0.0002	0.0481
	1	cellular developmental process	2130	132	95.51	1.38	0.0003	0.0498
	2	cellular process	12019	605	538.95	1.12	0.0001	0.0435
	2	cell differentiation	2080	130	93.27	1.39	0.0002	0.0462
	0	mitotic cell cycle	295	32	13.23	2.42	0.0000	0.0165
	0	ubiquitin-dependent protein catabolic process	352	33	15.78	2.09	0.0002	0.0508
	1	primary metabolic process	5656	317	253.62	1.25	0.0000	0.0145
	2	metabolic process	6485	348	290.80	1.20	0.0002	0.0504
	2	organic substance metabolic process	5966	325	267.53	1.21	0.0001	0.0472
	2	nitrogen compound metabolic process	5198	286	233.09	1.23	0.0002	0.0498
	1	cellular protein metabolic process	2801	169	125.60	1.35	0.0001	0.0452
	2	cellular macromolecule metabolic process	3555	206	159.41	1.29	0.0002	0.0520

GO	H	GO Complete	No. genes zebrafish genome	No. obs.	No. exp.	FE	Raw p-value	FDR
	3	cellular metabolic process	5582	306	250.31	1.22	0.0001	0.0479
	0	regulation of protein modification process	516	45	23.14	1.94	0.0001	0.0405
	1	regulation of protein metabolic process	827	62	37.08	1.67	0.0002	0.0513
	2	regulation of primary metabolic process	2790	191	125.11	1.53	0.0000	0.0000
	3	regulation of metabolic process	3023	205	135.56	1.51	0.0000	0.0000
	4	regulation of biological process	7993	420	358.42	1.17	0.0002	0.0490
	5	biological regulation	8691	452	389.72	1.16	0.0002	0.0504
	2	regulation of nitrogen compound metabolic process	2741	184	122.91	1.50	0.0000	0.0001
	1	regulation of cellular metabolic process	2846	192	127.62	1.50	0.0000	0.0000
	2	regulation of macromolecule metabolic process	2867	195	128.56	1.52	0.0000	0.0000
	0	regulation of phosphorylation	453	39	20.31	1.92	0.0003	0.0488
	1	regulation of phosphate metabolic process	482	41	21.61	1.90	0.0003	0.0516
	2	regulation of phosphorus metabolic process	482	41	21.61	1.90	0.0003	0.0505
	0	regulation of transcription, DNA-templated	1815	116	81.39	1.43	0.0002	0.0482
	1	regulation of cellular macromolecule biosynthetic process	1992	126	89.32	1.41	0.0002	0.0512
	2	regulation of cellular biosynthetic process	2031	129	91.07	1.42	0.0001	0.0446
	3	regulation of biosynthetic process	2054	129	92.10	1.40	0.0002	0.0492
	2	regulation of macromolecule biosynthetic process	2001	126	89.73	1.40	0.0002	0.0478
	1	regulation of gene expression	2190	146	98.2	1.49	0.0000	0.0043
	1	regulation of nucleic acid-templated transcription	1816	116	81.43	1.42	0.0002	0.0473
	2	regulation of RNA biosynthetic process	1817	116	81.48	1.42	0.0002	0.0455
	3	regulation of RNA metabolic process	1960	130	87.89	1.48	0.0000	0.0157
	4	regulation of nucleobase-containing compound metabolic process	2044	135	91.66	1.47	0.0000	0.0152
	0	cellular protein modification process	2229	141	99.95	1.41	0.0001	0.0359
	1	protein modification process	2229	141	99.95	1.41	0.0001	0.0383
	2	macromolecule modification	2357	146	105.69	1.38	0.0001	0.0447
	0	animal organ development	2248	139	100.8	1.38	0.0002	0.0514
	1	system development	3154	190	141.43	1.34	0.0000	0.0294
	2	multicellular organism development	3591	209	161.03	1.30	0.0001	0.0487

GO	H	GO Complete	No. genes zebrafish genome	No. obs.	No. exp.	FE	Raw p-value	FDR
CC	0	cyclin-dependent protein kinase holoenzyme complex	32	8	1.43	5.58	0.0003	0.0151
	1	serine/threonine protein kinase complex	69	11	3.09	3.56	0.0006	0.0316
	2	protein kinase complex	82	13	3.68	3.54	0.0002	0.0135
	3	transferase complex, transferring phosphorus-containing groups	216	25	9.69	2.58	0.0001	0.0053
	4	transferase complex	593	55	26.59	2.07	0.0000	0.0003
	5	catalytic complex	1035	77	46.41	1.66	0.0000	0.0032
	6	protein-containing complex	3792	229	170.04	1.35	0.0000	0.0006
	1	intracellular part	10545	561	472.85	1.19	0.0000	0.0001
	2	intracellular	10545	561	472.85	1.19	0.0000	0.0001
	3	cell part	13031	660	584.33	1.13	0.0000	0.0011
	4	cell	13031	660	584.33	1.13	0.0000	0.0012
	0	SCF ubiquitin ligase complex	50	9	2.24	4.01	0.0009	0.0425
	1	cullin-RING ubiquitin ligase complex	104	14	4.66	3.00	0.0005	0.0296
	0	nuclear body	92	14	4.13	3.39	0.0002	0.0121
	1	nucleoplasm part	426	38	19.10	1.99	0.0002	0.0124
	2	nucleoplasm	593	48	26.59	1.81	0.0002	0.0139
	3	nuclear lumen	979	70	43.90	1.59	0.0003	0.0182
	4	organelle part	4092	240	183.49	1.31	0.0000	0.0018
	5	organelle	8339	455	373.93	1.22	0.0000	0.0002
	4	intracellular organelle	8155	450	365.68	1.23	0.0000	0.0001
	3	nuclear part	1389	98	62.29	1.57	0.0000	0.0020
	4	nucleus	4461	260	200.04	1.30	0.0000	0.0010
	5	intracellular membrane-bounded organelle	6785	380	304.25	1.25	0.0000	0.0002
	6	membrane-bounded organelle	7138	398	320.08	1.24	0.0000	0.0002
	4	intracellular organelle part	3926	236	176.05	1.34	0.0000	0.0006
	0	bounding membrane of organelle	739	54	33.14	1.63	0.0009	0.0433
	1	organelle membrane	1095	74	49.10	1.51	0.0009	0.0432
MF	0	ubiquitin-protein transferase activity	322	34	14.44	2.35	0.0000	0.0054
	1	ubiquitin-like protein transferase activity	336	35	15.07	2.32	0.0000	0.0055

GO	H	GO Complete	No. genes zebrafish genome	No. obs.	No. exp.	FE	Raw p-value	FDR
	2	catalytic activity, acting on a protein	2605	162	116.81	1.39	0.0000	0.0119
	2	transferase activity	2769	174	124.17	1.40	0.0000	0.0057
	0	protein binding	3984	244	178.65	1.37	0.0000	0.0005
	1	binding	11537	620	517.34	1.20	0.0000	0.0000
	0	organic cyclic compound binding	6184	345	277.30	1.24	0.0000	0.0057
	0	heterocyclic compound binding	6124	341	274.61	1.24	0.0000	0.0053
