# Supplementary material for: Conservation of magnetite biomineralization genes in all domains of life and implications for magnetic sensing
Source: Proc Natl Acad Sci U S A. 2022 Jan 10;119(3):e2108655119. doi: 10.1073/pnas.2108655119 (PMC8784154; doi:10.1073/pnas.2108655119)
Supplement: Supplementary File [file pnas.2108655119.sd02.pdf]

Dataset S2. List of magnetotactic bacteria (MTB) magnetosome-associated protein UniProt accessions (n=601) and names (n=108, including "Unknown") used to evaluate distant homology with genes of eukaryote and Lokiarchaeota. A bidirectional BLASTp match between an individual MTB protein and a gene within genome contents of 13 eukaryotes, Lokiarchaeota, and five MTB, is indicated by x. The number (count) and percent of matches (% match) across the 13 eukaryote genomes are tallied as presence absence data. Columns labeled 1-13 are eukaryotes: (1) Panamanian leafcutter ant, *Acromyrmex echinatior*, (2) honeybee, *Apis mellifera*, (3) roundworm, *Caenorhabditis elegans*, (4) fruit fly, *Drosophila melanogaster*, (5) California two-spot octopus, *Octopus bimaculoides*, (6) Little Brown Bat, *Myotis lucifugus*, (7) Chinook salmon, *Oncorhynchus tshawytscha,* (8) human, *Homo sapiens*, (9) naked mole-rat, *Heterocephalus glaber*, (10) mouse, *Mus musculus,* (11) Minke whale, *Balaenoptera acutorostrata scammoni*, (12) zebra finch, *Taeniopygia guttata*, and (13) zebrafish, *Danio rerio*. Column 14 is the asgard clade of Archaea Lokiarchaeota (A), and columns 15-19 are magnetotactic bacteria: (15) *Candidatus magnetobacterium casensis* (Nitrospirae), (16) *Candidatus Magnetomorum* sp. HK-1 (Deltaproteobacteria), and three Alphaproteobacteria (17) *Magnetococcus marinus* MC-1, (18) *Magnetospirillum magneticum* AMB-1, and (19) *Magnetospirillum magnetotacticum* MS1. Bidirectional BLASTp matches were filtered for E <$10e^{-3}$ in the eukaryote/Archaea to bacteria magnetosome-associated protein comparison.

		Eukaryotes													A	Magnetotactic bacteria						
UniProt Accession	Protein name	1	2	3	4	5	6	7	8	9	10	11	12	13	14	15	16	17	18	19	Count	% Match
K7Y619_9DELT	Dam21		x									x									2	0.15
K7YKX6_9DELT	Dam22																				0	0.00
K7Y7J2_9DELT	Dam28			x																	1	0.08
K7Y0C1_9DELT	FeoB	x			x			x					x	x			x				5	0.38
A0A0M9ECT4_9DELT	Mad1		x														x				1	0.08
M1RA87_9BACT	Mad1								x	x											2	0.15
U5IGI8_9DELT	Mad1																				0	0.00
A0A088FCJ2_9BACT	Mad10																				0	0.00
A0A0N0V130_9DELT	Mad10				x												x				1	0.08
A0A125P3F7_9BACT	Mad10					x		x													2	0.15
A0A142BTU7_9BACT	Mad10																				0	0.00
A0A142BTW5_9BACT	Mad10																				0	0.00
A0A142BU09_9BACT	Mad10							x				x				x					2	0.15
A0A142BU45_9BACT	Mad10							x		x											2	0.15
M1QLM3_9BACT	Mad10																		x		0	0.00
U5IHW3_9DELT	Mad10																				0	0.00
A0A0F2J479_9BACT	Mad11																				0	0.00
A0A0F3GMB8_9BACT	Mad11																				0	0.00
A0A0H3Y7F5_9BACT	Mad11							x													1	0.08

		Eukaryotes													A	Magnetotactic bacteria						
**UniProt Accession**	**Protein name**	**1**	**2**	**3**	**4**	**5**	**6**	**7**	**8**	**9**	**10**	**11**	**12**	**13**	**14**	**15**	**16**	**17**	**18**	**19**	**Count**	**% Match**
A0A0N0D3V2_9DELT	Mad11																x				0	0.00
U5IGJ8_9DELT	Mad11																				0	0.00
A0A0N0D2W6_9DELT	Mad12																x				0	0.00
A0A0N0D263_9DELT	Mad17																x				0	0.00
A0A109CXN6_9BACT	Mad17		x	x	x	x	x	x	x	x	x	x	x	x		x		x	x	x	12	0.92
M1RG59_9DELT	Mad17	x	x	x		x	x								x						5	0.38
M1RMK5_9BACT	Mad17							x		x		x	x								4	0.31
A0A088F9L7_9BACT	Mad2															x					0	0.00
A0A0F2J6P0_9BACT	Mad2																				0	0.00
A0A0F3GSK4_9BACT	Mad2																				0	0.00
A0A109CXB8_9BACT	Mad2																				0	0.00
A0A142BTX2_9BACT	Mad2																				0	0.00
A0A142BU16_9BACT	Mad2																				0	0.00
A0A142BU52_9BACT	Mad2							x													1	0.08
M1RMJ1_9BACT	Mad2																x				0	0.00
A0A0F3GSJ9_9BACT	Mad20													x							1	0.08
M1RMK8_9BACT	Mad20																				0	0.00
U5IHY0_9DELT	Mad20						x			x		x									3	0.23
M1RVI3_9BACT	Mad21																				0	0.00
U5IGN4_9DELT	Mad21								x			x									2	0.15
A0A0M9E5G1_9DELT	Mad21-1		x				x		x	x	x	x					x				6	0.46
A0A0N1J199_9DELT	Mad22	x					x	x		x						x	x				4	0.31
M1RG91_9BACT	Mad22												x	x							2	0.15
U5IGK2_9DELT	Mad22				x																1	0.08
A0A0F2J1L8_9BACT	Mad23																				0	0.00
A0A0F3GSJ4_9BACT	Mad23							x						x							2	0.15
A0A120DCN2_9BACT	Mad23																				0	0.00
A0A142BTY1_9BACT	Mad23			x	x					x	x	x									5	0.38
A0A142BU25_9BACT	Mad23				x										x	x			x		1	0.08
M1RA76_9BACT	Mad23		x					x													2	0.15
U5IHW5_9DELT	Mad23	x		x	x	x				x	x	x		x							8	0.62
A0A0M9E510_9DELT	Mad23-1	x	x	x	x	x	x	x			x			x			x				9	0.69
A0A0M9E2C3_9DELT	Mad23-2																x				0	0.00
A0A088F9L1_9BACT	Mad23-I		x																		1	0.08
A0A088F8I8_9BACT	Mad23-II							x			x					x					2	0.15

								Eukaryotes							A			Magnetotactic bacteria				
UniProt Accession	Protein name	1	2	3	4	5	6	7	8	9	10	11	12	13	14	15	16	17	18	19	Count	% Match
A0A088FCD9_9BACT	Mad24						x				x					x					2	0.15
A0A0F2J410_9BACT	Mad24																x				0	0.00
A0A0F3GS82_9BACT	Mad24																				0	0.00
A0A120DCN3_9BACT	Mad24			x					x										x		2	0.15
A0A142BTY2_9BACT	Mad24																				0	0.00
A0A142BU26_9BACT	Mad24																				0	0.00
M1QLM7_9BACT	Mad24	x				x		x				x									4	0.31
M1RMI3_9BACT	Mad24																				0	0.00
A0A0M9E319_9DELT	Mad24-2									x			x				x				2	0.15
A0A0N0D2V7_9DELT	Mad24-2						x		x			x		x			x				4	0.31
A0A088F8P3_9BACT	Mad25													x		x					1	0.08
A0A0F2IYL9_9BACT	Mad25	x			x				x												3	0.23
A0A0F3GSE1_9BACT	Mad25						x										x				1	0.08
A0A109CXJ6_9BACT	Mad25					x	x	x	x	x	x	x	x	x							9	0.69
A0A142BTY3_9BACT	Mad25						x					x									2	0.15
A0A142BU27_9BACT	Mad25																				0	0.00
M1RML3_9BACT	Mad25	x	x	x			x		x			x									6	0.46
M1RVF5_9BACT	Mad25					x									x						1	0.08
A0A088FCH5_9BACT	Mad26					x	x							x		x					3	0.23
A0A0F2IZM6_9BACT	Mad26																				0	0.00
A0A0F3GW06_9BACT	Mad26												x								1	0.08
A0A109CUC4_9BACT	Mad26		x																		1	0.08
A0A142BTY4_9BACT	Mad26					x				x	x		x								4	0.31
A0A142BU28_9BACT	Mad26																				0	0.00
M1RVI8_9BACT	Mad26							x													1	0.08
A0A0M9E4Z0_9DELT	Mad26-1						x										x				1	0.08
A0A0M9E2N1_9DELT	Mad26-2				x			x				x					x				3	0.23
M1RVJ3_9BACT	Mad27	x			x	x								x	x	x					4	0.31
A0A0M9E606_9DELT	Mad27-1			x	x	x	x	x	x	x	x	x	x	x			x	x			11	0.85
A0A0M9E220_9DELT	Mad27-2		x	x	x	x	x		x	x	x	x	x	x		x	x		x	x	11	0.85
A0A088FCJ5_9BACT	Mad28									x		x				x					2	0.15
A0A109CY44_9BACT	Mad28																				0	0.00
A0A142BTT3_9BACT	Mad28										x										1	0.08
A0A142BTV9_9BACT	Mad28																				0	0.00
A0A142BU04_9BACT	Mad28																				0	0.00

															A	Magnetotactic bacteria						
							Eukaryotes															
UniProt Accession	Protein name	1	2	3	4	5	6	7	8	9	10	11	12	13	14	15	16	17	18	19	Count	% Match
A0A142BU40_9BACT	Mad28				x		x	x													3	0.23
M1RA80_9BACT	Mad28	x																			1	0.08
A0A0F2J3Z6_9BACT	Mad28-1							x													1	0.08
A0A0F3GQM5_9BACT	Mad28-1			x					x		x	x									4	0.31
A0A0N0D423_9DELT	Mad28-1																x				0	0.00
A0A0F2J7B2_9BACT	Mad28-2		x																		1	0.08
A0A0F3GM90_9BACT	Mad28-2															x					0	0.00
A0A0M9E946_9DELT	Mad28-2		x		x	x		x			x	x	x	x			x				8	0.62
A0A109CXB0_9BACT	Mad28-2																				0	0.00
A0A0F3GWQ5_9BACT	Mad28-3																				0	0.00
A0A0F3GXB1_9BACT	Mad28-5									x											1	0.08
A0A0F3GRG4_9BACT	Mad28-6									x	x										2	0.15
A0A0F2J572_9BACT	Mad29	x										x			x						2	0.15
A0A125P3I7_9BACT	Mad29													x							1	0.08
A0A142BTT5_9BACT	Mad29	x	x	x	x	x	x	x	x	x	x		x	x			x	x	x	x	12	0.92
A0A142BTW1_9BACT	Mad29																				0	0.00
M1RML8_9BACT	Mad29																				0	0.00
A0A0F3GWM7_9BACT	Mad29-1						x			x			x								3	0.23
A0A0F3GL85_9BACT	Mad29-2														x	x					0	0.00
A0A0F2J6W1_9BACT	Mad30							x													1	0.08
A0A0F3GN75_9BACT	Mad30															x					0	0.00
A0A0M9DZT2_9DELT	Mad30																x		x	x	0	0.00
A0A109CXN1_9BACT	Mad30																				0	0.00
M1RA46_9DELT	Mad30																				0	0.00
M1RG63_9BACT	Mad30								x										x	x	1	0.08
M1RVH8_9BACT	Mad30														x						0	0.00
A0A0F2J3L5_9BACT	Mad31																				0	0.00
A0A0F3GSF2_9BACT	Mad31																				0	0.00
A0A109CX41_9BACT	Mad31																				0	0.00
A0A142BTV1_9BACT	Mad31																x				0	0.00
A0A142BTW9_9BACT	Mad31																				0	0.00
A0A142BU13_9BACT	Mad31															x					0	0.00
A0A142BU49_9BACT	Mad31																				0	0.00
A0A0N0V2S1_9DELT	Mad4					x											x				1	0.08
M1RVG8_9BACT	Mad4																				0	0.00

		Eukaryotes													A	Magnetotactic bacteria						
UniProt Accession	Protein name	1	2	3	4	5	6	7	8	9	10	11	12	13	14	15	16	17	18	19	Count	% Match
U5IIP2_9DELT	Mad4																				0	0.00
A0A0N0D573_9DELT	Mad6																x				0	0.00
M1RG77_9BACT	Mad6											x			x	x			x	x	1	0.08
U5IHV9_9DELT	Mad6															x		x			0	0.00
A0A0M9E7Q4_9DELT	Mad7																x				0	0.00
M1RVH3_9BACT	Mad7																				0	0.00
U5IIP3_9DELT	Mad7																				0	0.00
A0A0M9E7N4_9DELT	Mad8																x				0	0.00
M1RG82_9BACT	Mad8																				0	0.00
A0A0M9E474_9DELT	Mad9	x	x	x		x	x								x	x	x	x	x	x	5	0.38
M1QLL7_9BACT	Mad9															x		x		x	0	0.00
U5IGN0_9DELT	Mad9	x	x	x	x	x	x	x	x	x	x	x	x	x					x	x	13	1.00
A0A088FCE6_9BACT	MamA															x					0	0.00
A0A0F2J8Z5_9BACT	MamA							x										x		x	1	0.08
A0A0F3GS91_9BACT	MamA																				0	0.00
A0A0G3VLC8_9BACT	MamA				x			x						x				x			3	0.23
A0A0M9EAQ0_9DELT	MamA						x	x	x		x	x	x	x		x	x	x			7	0.54
A0A0M9EDA7_9DELT	MamA																x				0	0.00
A0A0U5MIJ2_9PROT	MamA									x									x	x	1	0.08
A0A109CX17_9BACT	MamA	x						x	x		x			x							5	0.38
A0A142BTV4_9BACT	MamA	x	x	x	x	x	x	x	x	x	x	x	x	x							13	1.00
A0A142BTX3_9BACT	MamA																				0	0.00
A0A142BU17_9BACT	MamA														x						0	0.00
A0A142BU53_9BACT	MamA	x																	x		1	0.08
A0A165G2S6_9BACT	MamA																				0	0.00
C4RAG5_9PROT	MamA													x							1	0.08
C4XPQ7_DESMR	MamA				x		x			x	x	x	x	x	x						7	0.54
C5JBN5_9BACT	MamA			x												x					1	0.08
F5BZ68_9PROT	MamA																				0	0.00
F5BZ69_9PROT	MamA																				0	0.00
F5BZ70_9PROT	MamA																				0	0.00
F5BZ71_9PROT	MamA																				0	0.00
F5BZ72_9PROT	MamA	x																			1	0.08
F5BZ73_9PROT	MamA																				0	0.00
F5BZ74_9PROT	MamA																				0	0.00

		**Eukaryotes**													**A**	**Magnetotactic bacteria**						
**UniProt Accession**	**Protein name**	**1**	**2**	**3**	**4**	**5**	**6**	**7**	**8**	**9**	**10**	**11**	**12**	**13**	**14**	**15**	**16**	**17**	**18**	**19**	**Count**	**% Match**
F5BZ75_9PROT	MamA																				0	0.00
F5BZ76_9PROT	MamA		x						x	x	x	x	x								6	0.46
F5BZ77_9PROT	MamA																				0	0.00
F5BZ78_9PROT	MamA																				0	0.00
G8IQT2_9DELT	MamA				x						x										2	0.15
G9HWD2_9PROT	MamA																				0	0.00
K7Y0D2_9GAMM	MamA						x							x							2	0.15
K7YYZ8_9DELT	MamA				x																1	0.08
L0R455_9DELT	MamA																				0	0.00
L0R598_9DELT	MamA														x						0	0.00
M1RG67_9BACT	MamA								x												1	0.08
Q93DY9_9PROT	MamA																				0	0.00
R6XLS2_9FIRM	MamA																				0	0.00
V6F5J2_9PROT	MamA																				0	0.00
W0LN53_9PROT	MamA	x					x	x				x	x	x							6	0.46
W5S6U7_9PROT	MamA																				0	0.00
W5S6Y4_9PROT	MamA																				0	0.00
W5S7U7_9PROT	MamA																				0	0.00
W6K883_9PROT	MamA						x		x		x	x	x								5	0.38
G8IQT3_9DELT	MamA*																				0	0.00
A0A088F8K0_9BACT	MamB																				0	0.00
A0A0F3GSP9_9BACT	MamB																				0	0.00
A0A0M9ECL1_9DELT	MamB																x				0	0.00
A0A0U5FSV4_9PROT	MamB																				0	0.00
A0A0U5MCU9_9PROT	MamB		x	x			x	x	x	x	x	x	x	x					x	x	10	0.77
A0A0U5MCV0_9PROT	MamB																				0	0.00
A0A109CWW4_9BACT	MamB																				0	0.00
A0A142BTV3_9BACT	MamB			x																	1	0.08
A0A142BTX1_9BACT	MamB																				0	0.00
A0A142BU15_9BACT	MamB															x					0	0.00
A0A142BU51_9BACT	MamB																				0	0.00
A0A1C3RH60_9RHIZ	MamB																				0	0.00
A0A1S7LFA4_9BACT	MamB			x					x	x			x					x			4	0.31
C4RAG2_9PROT	MamB		x																		1	0.08
C4XPP3_DESMR	MamB									x											1	0.08

		**Eukaryotes**													**A**	**Magnetotactic bacteria**						
**UniProt Accession**	**Protein name**	**1**	**2**	**3**	**4**	**5**	**6**	**7**	**8**	**9**	**10**	**11**	**12**	**13**	**14**	**15**	**16**	**17**	**18**	**19**	**Count**	**% Match**
C4XPQ2_DESMR	MamB																				0	0.00
C5JAK7_9BACT	MamB										x										1	0.08
C5JBN1_9BACT	MamB	x			x	x															3	0.23
F5BZ79_9PROT	MamB																				0	0.00
F5BZ80_9PROT	MamB																				0	0.00
F5BZ81_9PROT	MamB																				0	0.00
F5BZ82_9PROT	MamB																				0	0.00
F5BZ83_9PROT	MamB																				0	0.00
F5BZ84_9PROT	MamB																				0	0.00
F5BZ85_9PROT	MamB																				0	0.00
G8IQT4_9DELT	MamB																				0	0.00
G9HWD5_9PROT	MamB																				0	0.00
K7Y5Z5_9DELT	MamB																				0	0.00
K7YZ09_9GAMM	MamB																				0	0.00
L0R3X8_9DELT	MamB																				0	0.00
L0R579_9DELT	MamB																				0	0.00
L0R6P9_9DELT	MamB																				0	0.00
M1RVG4_9BACT	MamB																				0	0.00
Q6NE50_9PROT	MamB																				0	0.00
Q93DY6_9PROT	MamB																				0	0.00
U5IHX2_9DELT	MamB	x										x									2	0.15
V6F510_9PROT	MamB																				0	0.00
W0LMV4_9PROT	MamB							x			x		x	x							4	0.31
W5S6N1_9PROT	MamB																				0	0.00
W5S6Z3_9PROT	MamB																				0	0.00
W5S7U4_9PROT	MamB																				0	0.00
W6KHH6_9PROT	MamB																				0	0.00
A0A0F2J6A4_9BACT	MamB-1														x						0	0.00
A0A0F2J569_9BACT	MamB-2																				0	0.00
G8IQT7_9DELT	MamB*																x				0	0.00
A0A0C2YPV0_MAGMG	MamC																				0	0.00
A0A0U5I168_9PROT	MamC																		x	x	0	0.00
A0A1S7LCW8_9BACT	MamC																	x			0	0.00
Q6NE72_9PROT	MamC																				0	0.00
Q93DY1_9PROT	MamC																				0	0.00

		Eukaryotes													A	Magnetotactic bacteria						
**UniProt Accession**	**Protein name**	**1**	**2**	**3**	**4**	**5**	**6**	**7**	**8**	**9**	**10**	**11**	**12**	**13**	**14**	**15**	**16**	**17**	**18**	**19**	**Count**	**% Match**
V6F534_9PROT	MamC																				0	0.00
W0LJ34_9PROT	MamC																				0	0.00
W6KA44_9PROT	MamC																				0	0.00
A0A0C2UV27_MAGMG	MamD																				0	0.00
A0A0C2UVB2_MAGMG	MamD																		x	x	0	0.00
A0A0U5MJD9_9PROT	MamD																				0	0.00
C5JBM4_9BACT	MamD																				0	0.00
Q6NE73_9PROT	MamD																				0	0.00
Q93DY2_9PROT	MamD																				0	0.00
V6F2D1_9PROT	MamD																				0	0.00
W0LJE9_9PROT	MamD																	x			0	0.00
A0A0U5MIF3_9PROT	MamD-like																				0	0.00
A0A1C3RFM9_9RHIZ	MamD-like																				0	0.00
V6F244_9PROT	MamD-like																		x		0	0.00
K7Y606_9GAMM	MamE																				0	0.00
A0A088FCI2_9BACT	MamE															x					0	0.00
A0A0C2V5Q0_MAGMG	MamE																			x	0	0.00
A0A0C2YZG2_MAGMG	MamE																			x	0	0.00
A0A0F0CIU9_9BACT	MamE																				0	0.00
A0A0F2J699_9BACT	MamE																				0	0.00
A0A0F3GNZ2_9BACT	MamE												x								1	0.08
A0A0F3GW16_9BACT	MamE		x																		1	0.08
A0A0U5MEB8_9PROT	MamE			x															x		1	0.08
A0A0U5MHL9_9PROT	MamE																		x		0	0.00
A0A109CX29_9BACT	MamE																				0	0.00
A0A142BTV6_9BACT	MamE																				0	0.00
A0A142BTX5_9BACT	MamE			x																	1	0.08
A0A142BU19_9BACT	MamE																				0	0.00
A0A142BU55_9BACT	MamE																				0	0.00
A0A1S7LDU1_9BACT	MamE			x		x											x	x			2	0.15
A0A286TXT3_9BACT	MamE	x			x	x		x			x			x		x					6	0.46
C4RAH3_9PROT	MamE			x										x							2	0.15
C5JAJ2_9BACT	MamE			x			x		x	x	x	x	x	x							8	0.62
C5JBP1_9BACT	MamE			x																	1	0.08
L0R6S4_9DELT	MamE																				0	0.00

								<u>**Eukaryotes**</u>							<u>**A**</u>		**Magnetotactic** <u>**bacteria**</u>					
**UniProt Accession**	**Protein name**	**1**	**2**	**3**	**4**	**5**	**6**	**7**	**8**	**9**	**10**	**11**	**12**	**13**	**14**	**15**	**16**	**17**	**18**	**19**	**Count**	**% Match**
M1RA60_9BACT	MamE					x		x	x	x											4	0.31
M1RMJ6_9BACT	MamE																				0	0.00
Q6NE61_9PROT	MamE																				0	0.00
V6F2B6_9PROT	MamE																				0	0.00
W6K5N2_9PROT	MamE																				0	0.00
A0A0M9E3G8_9DELT	MamE-Cter		x	x		x											x	x			3	0.23
A0A0M9ED85_9DELT	MamE-Cter	x		x	x		x		x	x	x		x	x			x				9	0.69
U5IIP0_9DELT	MamE-Cter	x																			1	0.08
A0A0M9E426_9DELT	MamE-Nter																x				0	0.00
A0A0M9EDB3_9DELT	MamE-Nter																x				0	0.00
A0A0N0D6D8_9DELT	MamE-Nter																x				0	0.00
A0A023UKD7_9GAMM	MamE'			x																	1	0.08
G8IQT9_9DELT	MamE*																				0	0.00
M1QLL0_9BACT	MamEO	x			x																2	0.15
U5IGM4_9DELT	MamEO-Cter			x																	1	0.08
U5IHV7_9DELT	MamEO-Nter							x						x			x				2	0.15
A0A0C2U5F8_MAGMG	MamF																		x	x	0	0.00
A0A0U5I0K8_9PROT	MamF																				0	0.00
A0A0U5MFD3_9PROT	MamF																		x		0	0.00
A0A0U5MHZ9_9PROT	MamF												x						x	x	1	0.08
A0A1C3RFM2_9RHIZ	MamF																				0	0.00
A0A1C3RKE7_9RHIZ	MamF																				0	0.00
A0A1S7LFC6_9BACT	MamF																x	x			0	0.00
A0A1S7LFJ0_9BACT	MamF																	x			0	0.00
C5JAI9_9BACT	MamF																				0	0.00
C5JBM6_9BACT	MamF																				0	0.00
Q6NE74_9PROT	MamF																				0	0.00
V6F254_9PROT	MamF																				0	0.00
W0LN58_9PROT	MamF																				0	0.00
C5JBM7_9BACT	MamG-like																			x	0	0.00
A0A0U5N344_9PROT	MamH	x	x					x											x	x	3	0.23
A0A1C3RFR4_9RHIZ	MamH		x	x	x	x	x	x	x	x	x	x	x								11	0.85
A0A1S7LFY5_9BACT	MamH							x	x	x		x						x			4	0.31
C4RAF1_9PROT	MamH	x			x		x	x	x	x	x	x			x						8	0.62
C5JAJ0_9BACT	MamH					x		x	x					x	x	x					4	0.31

		**Eukaryotes**													**A**	**Magnetotactic bacteria**						
**UniProt Accession**	**Protein name**	**1**	**2**	**3**	**4**	**5**	**6**	**7**	**8**	**9**	**10**	**11**	**12**	**13**	**14**	**15**	**16**	**17**	**18**	**19**	**Count**	**% Match**
Q3BKB5_9PROT	MamH																				0	0.00
V6F5J8_9PROT	MamH			x									x								2	0.15
W0LN63_9PROT	MamH	x		x	x	x			x		x	x	x	x							9	0.69
W6K875_9PROT	MamH								x		x			x		x					3	0.23
G8IQU6_9DELT	MamI															x		x			0	0.00
L0R543_9DELT	MamI																				0	0.00
A0A0N0D744_9DELT	MamI-2																x		x	x	0	0.00
M1RA55_9BACT	MamI-2																				0	0.00
U5IIN8_9DELT	MamI-2																				0	0.00
A0A0M9E825_9DELT	MamI-3																x				0	0.00
M1RA63_9BACT	MamI-3																				0	0.00
U5IHX6_9DELT	MamI-3																				0	0.00
A0A0C2YL13_MAGMG	MamJ																				0	0.00
A0A0C2Z0G1_MAGMG	MamJ																		x	x	0	0.00
A0A0U5MF51_9PROT	MamJ																		x		0	0.00
Q3BKB2_9PROT	MamJ																				0	0.00
W5SC56_9PROT	MamJ																				0	0.00
A0A088F9M4_9BACT	MamK	x		x	x			x	x				x	x	x	x					7	0.54
A0A0F2J6W9_9BACT	MamK																				0	0.00
A0A0F3GM49_9BACT	MamK			x						x		x		x							4	0.31
A0A0F3H0I0_9BACT	MamK																				0	0.00
A0A0U5MI56_9PROT	MamK														x				x	x	0	0.00
A0A109CWV6_9BACT	MamK														x						0	0.00
A0A142BTU5_9BACT	MamK		x	x																	2	0.15
A0A142BTW3_9BACT	MamK				x																1	0.08
A0A142BU06_9BACT	MamK																				0	0.00
A0A142BU42_9BACT	MamK					x	x		x		x	x		x							6	0.46
A0A1C3RFN3_9RHIZ	MamK	x	x	x	x		x			x	x				x						7	0.54
A0A1S7LCS9_9BACT	MamK									x								x			1	0.08
C4RAH1_9PROT	MamK																				0	0.00
C5JAJ8_9BACT	MamK			x		x	x		x		x			x							6	0.46
C5JBP0_9BACT	MamK					x															1	0.08
F5BZ86_9PROT	MamK							x													1	0.08
F5BZ87_9PROT	MamK						x		x	x	x	x		x							6	0.46
F5BZ88_9PROT	MamK																				0	0.00

								<u>**Eukaryotes**</u>							<u>**A**</u>			<u>**Magnetotactic bacteria**</u>				
**UniProt Accession**	**Protein name**	**1**	**2**	**3**	**4**	**5**	**6**	**7**	**8**	**9**	**10**	**11**	**12**	**13**	**14**	**15**	**16**	**17**	**18**	**19**	**Count**	**% Match**
F5BZ89_9PROT	MamK																				0	0.00
F5BZ90_9PROT	MamK																				0	0.00
F5BZ91_9PROT	MamK																				0	0.00
F5BZ92_9PROT	MamK																				0	0.00
F5BZ93_9PROT	MamK																				0	0.00
G8IQU0_9DELT	MamK			x																	1	0.08
K7XCG1_9BACT	MamK																				0	0.00
K7Y0C6_9DELT	MamK								x												1	0.08
L0R478_9DELT	MamK																				0	0.00
M1RA72_9BACT	MamK	x	x							x		x	x	x							6	0.46
Q6NE59_9PROT	MamK																				0	0.00
U5IIP4_9DELT	MamK													x							1	0.08
V6F2H8_9PROT	MamK																				0	0.00
W0LJ11_9PROT	MamK																				0	0.00
W5S6M6_9PROT	MamK																				0	0.00
W5S6V4_9PROT	MamK	x	x										x								3	0.23
W5SC65_9PROT	MamK																				0	0.00
A0A0M9E5E5_9DELT	MamK-1																x				0	0.00
A0A0M9E4Z8_9DELT	MamK-2		x		x		x	x	x			x	x	x	x		x				8	0.62
C4RAF3_9PROT	MamK-II							x													1	0.08
K7Y7I3_9GAMM	MamK1	x	x	x	x	x		x													6	0.46
K7Y0D5_9GAMM	MamK2	x																			1	0.08
G8IQU5_9DELT	MamL																x				0	0.00
L0R6R0_9DELT	MamL																				0	0.00
Q6NE58_9PROT	MamL																		x	x	0	0.00
A0A088FCF0_9BACT	MamM															x					0	0.00
A0A0F3GS95_9BACT	MamM																				0	0.00
A0A0N0V1V6_9DELT	MamM			x		x											x				2	0.15
A0A0U5I177_9PROT	MamM																		x	x	0	0.00
A0A109CXI5_9BACT	MamM																				0	0.00
A0A142BTV0_9BACT	MamM																				0	0.00
A0A142BTW8_9BACT	MamM																				0	0.00
A0A142BU12_9BACT	MamM																				0	0.00
A0A142BU48_9BACT	MamM																				0	0.00
A0A1C3RFQ1_9RHIZ	MamM																				0	0.00

		Eukaryotes													A		Magnetotactic bacteria					
**UniProt Accession**	**Protein name**	**1**	**2**	**3**	**4**	**5**	**6**	**7**	**8**	**9**	**10**	**11**	**12**	**13**	**14**	**15**	**16**	**17**	**18**	**19**	**Count**	**% Match**
A0A1C3RFW7_9RHIZ	MamM																				0	0.00
A0A1S7LE79_9BACT	MamM																	x			0	0.00
A0A221SNY2_9PROT	MamM																				0	0.00
C4RAG9_9PROT	MamM																				0	0.00
C5JAK0_9BACT	MamM																				0	0.00
C5JBN8_9BACT	MamM																				0	0.00
F5BZ94_9PROT	MamM																				0	0.00
F5BZ95_9PROT	MamM																				0	0.00
F5BZ96_9PROT	MamM																				0	0.00
F5BZ97_9PROT	MamM																				0	0.00
F5BZ98_9PROT	MamM																				0	0.00
F5BZ99_9PROT	MamM																				0	0.00
F5BZA0_9PROT	MamM																				0	0.00
F5BZA1_9PROT	MamM																				0	0.00
F5BZA2_9PROT	MamM																				0	0.00
G8IQT5_9DELT	MamM																				0	0.00
G8IQT6_9DELT	MamM													x							1	0.08
K7YZ03_9DELT	MamM																				0	0.00
K7YZ14_9GAMM	MamM																				0	0.00
L0R560_9DELT	MamM				x	x	x	x	x	x	x	x	x	x							10	0.77
M1RMK0_9BACT	MamM																				0	0.00
Q6NE57_9PROT	MamM																				0	0.00
U5IGJ7_9DELT	MamM																				0	0.00
V6F235_9PROT	MamM																				0	0.00
W0LP10_9PROT	MamM																				0	0.00
W5S6N4_9PROT	MamM																				0	0.00
W5S6U2_9PROT	MamM																				0	0.00
W5S7V3_9PROT	MamM																				0	0.00
W6K7B7_9PROT	MamM				x																1	0.08
A0A0F2IZI4_9BACT	MamM-2																				0	0.00
A0A0N0V2D5_9DELT	MamMB																x				0	0.00
A0A0C2YZG7_MAGMG	MamN																		x	x	0	0.00
A0A0M9E8B0_9DELT	MamN	x	x		x		x		x		x	x	x	x			x	x			9	0.69
A0A0U5MJE9_9PROT	MamN																				0	0.00
A0A1S7LHQ7_9BACT	MamN																				0	0.00

								Eukaryotes							A			Magnetotactic bacteria				
**UniProt Accession**	**Protein name**	**1**	**2**	**3**	**4**	**5**	**6**	**7**	**8**	**9**	**10**	**11**	**12**	**13**	**14**	**15**	**16**	**17**	**18**	**19**	**Count**	**% Match**
C4RAG8_9PROT	MamN																				0	0.00
C5JAK1_9BACT	MamN							x							x						1	0.08
C5JBN7_9BACT	MamN																				0	0.00
L0R3W4_9DELT	MamN					x				x					x			x			2	0.15
Q6NE56_9PROT	MamN																				0	0.00
V6F2B1_9PROT	MamN																				0	0.00
W6K5N4_9PROT	MamN																				0	0.00
A0A0U5MJR1_9PROT	MamO																	x	x	x	0	0.00
G8IQU1_9DELT	MamO																				0	0.00
G8IQU2_9DELT	MamO*																x				0	0.00
A0A088F9M2_9BACT	MamP															x					0	0.00
A0A0C2Z0Q0_MAGMG	MamP																			x	0	0.00
A0A0F2IZB6_9BACT	MamP																				0	0.00
A0A0F3GSK9_9BACT	MamP																				0	0.00
A0A0U5MHH1_9PROT	MamP																		x		0	0.00
A0A120DCM6_9BACT	MamP																				0	0.00
A0A142BTU9_9BACT	MamP																				0	0.00
A0A142BTW7_9BACT	MamP									x											1	0.08
A0A142BU11_9BACT	MamP																				0	0.00
A0A142BU47_9BACT	MamP																				0	0.00
A0A1S7LCW6_9BACT	MamP					x												x			1	0.08
C4RAG6_9PROT	MamP																				0	0.00
C5JBN4_9BACT	MamP											x									1	0.08
F5BZB3_9PROT	MamP																				0	0.00
F5BZB4_9PROT	MamP																				0	0.00
F5BZB5_9PROT	MamP																				0	0.00
F5BZB6_9PROT	MamP																				0	0.00
F5BZB7_9PROT	MamP																				0	0.00
F5BZB8_9PROT	MamP																				0	0.00
F5BZB9_9PROT	MamP																				0	0.00
F5BZC0_9PROT	MamP																				0	0.00
F5BZC1_9PROT	MamP																				0	0.00
G8IQU8_9DELT	MamP	x					x	x						x							4	0.31
G9HWD4_9PROT	MamP																				0	0.00
G9HWD6_9PROT	MamP		x																		1	0.08

							**Eukaryotes**							**A**			**Magnetotactic bacteria**				
**UniProt Accession**	**Protein name**	**1**	**2**	**3**	**4**	**5**	**6**	**7**	**8**	**9**	**10**	**11**	**12**	**13**	**14**	**15**	**16**	**17**	**18**	**19**	**Count**	**% Match**
G9HWD7_9PROT	MamP																				0	0.00
K7Y602_9DELT	MamP																				0	0.00
K7Y613_9GAMM	MamP																				0	0.00
M1RG72_9BACT	MamP					x															1	0.08
Q93DZ0_9PROT	MamP																				0	0.00
V6F2H3_9PROT	MamP																				0	0.00
W0LJ06_9PROT	MamP								x												1	0.08
W5S6P2_9PROT	MamP																				0	0.00
W5S6V8_9PROT	MamP																				0	0.00
W5S6Y9_9PROT	MamP																				0	0.00
W6K8F2_9PROT	MamP																				0	0.00
G8IQU9_9DELT	MamP*		x	x																	2	0.15
L0R595_9DELT	MamP*																				0	0.00
A0A0C2UG32_MAGMG	MamQ																				0	0.00
A0A0M9EB90_9DELT	MamQ																x				0	0.00
A0A0U5MJL3_9PROT	MamQ																		x	x	0	0.00
A0A1C3RH51_9RHIZ	MamQ																				0	0.00
A0A1S7LDV1_9BACT	MamQ				x									x				x			2	0.15
C4RAG4_9PROT	MamQ																				0	0.00
C5JAK5_9BACT	MamQ																				0	0.00
C5JBN3_9BACT	MamQ																				0	0.00
F5BZC2_9PROT	MamQ																				0	0.00
F5BZC3_9PROT	MamQ										x										1	0.08
F5BZC4_9PROT	MamQ																				0	0.00
F5BZC5_9PROT	MamQ																				0	0.00
F5BZC6_9PROT	MamQ																				0	0.00
F5BZC7_9PROT	MamQ																				0	0.00
F5BZC8_9PROT	MamQ																				0	0.00
F5BZC9_9PROT	MamQ																				0	0.00
F5BZD0_9PROT	MamQ																				0	0.00
G8IQU3_9DELT	MamQ																x				0	0.00
G9HWD3_9PROT	MamQ																				0	0.00
K7YKW0_9DELT	MamQ																				0	0.00
K7YKX0_9GAMM	MamQ																				0	0.00
L0R3V3_9DELT	MamQ																				0	0.00

								Eukaryotes							A			Magnetotactic bacteria				
**UniProt Accession**	**Protein name**	**1**	**2**	**3**	**4**	**5**	**6**	**7**	**8**	**9**	**10**	**11**	**12**	**13**	**14**	**15**	**16**	**17**	**18**	**19**	**Count**	**% Match**
L0R6S9_9DELT	MamQ																				0	0.00
M1QLK4_9BACT	MamQ																				0	0.00
Q93DY8_9PROT	MamQ																				0	0.00
V6F230_9PROT	MamQ																				0	0.00
W0LP06_9PROT	MamQ																				0	0.00
W5S6N8_9PROT	MamQ																				0	0.00
W5S7T9_9PROT	MamQ																				0	0.00
W5S7V9_9PROT	MamQ																				0	0.00
W6K7C1_9PROT	MamQ																				0	0.00
A0A0F3GSP5_9BACT	MamQ-1							x-													1	0.08
A0A0F3GW21_9BACT	MamQ-2																				0	0.00
A0A088F8J3_9BACT	MamQ-I															x					0	0.00
A0A109CXG8_9BACT	MamQ-I																				0	0.00
A0A142BTV7_9BACT	MamQ-I																				0	0.00
A0A142BTX6_9BACT	MamQ-I																				0	0.00
A0A142BU20_9BACT	MamQ-I																				0	0.00
A0A088FCI7_9BACT	MamQ-II															x					0	0.00
A0A0F2J4J9_9BACT	MamQ-II																				0	0.00
A0A109CX26_9BACT	MamQ-II																				0	0.00
A0A142BTV2_9BACT	MamQ-II																				0	0.00
A0A142BTX0_9BACT	MamQ-II																				0	0.00
A0A142BU14_9BACT	MamQ-II																				0	0.00
A0A142BU50_9BACT	MamQ-II																				0	0.00
G8IQU4_9DELT	MamQ*																				0	0.00
A0A0C2UG17_MAGMG	MamS																				0	0.00
A0A0U5MCS7_9PROT	MamS																		x	x	0	0.00
A0A1C3RH65_9RHIZ	MamS																				0	0.00
A0A1S7LFK3_9BACT	MamS									x								x			1	0.08
C4RAG1_9PROT	MamS																				0	0.00
C5JAK8_9BACT	MamS																				0	0.00
C5JBN0_9BACT	MamS																				0	0.00
Q6NE49_9PROT	MamS										x			x							2	0.15
V6F2G8_9PROT	MamS																				0	0.00
W0LIZ9_9PROT	MamS																				0	0.00
W6K8F7_9PROT	MamS																				0	0.00

		<u>**Eukaryotes**</u>													<u>**A**</u>	<u>**Magnetotactic bacteria**</u>						
**UniProt Accession**	**Protein name**	**1**	**2**	**3**	**4**	**5**	**6**	**7**	**8**	**9**	**10**	**11**	**12**	**13**	**14**	**15**	**16**	**17**	**18**	**19**	**Count**	**% Match**
A0A023UJW7_9GAMM	MamT																				0	0.00
A0A0C2V5X2_MAGMG	MamT																		x	x	0	0.00
A0A0M9E1J4_9DELT	MamT																x				0	0.00
A0A0U5MHL5_9PROT	MamT																				0	0.00
A0A1C3RH70_9RHIZ	MamT																				0	0.00
A0A1S7LCY0_9BACT	MamT																	x			0	0.00
C4RAG0_9PROT	MamT														x						0	0.00
C5JBM9_9BACT	MamT									x											1	0.08
F5BZD1_9PROT	MamT																				0	0.00
F5BZD2_9PROT	MamT																				0	0.00
F5BZD3_9PROT	MamT																				0	0.00
F5BZD4_9PROT	MamT																				0	0.00
F5BZD5_9PROT	MamT																				0	0.00
F5BZD6_9PROT	MamT																				0	0.00
F5BZD7_9PROT	MamT																				0	0.00
F5BZD8_9PROT	MamT																				0	0.00
F5BZD9_9PROT	MamT																				0	0.00
G8IQV0_9DELT	MamT			x				x													2	0.15
Q93DY4_9PROT	MamT																				0	0.00
W5S6M1_9PROT	MamT																				0	0.00
W5S701_9PROT	MamT																				0	0.00
W5SC75_9PROT	MamT																				0	0.00
W6K887_9PROT	MamT																				0	0.00
G8IQV1_9DELT	MamT*			x				x			x		x								4	0.31
A0A0C2YUW3_MAGMG	MamU		x	x		x								x		x			x	x	4	0.31
A0A0C2Z0N4_MAGMG	MamU																			x	0	0.00
A0A0U5N358_9PROT	MamU														x				x		0	0.00
Q3BK99_9PROT	MamU	x					x		x	x	x	x	x	x							8	0.62
A0A0C2YL01_MAGMG	MamV											x							x	x	1	0.08
A0A0U5I0Z6_9PROT	MamW									x									x	x	1	0.08
C5JBL8_9BACT	MamW																				0	0.00
Q5D4Z7_9PROT	MamW																				0	0.00
V6F5M3_9PROT	MamW																				0	0.00
A0A0C2V5H3_MAGMG	MamX				x														x	x	1	0.08
A0A0U5MJM3_9PROT	MamX							x													1	0.08

								**Eukaryotes**						**A**			**Magnetotactic bacteria**					
**UniProt Accession**	**Protein name**	**1**	**2**	**3**	**4**	**5**	**6**	**7**	**8**	**9**	**10**	**11**	**12**	**13**	**14**	**15**	**16**	**17**	**18**	**19**	**Count**	**% Match**
A0A1C3RH47_9RHIZ	mamX		x																		1	0.08
A0A1S7LF68_9BACT	MamX						x											x			1	0.08
A4U5C4_9PROT	MamX	x																			1	0.08
V6F2C2_9PROT	MamX																				0	0.00
W0LJD8_9PROT	MamX																				0	0.00
W6K5M8_9PROT	MamX																				0	0.00
A0A0C2UFM5_MAGMG	MamY																		x	x	0	0.00
A0A0U5MHN7_9PROT	MamY				x																1	0.08
C4RAF9_9PROT	MamY																				0	0.00
Q3BK69_9PROT	MamY																x				0	0.00
W6KHI2_9PROT	MamY					x							x								2	0.15
A0A088F8L0_9BACT	Man1															x					0	0.00
A0A125P3G0_9BACT	Man1								x												1	0.08
A0A142BTU4_9BACT	Man1																				0	0.00
A0A142BU05_9BACT	Man1													x							1	0.08
A0A142BU41_9BACT	Man1							x													1	0.08
A0A088F9L3_9BACT	Man4						x		x	x	x	x		x		x					6	0.46
A0A120DCN1_9BACT	Man4							x													1	0.08
A0A142BTV8_9BACT	Man4									x											1	0.08
A0A142BTX7_9BACT	Man4				x					x	x		x								4	0.31
A0A142BU21_9BACT	Man4																				0	0.00
A0A088FCE2_9BACT	Man5				x		x						x	x		x					4	0.31
A0A0F3GS87_9BACT	Man5																				0	0.00
A0A109CWW5_9BACT	Man5																				0	0.00
A0A142BTX8_9BACT	Man5				x			x	x												3	0.23
A0A142BU22_9BACT	Man5																				0	0.00
A0A0F3GSE5_9BACT	Man5-Cter						x		x												2	0.15
A0A088F8P8_9BACT	Man6		x	x			x	x	x	x	x	x	x	x		x					10	0.77
A0A0F3GW11_9BACT	Man6	x	x	x		x		x	x				x								7	0.54
A0A120DCM7_9BACT	Man6		x	x			x	x	x	x	x	x	x				x				9	0.69
A0A142BTX9_9BACT	Man6		x														x				1	0.08
A0A142BU23_9BACT	Man6																				0	0.00
A0A212KIW2_9PROT	MmeA							x													1	0.08
A4TVB2_9PROT	MmeA																		x	x	0	0.00

		**Eukaryotes**													**A**	**Magnetotactic bacteria**						
**UniProt Accession**	**Protein name**	**1**	**2**	**3**	**4**	**5**	**6**	**7**	**8**	**9**	**10**	**11**	**12**	**13**	**14**	**15**	**16**	**17**	**18**	**19**	**Count**	**% Match**
R5Q0D6_9PROT	MmeA																				0	0.00
V6F7D9_9PROT	MmeA																				0	0.00
A0A0C2YPV4_MAGMG	Mms6																			x	0	0.00
A0A0U5MJJ9_9PROT	Mms6																		x		0	0.00
A0A1S7LCS1_9BACT	Mms6																	x			0	0.00
Q3BKD2_9PROT	Mms6																				0	0.00
V6F5K9_9PROT	Mms6																				0	0.00
W6KB84_9PROT	Mms6																				0	0.00
W6KHG2_9PROT	Mms6																				0	0.00
A0A1S7LFH9_9BACT	MmsF																	x			0	0.00
A4U547_9PROT	MmsF																				0	0.00
C4RAE5_9PROT	MmsF																				0	0.00
C4RAF0_9PROT	MmsF																				0	0.00
C5JBM3_9BACT	MmsF																				0	0.00
W0LMX0_9PROT	MmsF																				0	0.00
W6K8E4_9PROT	MmsF																				0	0.00
A0A0F2J2N0_9BACT	Unknown					x				x											2	0.15
A0A0M9E139_9DELT	Unknown	x	x		x	x	x	x	x	x		x	x	x			x				13	0.85
A0A0M9E9F4_9DELT	Unknown																x				0	0.00
A0A0M9ECR7_9DELT	Unknown											x			x		x				1	0.08
U5IGM0_9DELT	Unknown																				0	0.00
W0LJE4_9PROT	Unknown																	x			0	0.00
W0LMZ2_9PROT	Unknown																	x			0	0.00
