# Supplementary material for: Conservation of magnetite biomineralization genes in all domains of life and implications for magnetic sensing
Source: Proc Natl Acad Sci U S A. 2022 Jan 10;119(3):e2108655119. doi: 10.1073/pnas.2108655119 (PMC8784154; doi:10.1073/pnas.2108655119)
Supplement: Supplementary File [file pnas.2108655119.sd03.pdf]

Dataset S3. Summary of numbers of eukaryote and Lokiarchaeota genes with bidirectional BLASTp matches to distant homologs of magnetotactic bacteria (MTB) magnetosome-associated protein sequences. The number of reference sequences (No. Prot.) is listed for each of the 594 named prokaryote proteins (proteins named "Unknown" not shown). The 13 Eukaryote genomes considered are: (1) Panamanian leafcutter ant, *Acromyrmex echinatior*, (2) honeybee, *Apis mellifera*, (3) roundworm, *Caenorhabditis elegans*, (4) fruit fly, *Drosophila melanogaster*, (5) California two-spot octopus, *Octopus bimaculoides*, (6) Little Brown Bat, *Myotis lucifugus*, (7) Chinook salmon, *Oncorhynchus tshawytscha,* (8) human, *Homo sapiens*, (9) naked mole-rat, *Heterocephalus glaber*, (10) mouse, *Mus musculus,* (11) Minke whale, *Balaenoptera acutorostrata scammoni*, (12) zebra finch, *Taeniopygia guttata*, and (13) zebrafish, *Danio rerio*. Column 14 is the asgard clade of Archaea Lokiarchaeota (A), and columns 15-19 are MTB: (15) *Candidatus magnetobacterium casensis*, (16) *Candidatus Magnetomorum* sp. HK-1, (17) *Magnetococcus marinus* MC-1, (18) *Magnetospirillum magneticum* AMB-1, and (19) *Magnetospirillum magnetotacticum* MS1. Taxonomic affinities for MTB are Nitrospirae (n=1), Deltaproteobacteria (n=1), and Alphaproteobacteria (n=3), respectively. See SI Appendix Table S2 for genome accession numbers, references, and additional details. The bidirectional BLASTp matches were filtered for E $<10e^{-3}$ in the eukaryote/Archaea to bacteria protein comparison.

	Eukaryotes													A	Magnetotactic Bacteria					No. Prot.	% match Eukaryotes (of 13 genomes)
Gene	1	2	3	4	5	6	7	8	9	10	11	12	13	14	15	16	17	18	19		
Dam21	0	1	0	0	0	0	0	0	0	0	1	0	0	0	0	0	0	0	0	1	0.15
Dam22	0	0	0	0	0	0	0	0	0	0	0	0	0	0	0	0	0	0	0	1	0.00
Dam28	0	0	1	0	0	0	0	0	0	0	0	0	0	0	0	0	0	0	0	1	0.08
FeoB	1	0	0	1	0	0	1	0	0	0	0	1	1	0	0	1	0	0	0	1	0.38
Mad1	0	1	0	0	0	0	0	1	1	0	0	0	0	0	0	1	0	0	0	3	0.23
Mad2	0	0	0	0	0	0	1	0	0	0	0	0	0	0	1	1	0	0	0	8	0.08
Mad4	0	0	0	0	1	0	0	0	0	0	0	0	0	0	0	1	0	0	0	3	0.08
Mad6	0	0	0	0	0	0	0	0	0	0	1	0	0	1	2	1	1	1	1	3	0.08
Mad7	0	0	0	0	0	0	0	0	0	0	0	0	0	0	0	1	0	0	0	3	0.00
Mad8	0	0	0	0	0	0	0	0	0	0	0	0	0	0	0	1	0	0	0	2	0.00
**Mad9**	**2**	**2**	**2**	**1**	**2**	**2**	**1**	**1**	**1**	**1**	**1**	**1**	**1**	**1**	**2**	**1**	**2**	**2**	**3**	**3**	**1.00**
Mad10	0	0	0	1	1	0	3	0	1	0	1	0	0	0	1	1	0	1	0	9	0.38
Mad11	0	0	0	0	0	0	1	0	0	0	0	0	0	0	0	1	0	0	0	5	0.08
Mad12	0	0	0	0	0	0	0	0	0	0	0	0	0	0	0	1	0	0	0	1	0.00

	Eukaryotes													A	Magnetotactic Bacteria					No. Prot.	% match Eukaryotes (of 13 genomes)
Gene	1	2	3	4	5	6	7	8	9	10	11	12	13	14	15	16	17	18	19		
**Mad17**	**1**	**2**	**2**	**1**	**2**	**2**	**2**	**1**	**2**	**1**	**2**	**2**	**1**	**1**	**1**	**1**	**1**	**1**	**1**	**4**	**1.00**
Mad20	0	0	0	0	0	1	0	0	1	0	1	0	1	0	0	0	0	0	0	3	0.31
Mad21	0	0	0	0	0	0	0	1	0	0	1	0	0	0	0	0	0	0	0	2	0.15
Mad21-1	0	1	0	0	0	1	0	1	1	1	1	0	0	0	0	1	0	0	0	1	0.46
Mad22	1	0	0	1	0	1	1	0	1	0	0	1	1	0	1	1	0	0	0	3	0.54
Mad23	1	1	2	3	1	0	2	0	2	2	2	0	2	1	1	0	0	1	0	7	0.77
Mad23-1	1	1	1	1	1	1	1	0	0	1	0	0	1	0	0	1	0	0	0	1	0.69
Mad23-2	0	0	0	0	0	0	0	0	0	0	0	0	0	0	0	1	0	0	0	1	0.00
Mad23-I	0	1	0	0	0	0	0	0	0	0	0	0	0	0	0	0	0	0	0	1	0.08
Mad23-II	0	0	0	0	0	0	1	0	0	1	0	0	0	0	1	0	0	0	0	1	0.15
Mad24	1	0	1	0	1	1	1	1	0	1	1	0	0	0	1	1	0	1	0	8	0.62
Mad24-2	0	0	0	0	0	1	0	1	1	0	1	1	1	0	0	2	0	0	0	2	0.46
**Mad25**	**2**	**1**	**1**	**1**	**2**	**4**	**1**	**3**	**1**	**1**	**3**	**1**	**2**	**1**	**1**	**1**	**0**	**0**	**0**	**8**	**1.00**
Mad26	0	1	0	0	2	1	1	0	1	1	0	2	1	0	1	0	0	0	0	7	0.62
Mad26-1	0	0	0	0	0	1	0	0	0	0	0	0	0	0	0	1	0	0	0	1	0.08
Mad26-2	0	0	0	1	0	0	1	0	0	0	1	0	0	0	0	1	0	0	0	1	0.23
Mad27	1	0	0	1	1	0	0	0	0	0	0	0	1	1	1	0	0	0	0	1	0.31
Mad27-1	0	0	1	1	1	1	1	1	1	1	1	1	1	0	0	1	1	0	0	1	0.85
Mad27-2	0	1	1	1	1	1	0	1	1	1	1	1	1	0	1	1	0	1	1	1	0.85
Mad28	1	0	0	1	0	1	1	0	1	1	1	0	0	0	1	0	0	0	0	7	0.54
Mad28-1	0	0	1	0	0	0	1	1	0	1	1	0	0	0	0	1	0	0	0	3	0.38
Mad28-2	0	2	0	1	1	0	1	0	0	1	1	1	1	0	1	1	0	0	0	4	0.62
Mad28-3	0	0	0	0	0	0	0	0	0	0	0	0	0	0	0	0	0	0	0	1	0.00
Mad28-5	0	0	0	0	0	0	0	0	1	0	0	0	0	0	0	0	0	0	0	1	0.08
Mad28-6	0	0	0	0	0	0	0	0	1	1	0	0	0	0	0	0	0	0	0	1	0.15
**Mad29**	**2**	**1**	**1**	**1**	**1**	**1**	**1**	**1**	**1**	**1**	**1**	**1**	**2**	**1**	**0**	**1**	**1**	**1**	**1**	**5**	**1.00**

Gene	Eukaryotes													A	Magnetotactic Bacteria					No. Prot.	% match Eukaryotes (of 13 genomes)
	1	2	3	4	5	6	7	8	9	10	11	12	13	14	15	16	17	18	19		
Mad29-1	0	0	0	0	0	1	0	0	1	0	0	1	0	0	0	0	0	0	0	1	0.23
Mad29-2	0	0	0	0	0	0	0	0	0	0	0	0	0	1	1	0	0	0	0	1	0.00
Mad30	0	0	0	0	0	0	1	1	0	0	0	0	0	1	1	1	0	2	2	7	0.15
Mad31	0	0	0	0	0	0	0	0	0	0	0	0	0	0	1	1	0	0	0	7	0.00
**MamA**	**5**	**2**	**2**	**5**	**1**	**6**	**6**	**6**	**4**	**7**	**6**	**6**	**8**	**3**	**3**	**2**	**3**	**2**	**2**	**42**	**1.00**
MamA*	0	0	0	0	0	0	0	0	0	0	0	0	0	0	0	0	0	0	0	1	0.00
**MamB**	**2**	**2**	**3**	**1**	**1**	**1**	**2**	**2**	**3**	**3**	**2**	**3**	**2**	**0**	**1**	**1**	**1**	**1**	**1**	**42**	**1.00**
MamB-1	0	0	0	0	0	0	0	0	0	0	0	0	0	1	0	0	0	0	0	1	0.00
MamB-2	0	0	0	0	0	0	0	0	0	0	0	0	0	0	0	0	0	0	0	1	0.00
MamB*	0	0	0	0	0	0	0	0	0	0	0	0	0	0	0	1	0	0	0	1	0.00
MamC	0	0	0	0	0	0	0	0	0	0	0	0	0	0	0	0	1	1	1	8	0.00
MamD	0	0	0	0	0	0	0	0	0	0	0	0	0	0	0	0	1	1	1	8	0.00
MamD-like	0	0	0	0	0	0	0	0	0	0	0	0	0	0	0	0	0	1	0	3	0.00
**MamE**	**1**	**1**	**6**	**1**	**3**	**1**	**2**	**2**	**2**	**2**	**1**	**2**	**3**	**0**	**2**	**1**	**1**	**2**	**2**	**26**	**1.00**
MamE-Cter	2	1	2	1	1	1	0	1	1	1	0	1	1	0	0	2	1	0	0	3	0.85
MamE-Nter	0	0	0	0	0	0	0	0	0	0	0	0	0	0	0	3	0	0	0	3	0.00
MamE'	0	0	1	0	0	0	0	0	0	0	0	0	0	0	0	0	0	0	0	1	0.08
MamE*	0	0	0	0	0	0	0	0	0	0	0	0	0	0	0	0	0	0	0	1	0.00
MamEO	1	0	0	1	0	0	0	0	0	0	0	0	0	0	0	0	0	0	0	1	0.15
MamEO-Cter	0	0	1	0	0	0	0	0	0	0	0	0	0	0	0	0	0	0	0	1	0.08
MamEO-Nter	0	0	0	0	0	0	1	0	0	0	0	0	1	0	0	1	0	0	0	1	0.15
MamF	0	0	0	0	0	0	0	0	0	0	0	1	0	0	0	1	2	3	2	13	0.08
MamG-like	0	0	0	0	0	0	0	0	0	0	0	0	0	0	0	0	0	0	1	1	0.00
**MamH**	**3**	**2**	**3**	**3**	**3**	**2**	**5**	**6**	**3**	**4**	**4**	**3**	**3**	**2**	**2**	**0**	**1**	**1**	**1**	**9**	**1.00**
MamI	0	0	0	0	0	0	0	0	0	0	0	0	0	0	1	0	1	0	0	2	0.00
MamI-2	0	0	0	0	0	0	0	0	0	0	0	0	0	0	0	1	0	1	1	3	0.00

	Eukaryotes													A	Magnetotactic Bacteria					No. Prot.	% match Eukaryotes (of 13 genomes)
Gene	1	2	3	4	5	6	7	8	9	10	11	12	13	14	15	16	17	18	19		
MamI-3	0	0	0	0	0	0	0	0	0	0	0	0	0	0	0	1	0	0	0	3	0.00
MamJ	0	0	0	0	0	0	0	0	0	0	0	0	0	0	0	0	0	2	1	5	0.00
**MamK**	**4**	**4**	**6**	**3**	**3**	**4**	**2**	**5**	**5**	**4**	**4**	**3**	**7**	**4**	**1**	**0**	**1**	**1**	**1**	**35**	**1.00**
MamK-1	0	0	0	0	0	0	0	0	0	0	0	0	0	0	0	1	0	0	0	1	0.00
MamK-2	0	1	0	1	0	1	1	1	0	0	1	1	1	1	0	1	0	0	0	1	0.62
MamK-II	0	0	0	0	0	0	1	0	0	0	0	0	0	0	0	0	0	0	0	1	0.08
MamK1	1	1	1	1	1	0	1	0	0	0	0	0	0	0	0	0	0	0	0	1	0.46
MamK2	1	0	0	0	0	0	0	0	0	0	0	0	0	0	0	0	0	0	0	1	0.08
MamL	0	0	0	0	0	0	0	0	0	0	0	0	0	0	0	1	0	1	1	3	0.00
MamM	0	0	1	2	2	1	1	1	1	1	1	1	2	0	1	1	1	1	1	39	0.85
MamM-2	0	0	0	0	0	0	0	0	0	0	0	0	0	0	0	0	0	0	0	1	0.00
MamMB	0	0	0	0	0	0	0	0	0	0	0	0	0	0	0	1	0	0	0	1	0.00
**MamN**	**1**	**1**	**0**	**1**	**1**	**1**	**1**	**1**	**1**	**1**	**1**	**1**	**1**	**2**	**0**	**1**	**2**	**1**	**1**	**11**	**0.92**
MamO	0	0	0	0	0	0	0	0	0	0	0	0	0	0	0	0	1	1	1	2	0.00
MamO*	0	0	0	0	0	0	0	0	0	0	0	0	0	0	0	1	0	0	0	1	0.00
MamP	1	1	0	0	2	1	1	1	1	0	1	0	1	0	1	0	1	1	1	36	0.69
MamP*	0	1	1	0	0	0	0	0	0	0	0	0	0	0	0	0	0	0	0	2	0.15
MamQ	0	0	0	1	0	0	0	0	0	1	0	0	1	0	0	2	1	1	1	31	0.23
MamQ-1	0	0	0	0	0	0	1	0	0	0	0	0	0	0	0	0	0	0	0	1	0.08
MamQ-2	0	0	0	0	0	0	0	0	0	0	0	0	0	0	0	0	0	0	0	1	0.00
MamQ-I	0	0	0	0	0	0	0	0	0	0	0	0	0	0	1	0	0	0	0	5	0.00
MamQ-II	0	0	0	0	0	0	0	0	0	0	0	0	0	0	1	0	0	0	0	7	0.00
MamQ*	0	0	0	0	0	0	0	0	0	0	0	0	0	0	0	0	0	0	0	1	0.00
MamS	0	0	0	0	0	0	0	0	1	1	0	0	1	0	0	0	1	1	1	11	0.23
MamT	0	0	1	0	0	0	1	0	1	0	0	0	0	1	0	1	1	1	1	23	0.23
MamT*	0	0	1	0	0	0	1	0	0	1	0	1	0	0	0	0	0	0	0	1	0.31

Gene	Eukaryotes													A	Magnetotactic Bacteria					No. Prot.	% match Eukaryotes (of 13 genomes)
	1	2	3	4	5	6	7	8	9	10	11	12	13	14	15	16	17	18	19		
MamU	1	1	1	0	1	1	0	1	1	1	1	1	2	1	1	0	0	2	2	4	0.85
MamV	0	0	0	0	0	0	0	0	0	0	1	0	0	0	0	0	0	1	1	1	0.08
MamW	0	0	0	0	0	0	0	0	1	0	0	0	0	0	0	0	0	1	1	4	0.08
MamX	1	1	0	1	0	1	1	0	0	0	0	0	0	0	0	0	1	1	1	8	0.38
MamY	0	0	0	1	1	0	0	0	0	0	0	1	0	0	0	1	0	1	1	5	0.23
Man1	0	0	0	0	0	0	1	1	0	0	0	0	1	0	1	0	0	0	0	5	0.23
Man4	0	0	0	1	0	1	1	1	3	2	1	1	1	0	1	0	0	0	0	5	0.69
Man5	0	0	0	2	0	1	1	1	0	0	0	1	1	0	1	0	0	0	0	5	0.46
Man5-Cter	0	0	0	0	0	1	0	1	0	0	0	0	0	0	0	0	0	0	0	1	0.15
**Man6**	**1**	**4**	**3**	**0**	**1**	**2**	**3**	**3**	**2**	**2**	**2**	**3**	**1**	**1**	**1**	**2**	**0**	**0**	**0**	**5**	**0.92**
MmeA	0	0	0	0	0	0	1	0	0	0	0	0	0	0	0	0	0	1	1	4	0.08
Mms6	0	0	0	0	0	0	0	0	0	0	0	0	0	0	0	0	1	1	1	7	0.00
MmsF	0	0	0	0	0	0	0	0	0	0	0	0	0	0	0	0	1	0	0	7	0.00
