# Supplementary material for: Conservation of magnetite biomineralization genes in all domains of life and implications for magnetic sensing
Source: Proc Natl Acad Sci U S A. 2022 Jan 10;119(3):e2108655119. doi: 10.1073/pnas.2108655119 (PMC8784154; doi:10.1073/pnas.2108655119)
Supplement: Supplementary File [file pnas.2108655119.sd05.pdf]

Dataset S5. Functional annotations of zebrafish (*Danio rerio*) and Chinook salmon (*Oncorhynchus tshawytscha*) genes showing distant homology to magnetotactic bacteria biomineralization proteins. The complete repertoire of zebrafish (226) and Chinook salmon genes (181) identified as ‘universally conserved’ in eukaryotes and with matches to zebrafish ZDB-Gene identifiers were functionally annotated in PANTHER, Protein Analysis Through Evolutionary Relationships, applying the built-in Benjamini-Hochberg false discovery rate (FDR P < 0.05) to correct for multiple tests. All categories are listed regardless of significance level. Results are shown for gene ontology (GO) complete categories biological process (BP), molecular function (MF), cellular component (CC), and the reactome pathway (R). Shading is used to depict individual GO category groupings, with sub-category hierarchy (H) indicated using a numeric scale: zero indicates the most specific sub-category, and incrementally higher numbers indicate the next-broader-level within each ontology hierarchy. The number of observed genes (No. Obs.) refers to the number of genes assigned to each GO category, while the number of expected genes (No. Exp.) is modeled by PANTHER using the number of genes in the input file relative to the background contents of the zebrafish genome (No. genes in genome). Fold-enrichment (F-E) is calculated by dividing No. Obs. by No. Exp, based on background genome contents of zebrafish.

				Zebrafish					Chinook salmon				
Cat.	H	GO Complete	No. Genes in Genome	No. Obs.	No. Exp.	F-E	Raw P value	FDR	No. Obs.	No. Exp.	F-E	Raw P value	FDR
BP	0	protein refolding	23	10	0.21	48.01	2.15E-13	6.34E-10	6	0.16	36.45	4.92E-08	1.09E-04
	1	protein folding	125	17	1.13	15.02	1.30E-14	5.77E-11	13	0.89	14.53	2.08E-11	9.21E-08
	0	zinc ion transport	20	8	0.18	44.17	9.91E-11	9.75E-08	7	0.14	48.90	6.42E-10	1.89E-06
	1	transition metal ion transport	75	9	0.68	13.25	6.62E-08	3.91E-05	7	0.54	13.04	1.98E-06	1.94E-03
	0	cellular response to heat	23	9	0.21	43.21	7.56E-12	9.56E-09	5	0.16	30.37	1.48E-06	1.63E-03
	1	response to heat	34	9	0.31	29.23	1.40E-10	1.24E-07	5	0.24	20.55	8.12E-06	5.99E-03
	2	response to temperature stimulus	57	9	0.52	17.44	7.66E-09	4.84E-06	5	0.41	12.26	7.99E-05	3.93E-02
	0	response to zinc ion	13	5	0.12	42.47	4.34E-07	2.26E-04	4	0.09	42.99	5.46E-06	4.40E-03
	0	chaperone cofactor-dependent protein refolding	27	10	0.24	40.90	7.84E-13	1.74E-09	6	0.19	31.05	1.12E-07	1.98E-04
	1	chaperone-mediated protein folding	34	13	0.31	42.22	1.87E-16	1.65E-12	10	0.24	41.09	5.15E-13	4.56E-09
	1	'de novo' posttranslational protein folding	28	10	0.25	39.44	1.06E-12	1.87E-09	6	0.2	29.94	1.35E-07	1.99E-04
	2	'de novo' protein folding	29	10	0.26	38.08	1.41E-12	2.08E-09	6	0.21	28.91	1.62E-07	2.05E-04
	0	mesenchyme morphogenesis	11	4	0.10	40.16	7.98E-06	3.93E-03	3	0.08	38.10	1.21E-04	4.88E-02
	0	cellular response to unfolded protein	31	9	0.28	32.06	6.92E-11	7.66E-08	5	0.22	22.54	5.41E-06	4.79E-03
	1	response to unfolded protein	37	9	0.34	26.86	2.66E-10	2.14E-07	5	0.26	18.88	1.18E-05	8.03E-03
	2	response to topologically incorrect protein	54	9	0.49	18.41	5.01E-09	3.41E-06	5	0.39	12.94	6.29E-05	3.28E-02
	1	cellular response to topologically incorrect protein	48	9	0.43	20.71	2.00E-09	1.47E-06	5	0.34	14.55	3.73E-05	2.36E-02
	0	protein peptidyl-prolyl isomerization	24	4	0.22	18.41	1.09E-04	3.33E-02	4	0.17	23.29	4.42E-05	2.45E-02
MF	0	protein folding chaperone	11	9	0.1	90.35	4.97E-14	2.64E-11	5	0.08	63.51	7.03E-08	3.20E-05

Cat.	H	GO Complete	No. Genes in Genome	Zebrafish					Chinook salmon				
				No. Obs.	No. Exp.	F-E	Raw P value	FDR	No. Obs.	No. Exp.	F-E	Raw P value	FDR
	0	misfolded protein binding	14	11	0.13	86.77	9.53E-17	1.01E-13	6	0.1	59.88	4.23E-09	3.36E-06
	0	zinc ion transmembrane transporter activity	20	8	0.18	44.17	9.91E-11	3.94E-08	7	0.14	48.9	6.42E-10	6.81E-07
	1	divalent inorganic cation transmembrane transporter activity	20	8	0.18	44.17	9.91E-11	4.50E-08	7	0.14	48.90	6.42E-10	1.02E-06
	1	transition metal ion transmembrane transporter activity	32	8	0.29	27.61	2.24E-09	6.47E-07	7	0.23	30.56	1.03E-08	6.58E-06
	0	Hsp90 protein binding	12	3	0.11	27.61	3.00E-04	3.29E-02	4	0.09	46.57	4.20E-06	1.48E-03
	1	heat shock protein binding	41	14	0.37	37.71	4.63E-17	7.37E-14	11	0.29	37.49	8.06E-14	2.56E-10
	0	peptidyl-prolyl cis-trans isomerase activity	41	7	0.37	18.85	2.30E-07	4.89E-05	7	0.29	23.85	4.69E-08	2.49E-05
	1	cis-trans isomerase activity	50	7	0.45	15.46	7.72E-07	1.54E-04	7	0.36	19.56	1.60E-07	6.35E-05
	2	isomerase activity	134	7	1.21	5.77	2.92E-04	3.44E-02	7	0.96	7.30	6.92E-05	2.00E-02
	0	4 iron, 4 sulfur cluster binding	36	5	0.33	15.34	3.21E-05	4.86E-03	4	0.26	15.52	1.85E-04	4.52E-02
	0	insulin-like growth factor binding	32	4	0.29	13.80	2.97E-04	3.38E-02	4	0.23	17.46	1.22E-04	3.23E-02
	0	unfolded protein binding	96	12	0.87	13.80	2.67E-10	8.50E-08	7	0.69	10.19	9.09E-06	2.89E-03
CC R	0	actin-based cell projection	29	5	0.26	19.04	0.000	0.017	5	0.21	24.09	0.000	0.006
	0	Zinc efflux and compartmentalization by the SLC30 family	4	4	0.04	> 100	4.30E-07	1.68E-04	2	0.03	69.86	7.39E-04	4.44E-02
	0	c-src mediated regulation of Cx43 function and closure of gap junctions	4	2	0.04	55.22	1.17E-03	3.67E-02	2	0.03	69.86	7.39E-04	4.62E-02
	1	Regulation of gap junction activity	4	2	0.04	55.22	1.17E-03	3.74E-02	2	0.03	69.86	7.39E-04	4.81E-02
	2	Gap junction trafficking and regulation	20	4	0.18	22.09	5.83E-05	3.50E-03	3	0.14	20.96	5.63E-04	4.00E-02
	0	Phosphorylation of the APC/C	18	5	0.16	30.68	1.64E-06	3.21E-04	4	0.13	31.05	1.63E-05	3.19E-03
	0	HSP90 chaperone cycle for steroid hormone receptors (SHR)	18	5	0.16	30.68	1.64E-06	2.85E-04	4	0.13	31.05	1.63E-05	2.83E-03
	1	Cellular responses to stress	292	12	2.64	4.54	2.14E-05	1.59E-03	9	2.09	4.31	3.18E-04	2.76E-02
	0	Inactivation of APC/C via direct inhibition of the APC/C complex	19	5	0.17	29.06	2.06E-06	3.22E-04	4	0.14	29.41	1.97E-05	3.07E-03
	1	Inhibition of the proteolytic activity of APC/C required for the onset of anaphase by mitotic spindle checkpoint components	19	5	0.17	29.06	2.06E-06	2.93E-04	4	0.14	29.41	1.97E-05	2.79E-03
	2	Regulation of APC/C activators between G1/S and early anaphase	30	5	0.27	18.41	1.45E-05	1.13E-03	4	0.21	18.63	9.68E-05	1.01E-02
	0	Conversion from APC/C:Cdc20 to APC/C:Cdh1 in late anaphase	19	5	0.17	29.06	2.06E-06	2.68E-04	4	0.14	29.41	1.97E-05	2.56E-03

Cat.	H	GO Complete	No. Genes in Genome	Zebrafish No. Obs.	Zebrafish No. Exp.	Zebrafish F-E	Zebrafish Raw P value	Zebrafish FDR	Chinook salmon No. Obs.	Chinook salmon No. Exp.	Chinook salmon F-E	Chinook salmon Raw P value	Chinook salmon FDR
	0	APC/C:Cdc20 mediated degradation of Cyclin B	22	5	0.2	25.1	3.83E-06	4.27E-04	4	0.16	25.40	3.26E-05	3.92E-03
	0	MET activates PTK2 signaling	37	8	0.34	23.88	6.03E-09	9.42E-06	6	0.26	22.66	5.81E-07	4.54E-04
	1	MET promotes cell motility	54	8	0.49	16.36	8.29E-08	4.32E-05	6	0.39	15.52	4.32E-06	1.69E-03
	2	Signaling by MET	98	9	0.89	10.14	5.40E-07	1.69E-04	6	0.7	8.55	1.01E-04	9.87E-03
	3	Signaling by Receptor Tyrosine Kinases	449	16	4.07	3.94	5.27E-06	4.84E-04	14	3.21	4.36	6.21E-06	1.62E-03
	4	Signal Transduction	2196	36	19.89	1.81	5.63E-04	2.15E-02	36	15.72	2.29	3.60E-06	1.88E-03
	0	APC-Cdc20 mediated degradation of Nek2A	24	5	0.22	23.01	5.55E-06	4.82E-04	4	0.17	23.29	4.42E-05	4.93E-03
	0	Laminin interactions	34	7	0.31	22.74	7.42E-08	5.79E-05	6	0.24	24.66	3.72E-07	5.81E-04
	0	EPHB-mediated forward signaling	63	7	0.57	12.27	3.17E-06	3.81E-04	5	0.45	11.09	1.24E-04	1.14E-02
	1	EPH-Ephrin signaling	96	8	0.87	9.2	4.57E-06	4.47E-04	5	0.69	7.28	7.83E-04	4.53E-02
	0	RHO GTPase Effectors	269	12	2.44	4.93	9.80E-06	8.06E-04	11	1.93	5.71	5.62E-06	1.75E-03
	1	Signaling by Rho GTPases	418	13	3.79	3.43	1.53E-04	7.02E-03	13	2.99	4.35	1.38E-05	3.08E-03
