# Supplementary material for: Conservation of magnetite biomineralization genes in all domains of life and implications for magnetic sensing
Source: Proc Natl Acad Sci U S A. 2022 Jan 10;119(3):e2108655119. doi: 10.1073/pnas.2108655119 (PMC8784154; doi:10.1073/pnas.2108655119)
Supplement: Supplementary File [file pnas.2108655119.sd06.pdf]

Dataset 6. The complete repertoire (homologs and paralogs) of zebrafish (*Danio rerio*) and Chinook salmon (*Oncorhynchus tshawytscha*) genes encoding 11 'universally conserved' uMGH proteins. The Chinook salmon genes were matched to zebrafish orthologs in order to identify Zebrafish Information Network gene codes (see Methods). The Protein ANalysis THrough Evolutionary Relationships Protein Classes are based on the 2020_04 release of "ReferenceProteome" dataset. The salmon accessions are available from the National Center for Bioinformatic Information (Accession GCF_002872995.1), while zebrafish accessions are available from ENSEMBL genome version GRCz11, file 'Danio_rerio.GRCz11.pep.all.fa.

uMGH Category	ZFIN gene code	PANTHER protein class	Accession	Fish
MamA	ZDB-GENE-040426-928	-	XP_024228601.1	Chinook salmon
MamK	ZDB-GENE-070410-58	-	XP_024228727.1	Chinook salmon
MamN	ZDB-GENE-070424-71	secondary carrier transporter	XP_024228764.1	Chinook salmon
MamK	ZDB-GENE-041121-7	actin and actin related protein	XP_024229240.1	Chinook salmon
MamA	ZDB-GENE-030131-2527	-	XP_024229660.1	Chinook salmon
MamA	ZDB-GENE-070705-219	-	XP_024229808.1	Chinook salmon
Man6	ZDB-GENE-021226-1	extracellular matrix protein	XP_024229830.1	Chinook salmon
MamA	ZDB-GENE-030616-630	chaperone	XP_024229852.1	Chinook salmon
MamA	ZDB-GENE-120727-22	protein modifying enzyme	XP_024230054.1	Chinook salmon
MamA	ZDB-GENE-070209-29	protein-binding activity modulator	XP_024230099.1	Chinook salmon
MamE	ZDB-GENE-030519-2	-	XP_024230496.1	Chinook salmon
MamE	ZDB-GENE-030616-572	scaffold/adaptor protein	XP_024230731.1	Chinook salmon
MamK	ZDB-GENE-040426-2877	actin and actin related protein	XP_024230997.1	Chinook salmon
MamH	ZDB-GENE-040718-380	secondary carrier transporter	XP_024231796.1	Chinook salmon
MamB	ZDB-GENE-041210-158	-	XP_024232636.1	Chinook salmon
Man6	ZDB-GENE-021226-1	extracellular matrix protein	XP_024232688.1	Chinook salmon
MamN	ZDB-GENE-070424-71	secondary carrier transporter	XP_024232770.1	Chinook salmon
MamA	ZDB-GENE-070705-219	-	XP_024232821.1	Chinook salmon
MamA	ZDB-GENE-040426-928	-	XP_024232920.1	Chinook salmon
Man6	ZDB-GENE-030131-7905	-	XP_024233250.1	Chinook salmon
MamB	ZDB-GENE-040426-1840	-	XP_024233478.1	Chinook salmon

uMGH Category	ZFIN gene code	PANTHER protein class	Accession	Fish
MamA	ZDB-GENE-040426-694	RNA splicing factor	XP_024233544.1	Chinook salmon
Mad25/Man6	ZDB-GENE-060118-1	extracellular matrix protein	XP_024233678.1	Chinook salmon
MamA	ZDB-GENE-080722-25	-	XP_024233785.1	Chinook salmon
MamH	ZDB-GENE-041210-308	-	XP_024234123.1	Chinook salmon
MamK	ZDB-GENE-040625-35	actin and actin related protein	XP_024234289.1	Chinook salmon
MamK	ZDB-GENE-040520-4	actin and actin related protein	XP_024234338.1	Chinook salmon
MamA	ZDB-GENE-030131-9443	-	XP_024234369.1	Chinook salmon
MamA	ZDB-GENE-060126-2	-	XP_024234753.1	Chinook salmon
MamA	ZDB-GENE-050208-116	chaperone	XP_024235293.1	Chinook salmon
MamA	ZDB-GENE-030131-514	chaperone	XP_024235350.1	Chinook salmon
MamA	ZDB-GENE-041210-299	protein modifying enzyme	XP_024235642.1	Chinook salmon
MamK	ZDB-GENE-041111-251	GTPase-activating protein	XP_024235815.1	Chinook salmon
MamA	ZDB-GENE-061221-2	-	XP_024235847.1	Chinook salmon
MamK	ZDB-GENE-040520-4	actin and actin related protein	XP_024235880.1	Chinook salmon
Mad25	ZDB-GENE-121214-181	membrane traffic protein	XP_024236113.1	Chinook salmon
MamK	ZDB-GENE-031001-11	-	XP_024236220.1	Chinook salmon
MamA	ZDB-GENE-110203-5	-	XP_024236246.1	Chinook salmon
Man6	ZDB-GENE-030131-3404	-	XP_024236304.1	Chinook salmon
Mad17	ZDB-GENE-030131-8767	-	XP_024236389.1	Chinook salmon
MamK	ZDB-GENE-040426-2695	actin and actin related protein	XP_024237762.1	Chinook salmon
MamA	ZDB-GENE-040704-72	-	XP_024237889.1	Chinook salmon
MamE	ZDB-GENE-091118-27	cytoskeletal protein	XP_024237920.1	Chinook salmon
MamE	ZDB-GENE-091118-27	cytoskeletal protein	XP_024237967.1	Chinook salmon
MamE	ZDB-GENE-070925-2	tight junction	XP_024238050.1	Chinook salmon
MamE	ZDB-GENE-040426-2544	cell junction protein	XP_024238242.1	Chinook salmon
MamA	ZDB-GENE-030131-8303	ubiquitin-protein ligase	XP_024238318.1	Chinook salmon
MamE	ZDB-GENE-030131-6959	guanyl-nucleotide exchange factor	XP_024238901.1	Chinook salmon

uMGH Category	ZFIN gene code	PANTHER protein class	Accession	Fish
MamB	ZDB-GENE-040724-254	transporter	XP_024239008.1	Chinook salmon
MamE	ZDB-GENE-080520-2	-	XP_024239312.1	Chinook salmon
MamA	ZDB-GENE-030313-1	-	XP_024239706.1	Chinook salmon
Mad17	ZDB-GENE-030131-2209	-	XP_024240635.1	Chinook salmon
MamK	ZDB-GENE-040426-1221	-	XP_024241239.1	Chinook salmon
MamA	ZDB-GENE-121214-233	-	XP_024242067.1	Chinook salmon
MamA	ZDB-GENE-121214-233	-	XP_024242233.1	Chinook salmon
MamA	ZDB-GENE-121214-248	-	XP_024242234.1	Chinook salmon
MamE	ZDB-GENE-080219-7	serine protease	XP_024242286.1	Chinook salmon
MamE	ZDB-GENE-090313-191	cytoskeletal protein	XP_024242489.1	Chinook salmon
MamA	ZDB-GENE-030131-2963	ubiquitin-protein ligase	XP_024242638.1	Chinook salmon
Mad29	ZDB-GENE-051023-10	-	XP_024242659.1	Chinook salmon
MamE	ZDB-GENE-121019-1	-	XP_024242820.1	Chinook salmon
MamE	ZDB-GENE-080219-7	serine protease	XP_024243445.1	Chinook salmon
MamA	ZDB-GENE-121214-233	-	XP_024243502.1	Chinook salmon
MamA	ZDB-GENE-060130-180	-	XP_024243879.1	Chinook salmon
MamK	ZDB-GENE-040520-4	actin and actin related protein	XP_024243910.1	Chinook salmon
MamA	ZDB-GENE-081105-56	chromatin/chromatin-binding, or -regulatory protein	XP_024244156.1	Chinook salmon
MamK	ZDB-GENE-030131-1229	actin and actin related protein	XP_024244208.1	Chinook salmon
MamN/MamE	ZDB-GENE-040724-161	-	XP_024244227.1	Chinook salmon
MamA	ZDB-GENE-051113-132	ubiquitin-protein ligase	XP_024244419.1	Chinook salmon
MamA	ZDB-GENE-070912-148	-	XP_024244429.1	Chinook salmon
MamK	ZDB-GENE-070424-10	actin and actin related protein	XP_024244528.1	Chinook salmon
MamA	ZDB-GENE-130530-956	-	XP_024244642.1	Chinook salmon
MamA	ZDB-GENE-031010-34	-	XP_024244693.1	Chinook salmon
MamA	ZDB-GENE-081105-56	chromatin/chromatin-binding, or -regulatory protein	XP_024244916.1	Chinook salmon
MamE	ZDB-GENE-031222-1	-	XP_024245149.1	Chinook salmon

uMGH Category	ZFIN gene code	PANTHER protein class	Accession	Fish
MamA	ZDB-GENE-990706-2	ubiquitin-protein ligase	XP_024245781.1	Chinook salmon
MamE	ZDB-GENE-081031-78	-	XP_024245876.1	Chinook salmon
MamA	ZDB-GENE-040426-2483	chaperone	XP_024245916.1	Chinook salmon
MamA	ZDB-GENE-080208-7	nucleic acid metabolism protein	XP_024246188.1	Chinook salmon
MamA	ZDB-GENE-030131-5395	-	XP_024246210.1	Chinook salmon
MamA	ZDB-GENE-040426-1169	-	XP_024246655.1	Chinook salmon
MamA	ZDB-GENE-041114-56	-	XP_024246929.1	Chinook salmon
Mad25	ZDB-GENE-100712-2	non-receptor serine/threonine protein kinase	XP_024247085.1	Chinook salmon
MamB	ZDB-GENE-030131-5556	non-receptor serine/threonine protein kinase	XP_024247301.1	Chinook salmon
Man6	ZDB-GENE-021226-3	extracellular matrix protein	XP_024247452.1	Chinook salmon
Man6	ZDB-GENE-110222-3	extracellular matrix protein	XP_024247454.1	Chinook salmon
MamE	ZDB-GENE-050302-100	-	XP_024247912.1	Chinook salmon
Man6	ZDB-GENE-030131-9823	extracellular matrix protein	XP_024248018.1	Chinook salmon
MamA	ZDB-GENE-031010-34	-	XP_024248039.1	Chinook salmon
MamE	ZDB-GENE-030616-572	scaffold/adaptor protein	XP_024248149.1	Chinook salmon
MamA	ZDB-GENE-070209-29	protein-binding activity modulator	XP_024248357.1	Chinook salmon
MamA	ZDB-GENE-120727-22	protein modifying enzyme	XP_024248389.1	Chinook salmon
MamA	ZDB-GENE-091204-117	-	XP_024248834.1	Chinook salmon
MamK	ZDB-GENE-990415-92	-	XP_024249523.1	Chinook salmon
MamA	ZDB-GENE-040426-1169	-	XP_024249903.1	Chinook salmon
MamA	ZDB-GENE-041121-17	-	XP_024250284.1	Chinook salmon
MamA	ZDB-GENE-040426-866	ubiquitin-protein ligase	XP_024250483.1	Chinook salmon
MamH	ZDB-GENE-030131-2165	DNA-binding transcription factor	XP_024251066.1	Chinook salmon
MamE	ZDB-GENE-031006-7	-	XP_024251777.1	Chinook salmon
Mad29	ZDB-GENE-040625-120	transporter	XP_024252163.1	Chinook salmon
MamK	ZDB-GENE-040426-2894	actin and actin related protein	XP_024252418.1	Chinook salmon
MamA	ZDB-GENE-050417-350	microtubule binding motor protein	XP_024252526.1	Chinook salmon

uMGH Category	ZFIN gene code	PANTHER protein class	Accession	Fish
MamA	ZDB-GENE-040704-72	-	XP_024252588.1	Chinook salmon
MamK	ZDB-GENE-040426-2695	actin and actin related protein	XP_024252934.1	Chinook salmon
MamB	ZDB-GENE-040718-6	-	XP_024253167.1	Chinook salmon
MamK	ZDB-GENE-031001-11	-	XP_024253253.1	Chinook salmon
MamE	ZDB-GENE-060531-24	-	XP_024253264.1	Chinook salmon
MamA	ZDB-GENE-110203-5	-	XP_024253278.1	Chinook salmon
Mad17	ZDB-GENE-030131-8767	-	XP_024253384.1	Chinook salmon
MamA	ZDB-GENE-040426-866	ubiquitin-protein ligase	XP_024253574.1	Chinook salmon
MamA	ZDB-GENE-041121-17	-	XP_024253745.1	Chinook salmon
Mad9	ZDB-GENE-040426-2153	oxidoreductase	XP_024254386.1	Chinook salmon
Mad9	ZDB-GENE-040426-1995	-	XP_024254617.1	Chinook salmon
MamA	ZDB-GENE-120727-22	protein modifying enzyme	XP_024255636.1	Chinook salmon
MamA	ZDB-GENE-000831-4	-	XP_024255720.1	Chinook salmon
MamA	ZDB-GENE-141219-15	-	XP_024255819.1	Chinook salmon
Mad25	ZDB-GENE-030115-3	non-receptor serine/threonine protein kinase	XP_024255941.1	Chinook salmon
MamA	ZDB-GENE-040426-1599	microtubule binding motor protein	XP_024256032.1	Chinook salmon
MamA	ZDB-GENE-090429-2	-	XP_024256802.1	Chinook salmon
MamA	ZDB-GENE-110203-5	-	XP_024256927.1	Chinook salmon
MamE	ZDB-GENE-060531-24	-	XP_024256947.1	Chinook salmon
MamK	ZDB-GENE-031001-11	-	XP_024256954.1	Chinook salmon
MamA	ZDB-GENE-120727-22	protein modifying enzyme	XP_024257062.1	Chinook salmon
MamA	ZDB-GENE-070209-29	protein-binding activity modulator	XP_024257091.1	Chinook salmon
MamA	ZDB-GENE-040426-2483	chaperone	XP_024257292.1	Chinook salmon
MamA	ZDB-GENE-000831-4	-	XP_024257505.1	Chinook salmon
MamA	ZDB-GENE-041010-223	-	XP_024257612.1	Chinook salmon
MamE	ZDB-GENE-130530-682	-	XP_024257776.1	Chinook salmon
MamA	ZDB-GENE-070209-29	protein-binding activity modulator	XP_024257788.1	Chinook salmon

uMGH Category	ZFIN gene code	PANTHER protein class	Accession	Fish
Man6	ZDB-GENE-060503-341	histone modifying enzyme	XP_024257807.1	Chinook salmon
MamE	ZDB-GENE-050302-100	-	XP_024257967.1	Chinook salmon
MamA	ZDB-GENE-040426-1849	chaperone	XP_024258320.1	Chinook salmon
MamE	ZDB-GENE-030131-3200	-	XP_024258450.1	Chinook salmon
MamE	ZDB-GENE-030131-3200	-	XP_024258640.1	Chinook salmon
MamA	ZDB-GENE-051113-132	ubiquitin-protein ligase	XP_024258785.1	Chinook salmon
MamA	ZDB-GENE-030131-2527	-	XP_024258863.1	Chinook salmon
MamK	ZDB-GENE-070424-10	actin and actin related protein	XP_024259230.1	Chinook salmon
MamE	ZDB-GENE-070925-1	tight junction	XP_024259264.1	Chinook salmon
MamK	ZDB-GENE-081105-139	transmembrane signal receptor	XP_024259359.1	Chinook salmon
MamK	ZDB-GENE-081105-139	transmembrane signal receptor	XP_024259365.1	Chinook salmon
MamK	ZDB-GENE-081105-147	transmembrane signal receptor	XP_024259367.1	Chinook salmon
MamK	ZDB-GENE-040520-4	actin and actin related protein	XP_024259889.1	Chinook salmon
MamE	ZDB-GENE-031001-2	tight junction	XP_024259973.1	Chinook salmon
MamA	ZDB-GENE-130530-956	-	XP_024260166.1	Chinook salmon
MamA	ZDB-GENE-090312-172	chaperone	XP_024260758.1	Chinook salmon
MamA	ZDB-GENE-030131-9443	-	XP_024260963.1	Chinook salmon
Mad9	ZDB-GENE-040426-1995	-	XP_024261034.1	Chinook salmon
Mad17	ZDB-GENE-030131-5063	G-protein	XP_024261105.1	Chinook salmon
Mad25	ZDB-GENE-121214-181	membrane traffic protein	XP_024262540.1	Chinook salmon
MamE	ZDB-GENE-030616-129	-	XP_024262865.1	Chinook salmon
MamE	ZDB-GENE-030616-129	-	XP_024262951.1	Chinook salmon
MamN/MamE	ZDB-GENE-091113-42	-	XP_024263273.1	Chinook salmon
MamE	ZDB-GENE-040426-1529	scaffold/adaptor protein	XP_024263285.1	Chinook salmon
MamA	ZDB-GENE-101021-4	ubiquitin-protein ligase	XP_024263382.1	Chinook salmon
MamA	ZDB-GENE-081104-93	scaffold/adaptor protein	XP_024264166.1	Chinook salmon
MamA	ZDB-GENE-030131-574	-	XP_024264486.1	Chinook salmon

uMGH Category	ZFIN gene code	PANTHER protein class	Accession	Fish
MamE	ZDB-GENE-090313-354	-	XP_024264568.1	Chinook salmon
Mad17	ZDB-GENE-041114-12	RNA methyltransferase	XP_024264670.1	Chinook salmon
MamA	ZDB-GENE-030131-8173	primary active transporter	XP_024264851.1	Chinook salmon
MamK	ZDB-GENE-000329-3	actin and actin related protein	XP_024264891.1	Chinook salmon
MamA	ZDB-GENE-051120-15	-	XP_024265145.1	Chinook salmon
MamE	ZDB-GENE-031222-1	-	XP_024265159.1	Chinook salmon
MamB	ZDB-GENE-041014-347	transporter	XP_024266292.1	Chinook salmon
Mad29	ZDB-GENE-040625-120	transporter	XP_024266660.1	Chinook salmon
MamA	ZDB-GENE-030131-2963	ubiquitin-protein ligase	XP_024266832.1	Chinook salmon
Mad29	ZDB-GENE-040625-120	transporter	XP_024267302.1	Chinook salmon
Mad17	ZDB-GENE-040426-2317	-	XP_024267358.1	Chinook salmon
MamB	ZDB-GENE-030131-5650	-	XP_024267897.1	Chinook salmon
MamA	ZDB-GENE-010319-27	-	XP_024268035.1	Chinook salmon
MamN/MamE	ZDB-GENE-080430-1	-	XP_024268223.1	Chinook salmon
MamB	ZDB-GENE-131127-443	G-protein coupled receptor	XP_024268465.1	Chinook salmon
Mad29	ZDB-GENE-040718-140	-	XP_024268606.1	Chinook salmon
MamA	ZDB-GENE-070105-3	-	XP_024268633.1	Chinook salmon
MamA	ZDB-GENE-030131-514	chaperone	XP_024268889.1	Chinook salmon
MamA	ZDB-GENE-050417-158	-	XP_024268915.1	Chinook salmon
MamA	ZDB-GENE-050208-116	chaperone	XP_024269300.1	Chinook salmon
MamK	ZDB-GENE-040520-4	actin and actin related protein	XP_024269370.1	Chinook salmon
MamK	ZDB-GENE-040520-4	actin and actin related protein	XP_024269756.1	Chinook salmon
MamB	ZDB-GENE-041014-347	transporter	XP_024270396.1	Chinook salmon
MamA	ZDB-GENE-041010-223	-	XP_024270611.1	Chinook salmon
MamE	ZDB-GENE-050302-100	-	XP_024271107.1	Chinook salmon
MamA	ZDB-GENE-040426-1599	microtubule binding motor protein	XP_024271153.1	Chinook salmon
Mad25	ZDB-GENE-030115-3	non-receptor serine/threonine protein kinase	XP_024271173.1	Chinook salmon

uMGH Category	ZFIN gene code	PANTHER protein class	Accession	Fish
MamK	ZDB-GENE-040426-1221	-	XP_024271380.1	Chinook salmon
MamA	ZDB-GENE-110125-2	-	XP_024271599.1	Chinook salmon
MamE	ZDB-GENE-030131-8525	serine protease	XP_024271690.1	Chinook salmon
MamA	ZDB-GENE-040625-34	chaperone	XP_024272178.1	Chinook salmon
MamA	ZDB-GENE-030131-8173	primary active transporter	XP_024272393.1	Chinook salmon
MamB	ZDB-GENE-040426-1408	transporter	XP_024272617.1	Chinook salmon
MamA	ZDB-GENE-081104-113	ubiquitin-protein ligase	XP_024273077.1	Chinook salmon
MamK	ZDB-GENE-040426-2695	actin and actin related protein	XP_024273249.1	Chinook salmon
MamA	ZDB-GENE-020919-3	-	XP_024273502.1	Chinook salmon
MamN/MamE	ZDB-GENE-131120-192	serine protease	XP_024273688.1	Chinook salmon
MamN/MamE	ZDB-GENE-131120-192	serine protease	XP_024273792.1	Chinook salmon
MamE	ZDB-GENE-060526-377	cytoskeletal protein	XP_024273949.1	Chinook salmon
MamE	ZDB-GENE-030131-9439	-	XP_024274502.1	Chinook salmon
MamA	ZDB-GENE-030131-3782	general transcription factor	XP_024275133.1	Chinook salmon
MamB	ZDB-GENE-040426-1840	-	XP_024275476.1	Chinook salmon
MamA	ZDB-GENE-040426-694	RNA splicing factor	XP_024275566.1	Chinook salmon
MamK	ZDB-GENE-040724-72	actin and actin related protein	XP_024275613.1	Chinook salmon
Man6	ZDB-GENE-040724-213	extracellular matrix protein	XP_024275664.1	Chinook salmon
MamA	ZDB-GENE-080722-25	-	XP_024275837.1	Chinook salmon
MamA	ZDB-GENE-041010-223	-	XP_024276298.1	Chinook salmon
MamE	ZDB-GENE-030616-129	-	XP_024276779.1	Chinook salmon
MamA	ZDB-GENE-030131-8811	-	XP_024276844.1	Chinook salmon
Mad25	ZDB-GENE-060503-274	membrane traffic protein	XP_024276987.1	Chinook salmon
MamE	ZDB-GENE-030828-10	tight junction	XP_024277063.1	Chinook salmon
Man6	ZDB-GENE-060503-341	histone modifying enzyme	XP_024277322.1	Chinook salmon
MamA	ZDB-GENE-040426-1849	chaperone	XP_024277334.1	Chinook salmon
MamE	ZDB-GENE-030131-9439	-	XP_024277654.1	Chinook salmon

uMGH Category	ZFIN gene code	PANTHER protein class	Accession	Fish
Man6	ZDB-GENE-021226-1	extracellular matrix protein	XP_024277800.1	Chinook salmon
MamA	ZDB-GENE-070705-298	membrane trafficking regulatory protein	XP_024278281.1	Chinook salmon
MamA	ZDB-GENE-041014-158	-	XP_024278416.1	Chinook salmon
MamB	ZDB-GENE-041014-347	transporter	XP_024278456.1	Chinook salmon
MamA	ZDB-GENE-070705-298	membrane trafficking regulatory protein	XP_024278617.1	Chinook salmon
MamA	ZDB-GENE-030131-8811	-	XP_024278812.1	Chinook salmon
Mad25	ZDB-GENE-060503-274	membrane traffic protein	XP_024278987.1	Chinook salmon
MamE	ZDB-GENE-040426-2830	-	XP_024279006.1	Chinook salmon
MamA	ZDB-GENE-050208-116	chaperone	XP_024279394.1	Chinook salmon
MamA	ZDB-GENE-030131-514	chaperone	XP_024279462.1	Chinook salmon
MamA	ZDB-GENE-050417-158	-	XP_024279571.1	Chinook salmon
MamA	ZDB-GENE-041210-299	protein modifying enzyme	XP_024279651.1	Chinook salmon
Mad25	ZDB-GENE-131121-533	-	XP_024279682.1	Chinook salmon
Mad25/Man6	ZDB-GENE-021226-2	extracellular matrix protein	XP_024279810.1	Chinook salmon
Man6	ZDB-GENE-021226-1	extracellular matrix protein	XP_024279811.1	Chinook salmon
MamK	ZDB-GENE-041111-251	GTPase-activating protein	XP_024279840.1	Chinook salmon
MamK	ZDB-GENE-040520-4	actin and actin related protein	XP_024279885.1	Chinook salmon
MamE	ZDB-GENE-090313-191	cytoskeletal protein	XP_024280049.1	Chinook salmon
MamA	ZDB-GENE-050417-350	microtubule binding motor protein	XP_024280052.1	Chinook salmon
MamA	ZDB-GENE-010319-27	-	XP_024280298.1	Chinook salmon
MamA	ZDB-GENE-030102-1	-	XP_024280520.1	Chinook salmon
MamE	ZDB-GENE-040704-64	serine protease	XP_024280749.1	Chinook salmon
MamE	ZDB-GENE-030131-8525	serine protease	XP_024280923.1	Chinook salmon
MamE	ZDB-GENE-030131-3149	scaffold/adaptor protein	XP_024280942.1	Chinook salmon
MamA	ZDB-GENE-051120-15	-	XP_024280982.1	Chinook salmon
MamK	ZDB-GENE-040426-2894	actin and actin related protein	XP_024280998.1	Chinook salmon

uMGH Category	ZFIN gene code	PANTHER protein class	Accession	Fish
MamA	ZDB-GENE-081105-56	chromatin/chromatin-binding, or -regulatory protein	XP_024281352.1	Chinook salmon
Mad17	ZDB-GENE-030131-5063	G-protein	XP_024281439.1	Chinook salmon
MamB	ZDB-GENE-091204-410	-	XP_024281956.1	Chinook salmon
MamA	ZDB-GENE-050417-350	microtubule binding motor protein	XP_024282077.1	Chinook salmon
MamA	ZDB-GENE-030131-2081	-	XP_024282175.1	Chinook salmon
MamE	ZDB-GENE-080717-1	C2H2 zinc finger transcription factor	XP_024282193.1	Chinook salmon
Mad25	ZDB-GENE-040516-8	protein-binding activity modulator	XP_024282840.1	Chinook salmon
MamE	ZDB-GENE-040704-64	serine protease	XP_024283543.1	Chinook salmon
MamA	ZDB-GENE-081105-56	chromatin/chromatin-binding, or -regulatory protein	XP_024283947.1	Chinook salmon
Man6	ZDB-GENE-070615-37	chromatin/chromatin-binding, or -regulatory protein	XP_024283987.1	Chinook salmon
MamA	ZDB-GENE-000831-4	-	XP_024284062.1	Chinook salmon
MamA	ZDB-GENE-000831-4	-	XP_024284077.1	Chinook salmon
MamK	ZDB-GENE-000329-3	actin and actin related protein	XP_024284211.1	Chinook salmon
MamB	ZDB-GENE-040426-1838	transporter	XP_024284293.1	Chinook salmon
Mad17	ZDB-GENE-130530-693	protein modifying enzyme	XP_024284401.1	Chinook salmon
Man6	ZDB-GENE-061103-409	-	XP_024284625.1	Chinook salmon
MamA	ZDB-GENE-051127-17	-	XP_024284918.1	Chinook salmon
MamE	ZDB-GENE-030131-3149	scaffold/adaptor protein	XP_024284986.1	Chinook salmon
Mad9	ZDB-GENE-050522-131	oxidoreductase	XP_024285127.1	Chinook salmon
MamA	ZDB-GENE-040625-34	chaperone	XP_024285285.1	Chinook salmon
MamE	ZDB-GENE-030131-6959	guanyl-nucleotide exchange factor	XP_024285287.1	Chinook salmon
MamA	ZDB-GENE-051120-15	-	XP_024285623.1	Chinook salmon
MamA	ZDB-GENE-070112-1282	serine protease	XP_024285644.1	Chinook salmon
MamK	ZDB-GENE-040520-4	actin and actin related protein	XP_024285818.1	Chinook salmon
MamA	ZDB-GENE-070105-3	-	XP_024286076.1	Chinook salmon
MamE	ZDB-GENE-030616-572	scaffold/adaptor protein	XP_024286214.1	Chinook salmon

uMGH Category	ZFIN gene code	PANTHER protein class	Accession	Fish
MamH	ZDB-GENE-050417-386	secondary carrier transporter	XP_024286242.1	Chinook salmon
MamB	ZDB-GENE-041014-347	transporter	XP_024286295.1	Chinook salmon
MamE	ZDB-GENE-031006-7	-	XP_024286992.1	Chinook salmon
MamA	ZDB-GENE-090313-3	ubiquitin-protein ligase	XP_024287685.1	Chinook salmon
MamA	ZDB-GENE-050302-4	chaperone	XP_024287707.1	Chinook salmon
MamK	ZDB-GENE-990415-92	-	XP_024287800.1	Chinook salmon
MamK	ZDB-GENE-990415-92	-	XP_024287807.1	Chinook salmon
MamE	ZDB-GENE-030131-6972	guanyl-nucleotide exchange factor	XP_024287983.1	Chinook salmon
MamK	ZDB-GENE-990415-92	-	XP_024288068.1	Chinook salmon
MamK	ZDB-GENE-990415-92	-	XP_024288075.1	Chinook salmon
MamE	ZDB-GENE-030131-6972	guanyl-nucleotide exchange factor	XP_024288248.1	Chinook salmon
MamA	ZDB-GENE-041114-80	-	XP_024288382.1	Chinook salmon
MamA	ZDB-GENE-990706-2	ubiquitin-protein ligase	XP_024288674.1	Chinook salmon
MamK	ZDB-GENE-110713-1	-	XP_024288929.1	Chinook salmon
MamA	ZDB-GENE-040426-2483	chaperone	XP_024288930.1	Chinook salmon
MamA	ZDB-GENE-030131-5395	-	XP_024289178.1	Chinook salmon
MamB	ZDB-GENE-040724-254	transporter	XP_024289677.1	Chinook salmon
MamE	ZDB-GENE-121019-1	-	XP_024289695.1	Chinook salmon
MamA	ZDB-GENE-080208-7	nucleic acid metabolism protein	XP_024289723.1	Chinook salmon
MamE	ZDB-GENE-130530-682	-	XP_024289739.1	Chinook salmon
Man6	ZDB-GENE-030131-9823	extracellular matrix protein	XP_024290010.1	Chinook salmon
MamE	ZDB-GENE-040801-245	serine protease	XP_024290137.1	Chinook salmon
MamK	ZDB-GENE-040520-4	actin and actin related protein	XP_024290181.1	Chinook salmon
MamK	ZDB-GENE-040520-4	actin and actin related protein	XP_024290182.1	Chinook salmon
MamK	ZDB-GENE-041121-7	actin and actin related protein	XP_024290301.1	Chinook salmon
MamA	ZDB-GENE-000831-4	-	XP_024290415.1	Chinook salmon
MamA	ZDB-GENE-070705-298	membrane trafficking regulatory protein	XP_024290798.1	Chinook salmon

uMGH Category	ZFIN gene code	PANTHER protein class	Accession	Fish
MamA	ZDB-GENE-070912-490	-	XP_024290803.1	Chinook salmon
MamE	ZDB-GENE-130530-876	scaffold/adaptor protein	XP_024291018.1	Chinook salmon
Man6	ZDB-GENE-021226-3	extracellular matrix protein	XP_024291330.1	Chinook salmon
Mad25	ZDB-GENE-100712-2	non-receptor serine/threonine protein kinase	XP_024291697.1	Chinook salmon
MamA	ZDB-GENE-040426-1169	-	XP_024291998.1	Chinook salmon
MamA	ZDB-GENE-050417-350	microtubule binding motor protein	XP_024292119.1	Chinook salmon
MamA	ZDB-GENE-030131-4744	chaperone	XP_024292265.1	Chinook salmon
MamA	ZDB-GENE-080728-4	protein modifying enzyme	XP_024292308.1	Chinook salmon
MamA	ZDB-GENE-080728-4	protein modifying enzyme	XP_024292320.1	Chinook salmon
MamA	ZDB-GENE-090429-2	-	XP_024292816.1	Chinook salmon
MamK	ZDB-GENE-040625-35	actin and actin related protein	XP_024292865.1	Chinook salmon
Mad25	ZDB-GENE-030115-3	non-receptor serine/threonine protein kinase	XP_024292898.1	Chinook salmon
MamA	ZDB-GENE-040426-1599	microtubule binding motor protein	XP_024292999.1	Chinook salmon
MamK	ZDB-GENE-040520-4	actin and actin related protein	XP_024293216.1	Chinook salmon
MamB	ZDB-GENE-041014-347	transporter	XP_024293297.1	Chinook salmon
MamH	ZDB-GENE-050417-386	secondary carrier transporter	XP_024293739.1	Chinook salmon
MamA	ZDB-GENE-020919-3	-	XP_024294314.1	Chinook salmon
MamE	ZDB-GENE-070308-4	actin or actin-binding cytoskeletal protein	XP_024294537.1	Chinook salmon
MamK	ZDB-GENE-040426-2695	actin and actin related protein	XP_024294607.1	Chinook salmon
Mad29	ZDB-GENE-040625-120	transporter	XP_024295385.1	Chinook salmon
MamE	ZDB-GENE-031001-2	tight junction	XP_024295795.1	Chinook salmon
MamA	ZDB-GENE-030131-3782	general transcription factor	XP_024295928.1	Chinook salmon
MamA	ZDB-GENE-060126-2	-	XP_024296284.1	Chinook salmon
MamE	ZDB-GENE-060526-377	cytoskeletal protein	XP_024296314.1	Chinook salmon
MamA	ZDB-GENE-081104-113	ubiquitin-protein ligase	XP_024296461.1	Chinook salmon
MamA	ZDB-GENE-100422-15	-	XP_024296581.1	Chinook salmon
MamH	ZDB-GENE-030131-2165	DNA-binding transcription factor	XP_024296711.1	Chinook salmon

uMGH Category	ZFIN gene code	PANTHER protein class	Accession	Fish
MamA	ZDB-GENE-071016-4	-	XP_024297837.1	Chinook salmon
Mad25	ZDB-GENE-141215-28	membrane traffic protein	XP_024298089.1	Chinook salmon
MamK	ZDB-GENE-040520-4	actin and actin related protein	XP_024298139.1	Chinook salmon
MamA	ZDB-GENE-060929-772	microtubule binding motor protein	XP_024298228.1	Chinook salmon
MamK	ZDB-GENE-040426-2894	actin and actin related protein	XP_024298298.1	Chinook salmon
Man6	ZDB-GENE-121114-8	-	XP_024298417.1	Chinook salmon
MamN	ZDB-GENE-030131-4628	secondary carrier transporter	XP_024298672.1	Chinook salmon
MamK	ZDB-GENE-990415-92	-	XP_024298732.1	Chinook salmon
MamA	ZDB-GENE-050417-350	microtubule binding motor protein	XP_024299050.1	Chinook salmon
MamA	ZDB-GENE-050327-75	protein phosphatase	XP_024299096.1	Chinook salmon
Mad17	ZDB-GENE-030131-5063	G-protein	XP_024299617.1	Chinook salmon
MamE	ZDB-GENE-060503-371	scaffold/adaptor protein	XP_024300068.1	Chinook salmon
MamA	ZDB-GENE-030131-2081	-	XP_024300141.1	Chinook salmon
MamA	ZDB-GENE-050419-67	chaperone	XP_024300276.1	Chinook salmon
MamA	ZDB-GENE-060929-772	microtubule binding motor protein	XP_024300555.1	Chinook salmon
MamK	ZDB-GENE-070410-58	-	XP_024301220.1	Chinook salmon
MamE	ZDB-GENE-031006-7	-	ENSDARG00000000068.9	Zebrafish
MamE	ZDB-GENE-030616-572	scaffold/adaptor protein	ENSDARG00000000861.13	Zebrafish
MamA	ZDB-GENE-030131-2081	-	ENSDARG00000001557.9	Zebrafish
MamA	ZDB-GENE-040426-1849	chaperone	ENSDARG00000001734.10	Zebrafish
MamA	ZDB-GENE-050208-116	chaperone	ENSDARG00000001976.9	Zebrafish
Mad25/Man6	ZDB-GENE-081030-4	extracellular matrix protein	ENSDARG00000002084.11	Zebrafish
MamE	ZDB-GENE-030828-10	tight junction	ENSDARG00000002909.11	Zebrafish
MamK	ZDB-GENE-030828-12	-	ENSDARG00000003035.10	Zebrafish
MamA	ZDB-GENE-030131-9443	-	ENSDARG00000004017.10	Zebrafish
MamK	ZDB-GENE-041121-7	actin and actin related protein	ENSDARG00000004658.9	Zebrafish
Mad25	ZDB-GENE-060125-2	non-receptor serine/threonine protein kinase	ENSDARG00000004877.14	Zebrafish

uMGH Category	ZFIN gene code	PANTHER protein class	Accession	Fish
MamA	ZDB-GENE-041121-17	-	ENSDARG00000004906.9	Zebrafish
MamE	ZDB-GENE-030219-74	-	ENSDARG00000004930.11	Zebrafish
MamB	ZDB-GENE-040426-1840	-	ENSDARG00000005463.6	Zebrafish
MamE	ZDB-GENE-030131-6959	guanyl-nucleotide exchange factor	ENSDARG00000005482.9	Zebrafish
MamA	ZDB-GENE-070209-29	protein-binding activity modulator	ENSDARG00000005821.11	Zebrafish
MamN/MamE	ZDB-GENE-131120-192	serine protease	ENSDARG00000005943.10	Zebrafish
MamE	ZDB-GENE-030131-431	protease	ENSDARG00000006300.9	Zebrafish
MamB	ZDB-GENE-040426-1408	transporter	ENSDARG00000007180.9	Zebrafish
MamA	ZDB-GENE-121214-248	-	ENSDARG00000007467.10	Zebrafish
MamA	ZDB-GENE-040426-694	RNA splicing factor	ENSDARG00000007901.9	Zebrafish
MamA	ZDB-GENE-030102-1	-	ENSDARG00000007918.12	Zebrafish
MamA	ZDB-GENE-121214-233	-	ENSDARG00000008098.9	Zebrafish
MamA	ZDB-GENE-030131-514	chaperone	ENSDARG00000008447.11	Zebrafish
MamA	ZDB-GENE-030131-3782	general transcription factor	ENSDARG00000009170.10	Zebrafish
MamA	ZDB-GENE-040426-1599	microtubule binding motor protein	ENSDARG00000009796.11	Zebrafish
MamE	ZDB-GENE-030131-6972	guanyl-nucleotide exchange factor	ENSDARG00000010945.9	Zebrafish
MamK	ZDB-GENE-040426-2695	actin and actin related protein	ENSDARG00000011611.10	Zebrafish
MamH	ZDB-GENE-020228-1	transporter	ENSDARG00000011925.9	Zebrafish
MamA	ZDB-GENE-031010-34	-	ENSDARG00000012368.13	Zebrafish
MamK	ZDB-GENE-040426-1221	-	ENSDARG00000012381.9	Zebrafish
MamE	ZDB-GENE-030131-3727	membrane trafficking regulatory protein	ENSDARG00000012513.7	Zebrafish
MamE	ZDB-GENE-080219-7	serine protease	ENSDARG00000014907.8	Zebrafish
MamE	ZDB-GENE-041210-125	-	ENSDARG00000015053.10	Zebrafish
MamA	ZDB-GENE-040704-72	-	ENSDARG00000016769.11	Zebrafish
MamA	ZDB-GENE-040426-1169	-	ENSDARG00000017311.11	Zebrafish
Mad25	ZDB-GENE-030115-3	non-receptor serine/threonine protein kinase	ENSDARG00000017500.12	Zebrafish
MamA	ZDB-GENE-000831-4	-	ENSDARG00000017874.11	Zebrafish

uMGH Category	ZFIN gene code	PANTHER protein class	Accession	Fish
Mad29	ZDB-GENE-040625-120	transporter	ENSDARG00000018190.10	Zebrafish
MamA	ZDB-GENE-030131-8811	-	ENSDARG00000019941.8	Zebrafish
MamB	ZDB-GENE-030131-5650	-	ENSDARG00000019998.9	Zebrafish
MamA	ZDB-GENE-070912-148	-	ENSDARG00000020447.11	Zebrafish
Man6	ZDB-GENE-040426-809	scaffold/adaptor protein	ENSDARG00000020764.9	Zebrafish
Man6	ZDB-GENE-040724-213	extracellular matrix protein	ENSDARG00000020785.10	Zebrafish
MamB	ZDB-GENE-041014-347	transporter	ENSDARG00000021305.12	Zebrafish
MamK	ZDB-GENE-110713-1	-	ENSDARG00000021924.9	Zebrafish
MamA	ZDB-GENE-030131-5395	-	ENSDARG00000021973.8	Zebrafish
MamE	ZDB-GENE-031222-1	-	ENSDARG00000022261.9	Zebrafish
MamA	ZDB-GENE-070705-298	membrane trafficking regulatory protein	ENSDARG00000022518.11	Zebrafish
MamE	ZDB-GENE-040718-58	tight junction	ENSDARG00000023443.10	Zebrafish
MamE	ZDB-GENE-030616-129	-	ENSDARG00000024964.9	Zebrafish
MamE	ZDB-GENE-081031-78	-	ENSDARG00000026840.11	Zebrafish
MamA	ZDB-GENE-030131-574	-	ENSDARG00000027234.11	Zebrafish
MamE	ZDB-GENE-070705-172	-	ENSDARG00000027618.12	Zebrafish
MamA	ZDB-GENE-030616-630	chaperone	ENSDARG00000028396.5	Zebrafish
MamA	ZDB-GENE-030131-8173	primary active transporter	ENSDARG00000029639.8	Zebrafish
MamE	ZDB-GENE-081104-57	membrane trafficking regulatory protein	ENSDARG00000030097.7	Zebrafish
MamA	ZDB-GENE-050302-4	chaperone	ENSDARG00000030133.7	Zebrafish
MamE	ZDB-GENE-040704-64	serine protease	ENSDARG00000032831.6	Zebrafish
Man6	ZDB-GENE-030131-4205	extracellular matrix protein	ENSDARG00000033950.9	Zebrafish
MamA	ZDB-GENE-050327-75	protein phosphatase	ENSDARG00000034313.9	Zebrafish
MamA	ZDB-GENE-081031-100	protein modifying enzyme	ENSDARG00000035273.8	Zebrafish
MamA	ZDB-GENE-040718-480	-	ENSDARG00000035406.5	Zebrafish
Man6	ZDB-GENE-021226-3	extracellular matrix protein	ENSDARG00000036279.6	Zebrafish
MamK	ZDB-GENE-050417-267	actin and actin related protein	ENSDARG00000036371.5	Zebrafish

uMGH Category	ZFIN gene code	PANTHER protein class	Accession	Fish
MamK	ZDB-GENE-121214-31	-	ENSDARG00000037403.6	Zebrafish
MamK	ZDB-GENE-000329-1	actin and actin related protein	ENSDARG00000037746.7	Zebrafish
MamK	ZDB-GENE-000329-3	actin and actin related protein	ENSDARG00000037870.6	Zebrafish
MamA	ZDB-GENE-040625-34	chaperone	ENSDARG00000038835.7	Zebrafish
MamA	ZDB-GENE-051120-15	-	ENSDARG00000039082.9	Zebrafish
Mad25/Man6	ZDB-GENE-021226-2	extracellular matrix protein	ENSDARG00000039133.8	Zebrafish
MamA	ZDB-GENE-030131-5511	chaperone	ENSDARG00000039208.9	Zebrafish
Mad17	ZDB-GENE-030131-8767	-	ENSDARG00000039345.6	Zebrafish
Mad17	ZDB-GENE-040718-153	-	ENSDARG00000040300.6	Zebrafish
MamN/MamE	ZDB-GENE-080430-1	-	ENSDARG00000040568.5	Zebrafish
MamK	ZDB-GENE-070410-58	-	ENSDARG00000040984.7	Zebrafish
MamA	ZDB-GENE-030131-1264	-	ENSDARG00000041110.4	Zebrafish
MamK	ZDB-GENE-040520-4	actin and actin related protein	ENSDARG00000042535.5	Zebrafish
Mad25	ZDB-GENE-060503-274	membrane traffic protein	ENSDARG00000042670.10	Zebrafish
MamE	ZDB-GENE-060421-4490	secondary carrier transporter	ENSDARG00000042737.7	Zebrafish
MamH	ZDB-GENE-081021-1	secondary carrier transporter	ENSDARG00000042954.8	Zebrafish
MamA	ZDB-GENE-040914-80	general transcription factor	ENSDARG00000043247.6	Zebrafish
MamE	ZDB-GENE-030131-9439	-	ENSDARG00000043323.9	Zebrafish
MamK	ZDB-GENE-040724-72	actin and actin related protein	ENSDARG00000043963.8	Zebrafish
MamH	ZDB-GENE-050417-386	secondary carrier transporter	ENSDARG00000044047.7	Zebrafish
MamA	ZDB-GENE-040426-866	ubiquitin-protein ligase	ENSDARG00000044484.5	Zebrafish
Mad17	ZDB-GENE-030131-5063	G-protein	ENSDARG00000044565.8	Zebrafish
MamA	ZDB-GENE-100422-15	-	ENSDARG00000044812.8	Zebrafish
MamK	ZDB-GENE-030131-1229	actin and actin related protein	ENSDARG00000045180.9	Zebrafish
MamA	ZDB-GENE-030131-2963	ubiquitin-protein ligase	ENSDARG00000045228.8	Zebrafish
Mad25/Man6	ZDB-GENE-041210-197	extracellular matrix protein	ENSDARG00000045524.7	Zebrafish
MamE	ZDB-GENE-041014-2	serine protease	ENSDARG00000045544.7	Zebrafish

uMGH Category	ZFIN gene code	PANTHER protein class	Accession	Fish
MamE	ZDB-GENE-041210-237	-	ENSDARG00000045687.7	Zebrafish
MamA	ZDB-GENE-041210-299	protein modifying enzyme	ENSDARG00000045753.6	Zebrafish
MamB	ZDB-GENE-040718-6	-	ENSDARG00000051921.4	Zebrafish
Mad9	ZDB-GENE-040426-2153	oxidoreductase	ENSDARG00000051986.4	Zebrafish
MamE	ZDB-GENE-000329-5	oxidase	ENSDARG00000052139.8	Zebrafish
MamH	ZDB-GENE-051120-165	secondary carrier transporter	ENSDARG00000052271.8	Zebrafish
MamK	ZDB-GENE-040426-2894	actin and actin related protein	ENSDARG00000052438.6	Zebrafish
MamE	ZDB-GENE-040801-245	serine protease	ENSDARG00000052895.9	Zebrafish
MamE	ZDB-GENE-040426-2544	cell junction protein	ENSDARG00000053194.7	Zebrafish
Mad25	ZDB-GENE-080204-113	-	ENSDARG00000053201.7	Zebrafish
MamE	ZDB-GENE-060825-242	-	ENSDARG00000053535.8	Zebrafish
MamB	ZDB-GENE-070424-43	-	ENSDARG00000053896.6	Zebrafish
Mad29	ZDB-GENE-040718-140	-	ENSDARG00000054821.6	Zebrafish
MamH	ZDB-GENE-060929-1158	secondary carrier transporter	ENSDARG00000055190.6	Zebrafish
MamA	ZDB-GENE-051113-132	ubiquitin-protein ligase	ENSDARG00000055470.6	Zebrafish
Mad29	ZDB-GENE-050417-471	-	ENSDARG00000055472.7	Zebrafish
MamK	ZDB-GENE-030131-55	actin and actin related protein	ENSDARG00000055618.5	Zebrafish
Mad29	ZDB-GENE-051023-10	-	ENSDARG00000055653.7	Zebrafish
MamE	ZDB-GENE-081022-151	-	ENSDARG00000055656.4	Zebrafish
MamK	ZDB-GENE-050321-1	-	ENSDARG00000055723.6	Zebrafish
MamA	ZDB-GENE-060929-772	microtubule binding motor protein	ENSDARG00000055965.6	Zebrafish
MamK	ZDB-GENE-060503-867	-	ENSDARG00000056210.7	Zebrafish
MamA	ZDB-GENE-990706-2	ubiquitin-protein ligase	ENSDARG00000056258.8	Zebrafish
MamA	ZDB-GENE-071016-4	-	ENSDARG00000056896.6	Zebrafish
Mad9	ZDB-GENE-050522-131	oxidoreductase	ENSDARG00000057055.9	Zebrafish
MamA	ZDB-GENE-051127-17	-	ENSDARG00000057173.4	Zebrafish
MamB	ZDB-GENE-040724-254	transporter	ENSDARG00000057272.8	Zebrafish

uMGH Category	ZFIN gene code	PANTHER protein class	Accession	Fish
MamA	ZDB-GENE-030131-2527	-	ENSDARG00000057890.7	Zebrafish
MamK	ZDB-GENE-040625-35	actin and actin related protein	ENSDARG00000057911.7	Zebrafish
Man6	ZDB-GENE-030131-3404	-	ENSDARG00000057918.7	Zebrafish
MamA	ZDB-GENE-050522-556	-	ENSDARG00000057986.8	Zebrafish
Mad25	ZDB-GENE-040801-25	-	ENSDARG00000057997.6	Zebrafish
MamA	ZDB-GENE-040426-995	-	ENSDARG00000058140.5	Zebrafish
MamA	ZDB-GENE-040426-2483	chaperone	ENSDARG00000058148.8	Zebrafish
MamA	ZDB-GENE-091204-50	structural protein	ENSDARG00000058508.8	Zebrafish
Man6	ZDB-GENE-030131-9823	extracellular matrix protein	ENSDARG00000058543.6	Zebrafish
Man6	ZDB-GENE-070705-287	DNA metabolism protein	ENSDARG00000058719.9	Zebrafish
MamH	ZDB-GENE-080204-92	secondary carrier transporter	ENSDARG00000058775.6	Zebrafish
MamA	ZDB-GENE-100922-181	-	ENSDARG00000058885.7	Zebrafish
MamN	ZDB-GENE-070424-71	secondary carrier transporter	ENSDARG00000059053.8	Zebrafish
MamE	ZDB-GENE-040426-2830	-	ENSDARG00000059177.5	Zebrafish
MamE	ZDB-GENE-130530-682	-	ENSDARG00000059567.7	Zebrafish
MamA	ZDB-GENE-070112-2002	chromatin/chromatin-binding, or - regulatory protein	ENSDARG00000059794.7	Zebrafish
Mad17	ZDB-GENE-060526-84	RNA metabolism protein	ENSDARG00000059887.6	Zebrafish
MamA	ZDB-GENE-070112-1282	serine protease	ENSDARG00000060101.8	Zebrafish
MamA	ZDB-GENE-070105-3	-	ENSDARG00000061207.8	Zebrafish
MamB	ZDB-GENE-060929-448	-	ENSDARG00000061216.6	Zebrafish
MamN	ZDB-GENE-070718-4	primary active transporter	ENSDARG00000061303.8	Zebrafish
MamE	ZDB-GENE-090313-280	guanyl-nucleotide exchange factor	ENSDARG00000061459.7	Zebrafish
MamE	ZDB-GENE-140106-76	-	ENSDARG00000061717.6	Zebrafish
MamA	ZDB-GENE-081105-56	chromatin/chromatin-binding, or regulatory protein	ENSDARG00000061759.9	Zebrafish
MamH	ZDB-GENE-131120-103	secondary carrier transporter	ENSDARG00000062182.6	Zebrafish
MamA	ZDB-GENE-061221-2	-	ENSDARG00000062846.7	Zebrafish
MamA	ZDB-GENE-081104-113	ubiquitin-protein ligase	ENSDARG00000063005.7	Zebrafish

uMGH Category	ZFIN gene code	PANTHER protein class	Accession	Fish
MamA	ZDB-GENE-070705-219	-	ENSDARG00000063149.9	Zebrafish
MamA	ZDB-GENE-080722-25	-	ENSDARG00000063242.6	Zebrafish
MamA	ZDB-GENE-060126-2	-	ENSDARG00000063522.7	Zebrafish
MamE	ZDB-GENE-060526-377	cytoskeletal protein	ENSDARG00000068166.6	Zebrafish
MamA	ZDB-GENE-050419-67	chaperone	ENSDARG00000068421.4	Zebrafish
MamK	ZDB-GENE-990415-92	-	ENSDARG00000068992.5	Zebrafish
MamE	ZDB-GENE-081028-30	serine protease	ENSDARG00000069909.7	Zebrafish
MamE	ZDB-GENE-070308-4	actin or actin-binding cytoskeletal protein	ENSDARG00000069956.7	Zebrafish
MamK	ZDB-GENE-050410-4	actin and actin related protein	ENSDARG00000070076.6	Zebrafish
MamE	ZDB-GENE-091113-21	serine protease	ENSDARG00000070138.6	Zebrafish
MamH	ZDB-GENE-030131-2165	DNA-binding transcription factor	ENSDARG00000070151.8	Zebrafish
MamA	ZDB-GENE-040426-981	membrane trafficking regulatory protein	ENSDARG00000070654.6	Zebrafish
MamA	ZDB-GENE-041111-230	-	ENSDARG00000071294.5	Zebrafish
MamE	ZDB-GENE-030131-3149	scaffold/adaptor protein	ENSDARG00000074059.6	Zebrafish
MamA	ZDB-GENE-110125-2	-	ENSDARG00000074314.5	Zebrafish
MamA	ZDB-GENE-090312-172	chaperone	ENSDARG00000074363.5	Zebrafish
MamA	ZDB-GENE-111221-1	membrane trafficking regulatory protein	ENSDARG00000074403.5	Zebrafish
MamA	ZDB-GENE-010319-27	-	ENSDARG00000074760.5	Zebrafish
MamE	ZDB-GENE-030131-8525	serine protease	ENSDARG00000074895.5	Zebrafish
MamA	ZDB-GENE-101021-4	ubiquitin-protein ligase	ENSDARG00000075048.6	Zebrafish
MamE	ZDB-GENE-090313-90	protein phosphatase	ENSDARG00000075459.5	Zebrafish
MamA	ZDB-GENE-081107-12	ubiquitin-protein ligase	ENSDARG00000075687.5	Zebrafish
MamN/MamE	ZDB-GENE-091113-42	-	ENSDARG00000076103.6	Zebrafish
MamK	ZDB-GENE-040520-4	actin and actin related protein	ENSDARG00000076126.4	Zebrafish
MamE	ZDB-GENE-090313-191	cytoskeletal protein	ENSDARG00000076974.5	Zebrafish
MamB	ZDB-GENE-040426-1838	transporter	ENSDARG00000077368.5	Zebrafish

uMGH Category	ZFIN gene code	PANTHER protein class	Accession	Fish
MamE	ZDB-GENE-031001-2	tight junction	ENSDARG00000077506.8	Zebrafish
MamK	ZDB-GENE-070522-2	-	ENSDARG00000077866.5	Zebrafish
Mad25	ZDB-GENE-081104-264	membrane traffic protein	ENSDARG00000078216.5	Zebrafish
MamA	ZDB-GENE-091204-117	-	ENSDARG00000078261.7	Zebrafish
MamA	ZDB-GENE-120727-22	protein modifying enzyme	ENSDARG00000078703.4	Zebrafish
MamA	ZDB-GENE-060130-180	-	ENSDARG00000079097.6	Zebrafish
MamE	ZDB-GENE-070925-1	tight junction	ENSDARG00000079374.8	Zebrafish
MamE	ZDB-GENE-130530-876	scaffold/adaptor protein	ENSDARG00000079670.5	Zebrafish
MamE	ZDB-GENE-121214-224	-	ENSDARG00000079791.5	Zebrafish
MamA	ZDB-GENE-090313-3	ubiquitin-protein ligase	ENSDARG00000086075.4	Zebrafish
MamH	ZDB-GENE-030131-7188	secondary carrier transporter	ENSDARG00000086739.3	Zebrafish
MamE	ZDB-GENE-081104-132	-	ENSDARG00000087187.5	Zebrafish
MamA	ZDB-GENE-040426-928	-	ENSDARG00000087962.4	Zebrafish
MamB	ZDB-GENE-091204-410	-	ENSDARG00000088227.4	Zebrafish
MamE	ZDB-GENE-080215-8	serine protease	ENSDARG00000088743.3	Zebrafish
MamA	ZDB-GENE-130530-956	-	ENSDARG00000089190.3	Zebrafish
MamA	ZDB-GENE-121214-302	-	ENSDARG00000090537.3	Zebrafish
MamN/MamE	ZDB-GENE-090313-136	-	ENSDARG00000090564.4	Zebrafish
MamA	ZDB-GENE-120406-2	-	ENSDARG00000090977.4	Zebrafish
MamE	ZDB-GENE-081028-21	serine protease	ENSDARG00000092061.3	Zebrafish
MamE	ZDB-GENE-081028-22	serine protease	ENSDARG00000092094.4	Zebrafish
MamE	ZDB-GENE-091113-30	serine protease	ENSDARG00000092133.3	Zebrafish
MamE	ZDB-GENE-141219-11	serine protease	ENSDARG00000092202.3	Zebrafish
MamK	ZDB-GENE-110405-1	-	ENSDARG00000092362.4	Zebrafish
MamE	ZDB-GENE-081028-18	serine protease	ENSDARG00000092380.3	Zebrafish
MamE	ZDB-GENE-081028-29	serine protease	ENSDARG00000093005.3	Zebrafish
MamA	ZDB-GENE-081104-93	scaffold/adaptor protein	ENSDARG00000093125.5	Zebrafish

uMGH Category	ZFIN gene code	PANTHER protein class	Accession	Fish
MamN/MamE	ZDB-GENE-081028-43	serine protease	ENSDARG00000094236.3	Zebrafish
MamE	ZDB-GENE-081028-27	serine protease	ENSDARG00000094403.3	Zebrafish
MamE	ZDB-GENE-081104-136	-	ENSDARG00000094605.2	Zebrafish
MamE	ZDB-GENE-071004-51	serine protease	ENSDARG00000094741.3	Zebrafish
MamE	ZDB-GENE-091113-19	serine protease	ENSDARG00000094749.3	Zebrafish
MamE	ZDB-GENE-091112-23	serine protease	ENSDARG00000094878.3	Zebrafish
MamE	ZDB-GENE-091113-31	serine protease	ENSDARG00000094995.3	Zebrafish
MamE	ZDB-GENE-081028-23	serine protease	ENSDARG00000095314.3	Zebrafish
MamA	ZDB-GENE-030131-4744	chaperone	ENSDARG00000095322.4	Zebrafish
MamE	ZDB-GENE-081028-30	serine protease	ENSDARG00000095545.3	Zebrafish
MamN/MamE	ZDB-GENE-040724-161	-	ENSDARG00000095603.4	Zebrafish
MamE	ZDB-GENE-091113-30	serine protease	ENSDARG00000095687.3	Zebrafish
MamA	ZDB-GENE-120215-5	non-receptor serine/threonine protein kinase	ENSDARG00000096423.3	Zebrafish
MamK	ZDB-GENE-040426-2877	actin and actin related protein	ENSDARG00000098235.2	Zebrafish
MamA	ZDB-GENE-030131-4512	nucleic acid metabolism protein	ENSDARG00000098540.3	Zebrafish
MamE	ZDB-GENE-030131-1791	general transcription factor	ENSDARG00000098822.2	Zebrafish
MamK	ZDB-GENE-000322-1	actin and actin related protein	ENSDARG00000099197.3	Zebrafish
MamA	ZDB-GENE-030131-9631	protein modifying enzyme	ENSDARG00000099455.2	Zebrafish
MamB	ZDB-GENE-060315-10	transporter	ENSDARG00000100917.2	Zebrafish
Man6	ZDB-GENE-021226-1	extracellular matrix protein	ENSDARG00000101209.3	Zebrafish
MamA	ZDB-GENE-041010-223	-	ENSDARG00000102026.2	Zebrafish
Mad25/Man6	ZDB-GENE-060118-1	extracellular matrix protein	ENSDARG00000102277.2	Zebrafish
MamA	ZDB-GENE-050417-158	-	ENSDARG00000103643.3	Zebrafish
MamE	ZDB-GENE-070410-72	protein phosphatase	ENSDARG00000103699.3	Zebrafish
MamK	ZDB-GENE-031001-11	-	ENSDARG00000103846.3	Zebrafish
MamB	ZDB-GENE-060608-2	-	ENSDARG00000103983.2	Zebrafish

uMGH Category	ZFIN gene code	PANTHER protein class	Accession	Fish
MamA	ZDB-GENE-141219-15	-	ENSDARG00000104035.2	Zebrafish
MamA	ZDB-GENE-090429-2	-	ENSDARG00000104125.2	Zebrafish
MamA	ZDB-GENE-030131-9631	protein modifying enzyme	ENSDARG00000105243.2	Zebrafish
MamA	ZDB-GENE-091204-50	structural protein	ENSDARG00000109449.1	Zebrafish
MamA	ZDB-GENE-030131-2527	-	ENSDARG00000109553.1	Zebrafish
MamE	ZDB-GENE-090313-191	cytoskeletal protein	ENSDARG00000109814.1	Zebrafish
MamK	ZDB-GENE-040625-35	actin and actin related protein	ENSDARG00000110511.1	Zebrafish
MamA	ZDB-GENE-081031-100	protein modifying enzyme	ENSDARG00000110747.1	Zebrafish
Man6	ZDB-GENE-030131-731	-	ENSDARG00000111088.1	Zebrafish
MamK	ZDB-GENE-040625-35	actin and actin related protein	ENSDARG00000111417.1	Zebrafish
MamE	ZDB-GENE-081028-23	serine protease	ENSDARG00000111633.1	Zebrafish
MamH	ZDB-GENE-020228-1	transporter	ENSDARG00000111727.1	Zebrafish
MamE	ZDB-GENE-091113-30	serine protease	ENSDARG00000111992.1	Zebrafish
MamA	ZDB-GENE-030131-8173	primary active transporter	ENSDARG00000112054.1	Zebrafish
MamA	ZDB-GENE-040426-1169	-	ENSDARG00000113810.1	Zebrafish
MamK	ZDB-GENE-990415-92	-	ENSDARG00000114726.1	Zebrafish
MamE	ZDB-GENE-081028-32	serine protease	ENSDARG00000115120.1	Zebrafish
MamE	ZDB-GENE-040426-2544	cell junction protein	ENSDARG00000116591.1	Zebrafish
MamK	ZDB-GENE-040520-4	actin and actin related protein	ENSDARG00000116925.1	Zebrafish
